# Proteins Involved in Motility and Sperm-Egg Interaction Evolve More Rapidly in Mouse Spermatozoa

**DOI:** 10.1371/journal.pone.0091302

**Published:** 2014-03-07

**Authors:** Alberto Vicens, Lena Lüke, Eduardo R. S. Roldan

**Affiliations:** Reproductive Ecology and Biology Group, Museo Nacional de Ciencias Naturales (CSIC), Madrid, Spain; University of Hawaii at Manoa, John A. Burns School of Medicine, United States of America

## Abstract

Proteomic studies of spermatozoa have identified a large catalog of integral sperm proteins. Rapid evolution of these proteins may underlie adaptive changes of sperm traits involved in different events leading to fertilization, although the selective forces underlying such rapid evolution are not well understood. A variety of selective forces may differentially affect several steps ending in fertilization, thus resulting in a compartmentalized adaptation of sperm proteins. Here we analyzed the evolution of genes associated to various events in the sperm’s life, from sperm formation to sperm-egg interaction. Evolutionary analyses were performed on gene sequences from 17 mouse strains whose genomes have been sequenced. Four of these are derived from wild *Mus musculus, M. domesticus, M. castaneus* and *M. spretus*. We found a higher proportion of genes exhibiting a signature of positive selection among those related to sperm motility and sperm-egg interaction. Furthermore, sperm proteins involved in sperm-egg interaction exhibited accelerated evolution in comparison to those involved in other events. Thus, we identified a large set of candidate proteins for future comparative analyses of genotype-phenotype associations in spermatozoa of species subjected to different sexual selection pressures. Adaptive evolution of proteins involved in motility could be driven by sperm competition, since this selective force is known to increase the proportion of motile sperm and their swimming velocity. On the other hand, sperm proteins involved in gamete interaction could be coevolving with their egg partners through episodes of sexual selection or sexual conflict resulting in species-specific sperm-egg interactions and barriers preventing interspecies fertilization.

## Introduction

Sexual reproduction is a fundamental biological process common among eukaryotes. At the molecular level, reproduction is an intricate process that involves interactions between many proteins. Because of the significance of such proteins to fitness, their diversity and divergence in relation to many steps of the reproductive process suggests a role of adaptive diversification during the evolution of reproduction. Numerous studies have shown a rapid evolution of these so-called reproductive proteins and, thus, the idea that genes involved in reproductive processes evolve rapidly has gained widespread acceptance [1]–[4]. However, recent genomic and proteomic screens have revealed that the evolutionary pattern of reproduction-related proteins is more heterogeneous than previously assumed [5], [6]. Rates of molecular evolution may vary depending on the different steps of the reproductive process, timing of gene expression, and the tissue or organ in which genes are expressed [7]–[9]. Heterogeneity in evolutionary rates has even been found in the mature sperm proteome and, in fact, most sperm proteins seem to be under functional constraints and only a small subset appears to evolve rapidly [10]. Controversial hypotheses also exist regarding proteins with testis-specific expression that evolve more rapidly than proteins with expression in other tissues [10], [11].

Spermatozoa differentiate in the testis during spermatogenesis but cells released from the testis are not yet ready to engage in fertilization (reviewed in [12]). They acquire the ability to express motility during their transit through different portions of the epididymis. Sperm are stored in the distant part of the caudal epididymis where they remain quiescent. Upon ejaculation, and mixing with secretions from accessory glands, sperm motility is activated. During this process, proteins from accessory gland secretions may adhere to spermatozoa and modulate their function. Subsequently, sperm migrate in the female reproductive tract, actively overcoming barriers such as the cervix and the utero-tubal junction, until they reach the lower segments of the oviduct. There, sperm associate with the oviductal epithelium and undergo a series of molecular and cellular changes (collectively known as “capacitation”) which are essential for their subsequent interaction with the female gamete. During interaction with the egg, spermatozoa undergo an exocytotic process (the “acrosome reaction”), in response to egg-derived signals, and engage in sperm-egg recognition events before penetrating the extracellular egg coat, the zona pellucida. It is predictable that different proteins regulate the various steps in the life of the sperm cell that are required before and during fertilization.

Spermatozoa therefore represent an excellent model for protein compositional analysis in a highly differentiated cell. Such analyses are particularly relevant to our knowledge of the molecular mechanisms of fertilization and the underlying selective forces contributing to sperm form and function but, also, to understanding cell function in general. Comparative genomic analyses of sperm proteins are required to uncover features of sperm evolution and to identify candidate components that may be targeted by selection. Current proteomic research is identifying a growing number of proteins present in the sperm cell of several model species [13]–[16]. However, there are only a limited number of studies focusing on the evolution of mammalian sperm proteins from a global perspective [2], [10]. Recent proteomic analysis revealed an evolutionary acceleration of cell membrane genes relative to other genes in mouse sperm [10]. These results suggest that a compartmentalized adaptation of the sperm proteome may occur in response to different evolutionary forces [5], [13]. In fact, different selective forces may exert selective pressures on different steps in the life of the sperm cell. For instance, sperm could be selected by sperm competition to achieve faster and more efficient swimming velocity and quick penetration of the egg’s envelope [17], or sperm proteins could evolve rapidly in a sexual conflict scenario to adapt to changes in the egg surface promoting prevention of polyspermy [18].

Changes in sperm traits could be the result of the rapid evolution of genes regulating these traits [1]. Indeed, evidence for adaptive evolution of a few genes coding sperm proteins involved in several steps leading to fertilization has been presented [3]. Thus, protein compositional analyses relative to the role of proteins during the different events leading to fertilization may provide not only localized signals of positive selection but also clues as to which steps in this train of events are under higher selective pressure. We therefore investigated evolutionary rates of genes linked to different steps in the series leading to and including sperm-egg interaction. To this end we compiled a large dataset of sperm genes with a clear role in fertilization-related events and examined the extent of positive selection on such genes. Evolutionary analyses were conducted using gene sequences from 17 strains and species of mice, four of which represent wild-derived species (*Mus musculus musculus, M. m. domesticus, M. m. castaneus* and *M. spretus*) [19]. The recent sequencing of their genomes allows for the characterization of mouse sequence diversity and facilitates studies relating genotype to phenotype. Moreover, the close phylogenetic proximity and the high number of mouse lineages for which information is available meet the requirements for an accurate and powerful analysis to assess molecular evolution [20], [21].

In this study, we analyzed the evolution of sperm genes taking into account their role during the events leading to fertilization. Genes were thus grouped based on several events in the life of spermatozoa, namely (1) spermatogenesis, (2) sperm metabolism, (3) sperm motility, (4) sperm capacitation, (5) acrosome reaction, and (6) sperm-egg interaction. We focused on whether there is accelerated evolution of genes in any of these groups. We hypothesized that there was likely to be more rapidly evolving genes, and a higher proportion of proteins subjected to positive selection, in the group including sperm-egg interaction genes. This was based on earlier observations that identified intensified selection in mouse sperm membrane genes [10], that some sperm surface proteins involved in gamete recognition appear to evolve rapidly in other taxa [2], [22]–[26], and that strong species-specificity exists in gamete interaction [27] probably as a result of coevolution between male and female interacting proteins.

## Results

### Distribution of Sperm Proteins

A dataset of 1,350 proteins was gathered based on evidence of their presence in mouse spermatozoa (Table S1). From this dataset, 165 proteins were identified based on an established or strongly supported implication in the train of events culminating in fertilization (Table S2). A total of 33 proteins (20%) were found to be related to spermatogenesis, 23 (13.9%) to sperm metabolism, 34 (20.6%) to sperm motility, 21 (12.7%) to sperm capacitation, 20 (12.1%) to the acrosome reaction, and 34 (20.6%) to sperm-egg interaction. These proteins did not show a bias in the distribution of functional classes with respect to the initial dataset (χ^2^_d.f = 16_ = 0.898, p = 0.997).

### Evolutionary Rate of Sperm Genes

**Figure 1:**
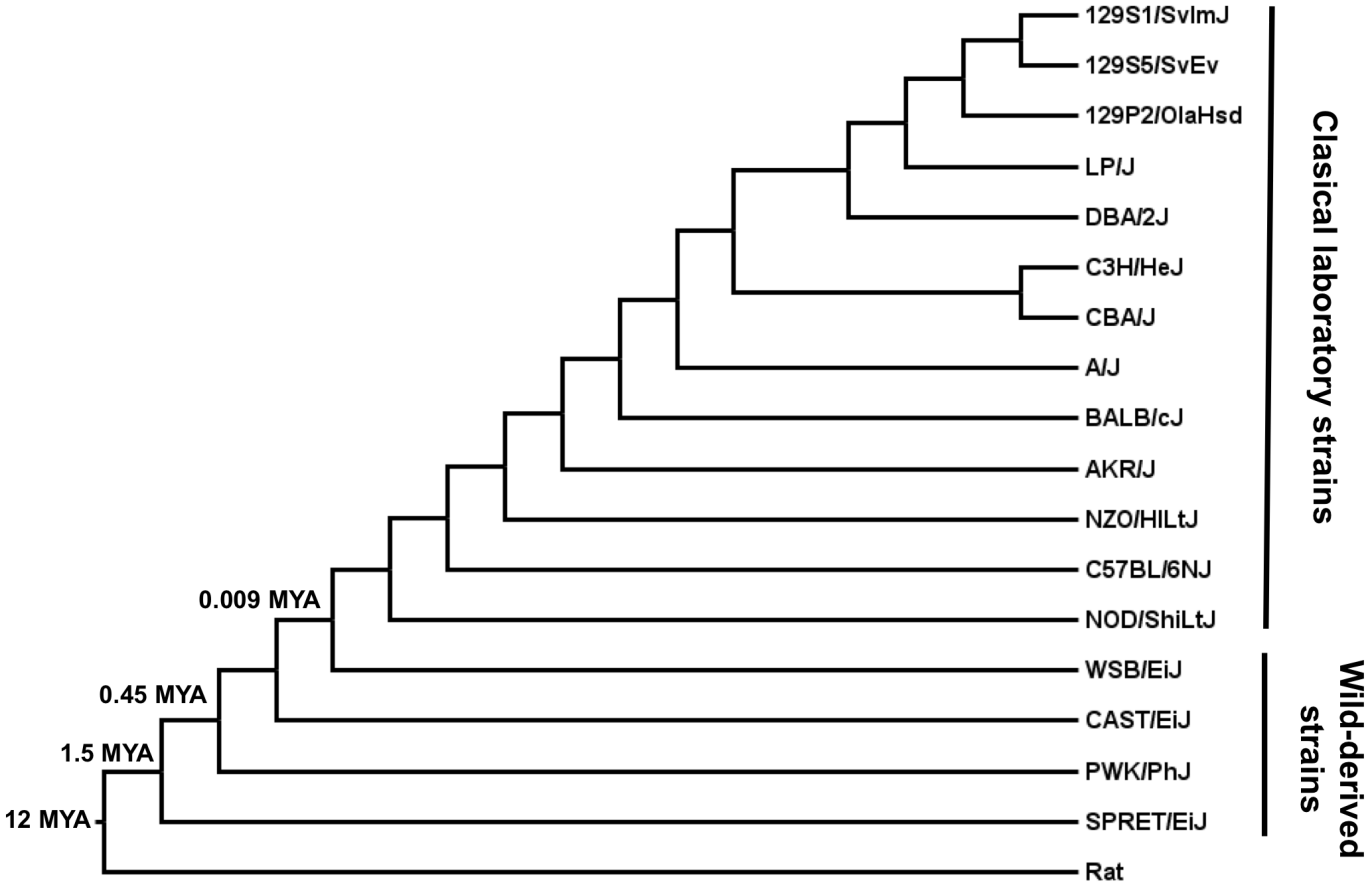
Phylogeny of mouse strains. Cladogram of mouse strains analyzed in this study. Divergence time in millions of years (MYA) is shown for splits among wild-derived strains and that between *Mus m. domesticus-*derived strain (WSB/Eij) and common inbred strains. On the right, the clades of classical laboratory strains and the wild-derived strains are indicated. *Rattus norvegicus* (rat) was used as outgroup. Phylogeny was built based on data from the literature [19], [62]–[64].

**Figure 2:**
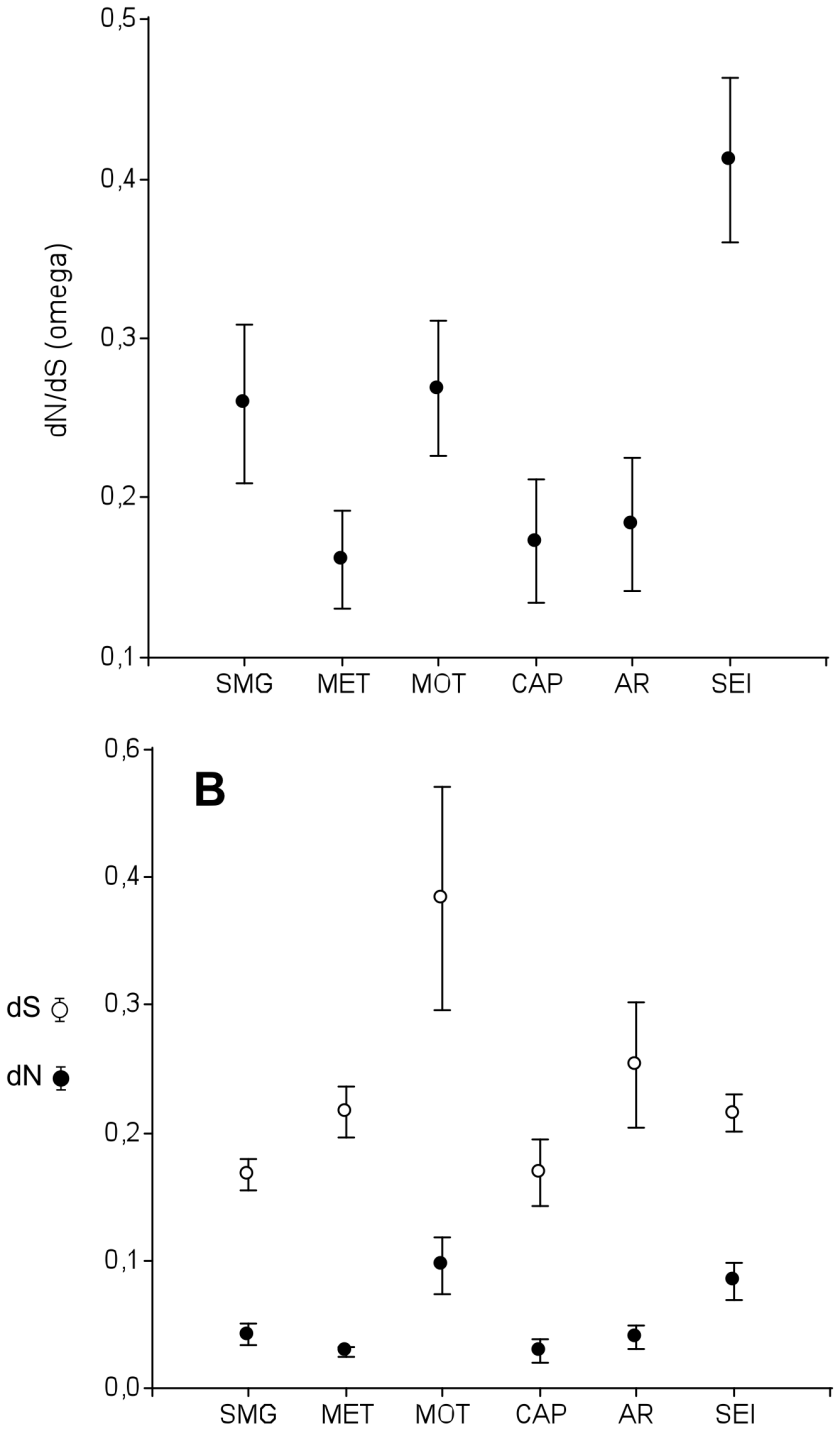
Evolution of mouse sperm genes grouped according to different events in the life of spermatozoa from sperm formation to sperm-egg interaction. (**A**) Average omega ratios (ω) and (**B**) Average nonsynonymous (dN, solid circles) and synonymous (dS, open circles) substitutions for proteins involved in spermatogenesis (SMG), sperm metabolism (MET), sperm motility (MOT), sperm capacitation (CAP), acrosome reaction (AR) and sperm-egg interaction (SEI). Standard errors are shown.

Nucleotide sequences of genes coding for the 165 selected sperm proteins were obtained from the genomes of 17 mouse strains and species (see Materials and Methods for details) (Fig. 1). We calculated the divergence for sperm genes as the ratio of nonsynonymous and synonymous substitutions (dN/dS = ω). Values of ω = 1 are expected for neutrally evolving genes whereas ω <1 indicates evidence of purifying selection, and ω >1 is considered a signal for positive selection. Estimation of evolutionary parameters among mouse strains and species resulted in an average dN of 0.064 (S.D. = 0.089), an average dS of 0.2630 (S.D. = 0.2902) and an average ω of 0.259 (S.D. = 0.255). Most proteins showed ω ratios between 0 and 1 (Table S3) and values greater than 1 were obtained for 6 sperm proteins (Tmem190, Hils1, Smcp, Slxl1, and Fscb and Crisp1) although only slightly above 1 (Table S3). Distributions of evolutionary rates and ω ratios were compared between the different proteins grouped according to their involvement in different events leading to fertilization. A higher average ω ratio was observed for proteins involved in sperm-egg interaction (ω = 0.413, S.D. = 0.304) in comparison to those in other groups. Proteins related to sperm metabolism (ω = 0.156, S.D. = 0.14), sperm capacitation (ω = 0.168, S.D. = 0.181) and the acrosome reaction (ω = 0.178, S.D. = 0.186) showed the lowest average ω values. Proteins with a role in spermatogenesis (ω = 0.262, S.D. = 0.287) and sperm motility (ω = 0.266, S.D. = 0.247) exhibited intermediate averages of ω (Fig. 2A). Statistical analyses revealed significant differences among ω values of the different protein groups (H = 23.18, p = 0.0003). Multiple comparison tests among groups detected significant differences in ω ratios of sperm-egg interaction proteins in relation to the other groups with the exception of sperm motility (Table 1). This latter group did not show significant differences in any comparison.

**Table 1:** Multiple comparisons of omega values among reproductive groups.

Reproductive process	Spermatogenesis	Sperm metabolism	Sperm motility	Capacitation	Acrosome reaction	Sperm-egg interaction
Spermatogenesis	–	0.27	0.64	0.23	0.38	0.033 (*)
Sperm metabolism	–	–	0.12	0.90	0.86	0.0016 (**)
Sperm motility	–	–	–	0.10	0.20	0.12
Capacitation	–	–	–	–	0.77	0.0014 (**)
Acrosome reaction	–	–	–	–	–	0.0061 (**)
Sperm-egg interaction	–	–	–	–	–	–

Evolutionary rates (ω) of groups assembled based on different events in the life of the sperm cell before fertilization. *P*-value for each pairwise comparison is shown. (*) and (**) indicate statistical significance with a 95% and 99% confidence interval respectively.

Sperm motility proteins presented the highest average dN value (dN = 0.11, S.D. = 0.143), followed by proteins related to sperm-egg interaction (dN = 0.094, S.D. = 0.089). The lowest dN estimates corresponded to the conserved categories of sperm metabolism (dN = 0.033, S.D. = 0.022) and sperm capacitation (dN = 0.031, S.D. = 0.038) (Fig. 2B). Comparative analysis revealed significant differences for average dN among reproductive events (H = 28.29, p = 0.0002). Proteins involved in sperm-egg interaction showed an average dN rate significantly higher than those in the other groups except for sperm motility, which presented significant differences only with sperm capacitation (Table 2).

**Table 2:** Multiple comparisons of evolutionary rates among reproductive processes.

Reproductive process	Spermatogenesis	Sperm metabolism	Sperm motility	Capacitation	Acrosome reaction	Sperm-egg interaction
Spermatogenesis	–	0.88	0.06	0.30	0.86	0.0021 (**)
Sperm metabolism	0.05	–	0.06	0.64	0.54	0.0005 (**)
Sperm motility	0.002 (**)	0.15	–	0.028 (*)	0.08	0.13
Capacitation	0.75	0.25	0.025 (*)	–	0.96	0.0003 (**)
Acrosome reaction	0.05	0.93	0.18	0.21	–	0.024 (*)
Sperm-egg interaction	0.09	0.92	0.14	0.09	0.94	–

Average synonymous (dS) and non-synonymous (dN) substitutions were calculated for different groups. *P*-values above the diagonal represent pairwise comparisons of dN rates whereas *P*-values below the diagonal show comparisons of dS rates. (*) and (**) indicate statistical significance with a 95% and 99% confidence interval respectively.

Statistically significant differences were also detected for dS values among the different groups of reproductive events (H = 17.96, p = 0.003). Proteins in sperm motility (dS = 0.418, SD = 0.561) showed the highest average dS, but the data were very spread out resulting in a high standard deviation (Fig. 2B). Sperm motility differed significantly from those groups with lower dS values such as spermatogenesis (dS = 0.182, S.D. = 0.076) and sperm capacitation (dS = 0.191, S.D. = 0.128) (Table 2).

Previous studies have demonstrated that most genes with a ω >0.5 are likely to have undergone adaptive evolution [28] and such genes have been considered as positively selected. We therefore selected those genes showing ω >0.5 (Table S3) and carried out additional analyses. Twenty-five sperm genes were identified. Significant differences were revealed in the percentage of rapidly evolving genes in each group (χ^2^_d.f = 5_ = 22.2, p = 0.0005) among this subset of genes. Rapidly evolving genes associated with sperm-egg interaction were the most overrepresented (9 out of 25; 36%) (Table 3), with a significant deviation from the proportion expected (19.4%).

**Table 3:** Rapidly evolving sperm genes involved in different reproductive processes.

Reproductive process	Number of geneswith dN/dS >0.5	Proportion of geneswith dN/dS >0.5	Expected proportion ofgenes with dN/dS >0.5	Observed- expected
Spermatogenesis	6	24.0	20.0	4.0
Sperm metabolism	3	8.0	13.94	−5.94
Sperm motility	4	20.0	20.61	−0.61
Capacitation	1	4.0	12.73	−8.73
Acrosome reaction	2	8.0	12.12	−4.12
Sperm-egg interaction	9	36.0	20.61	15.39

The proportion of sperm genes with ω >0.5 is shown for each group. Expected proportions were calculated as the percentage of genes of each group in the total sample. Differences between observed and expected proportions are presented.

Because the inclusion of highly related mouse strains may reduce the level of nucleotide variation and, thus, limit the sensitivity of our evolutionary analyses, we repeated the analyses of evolutionary rates including only the strains that derive from wild-derived species, namely PWK/PhJ, WSB/EiJ CAST/EiJ and SPRET/EiJ, derived from *Mus musculus, M. domesticus, M. castaneus* and *M. spretus*, respectively. The results obtained were very similar to those obtained with previous analyses for all genes. Moreover, the statistical comparisons yielded identical results (not shown).

Overall, these results showed that the evolutionary rate of sperm genes is heterogeneous in relation to the events in which they participate in the sequence ending in fertilization, with proteins involved in sperm-egg interaction exhibiting an accelerated evolution.

### Positive Selection on Sperm Proteins

Although ω values (dN/dS) are important indicators of selective pressure at the protein level, many proteins have a high proportion of amino acids that may remain largely invariable due to strong functional constraints. Thus, adaptive evolution most likely occurs at a few amino acids across protein sequence [29]. In such cases, the ω ratio averaged over the entire sequence will not be significantly >1 even if adaptive molecular evolution has occurred. This limitation is even more drastic when very closely related taxa are analyzed, as the case of this study. To overcome this limitation, we used models that account for heterogeneous evolutionary rates among amino acid sites to test for positive selection [29].

**Figure 3:**
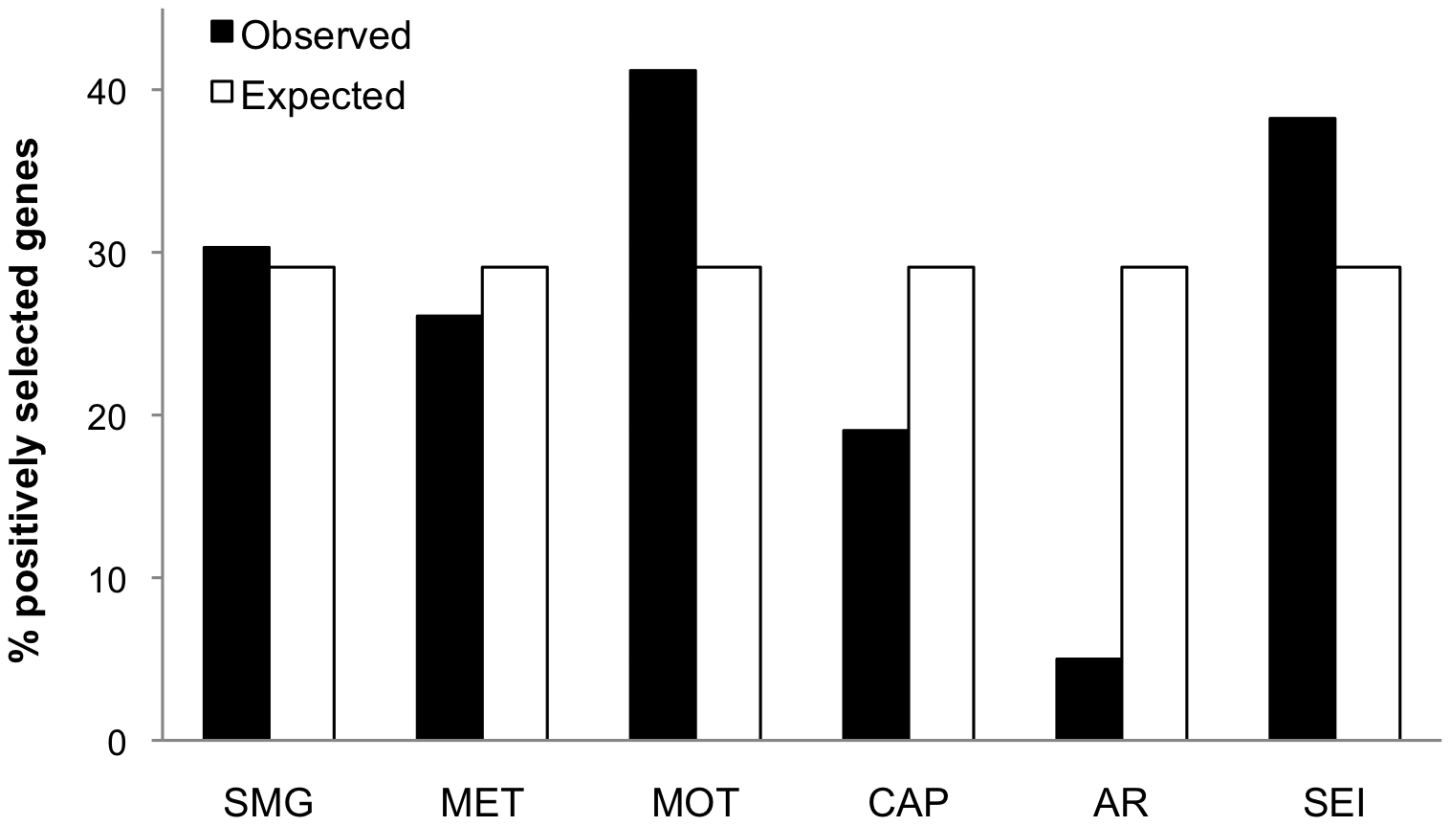
Positive selection on sperm genes involved in different events in the life of spermatozoa. Percentage of observed and expected genes subjected to positive selection in each class of sperm function. SMG: spermatogenesis, MET: sperm metabolism, MOT: sperm motility, CAP: sperm capacitation, AR: acrosome reaction, SEI: sperm-egg interaction. Expected proportion of positively selected genes was based on the percentage of all genes showing evidence for positive selection.

Site analysis applied to our dataset identified a total of 48 out of 165 (29.1%) sperm genes evolving under positive selection (Table S4). Different proportions of sperm genes under positive selection were observed among groups. A total of 10 positively selected genes were associated to spermatogenesis (out of 33 proteins in this group; 30.3%), 6 to sperm metabolism (out of 23; 26.1%), 14 to sperm motility (out of 34; 41.2%), 4 to capacitation (out of 21; 19.0%), 1 to acrosome reaction (out of 20; 5%) and 13 to sperm-egg interaction (out of 34; 38.2%) (Fig. 3).

The proportion of genes experiencing positive selection differed very significantly among the various groups (χ^2^_d.f = 5_ = 31.7, p = 6.9×10^−6^). Within this variant pattern, the groups of sperm motility and sperm-egg interaction exceeded the expected proportion of genes under positive selection (29.1%), estimated as the percentage of all genes exhibiting evidence of positive selection. On the other hand, sperm capacitation and acrosome reaction groups keep highly conserved genes with a low proportion of genes showing signature of positive selection. As done with the previous set of analyses, we applied these models only to strains that descend from wild-derived species to assess signatures of positive selection. Evidence of adaptive evolution was identified for the same set of genes, and statistical results remained unchanged.

**Figure 4:**
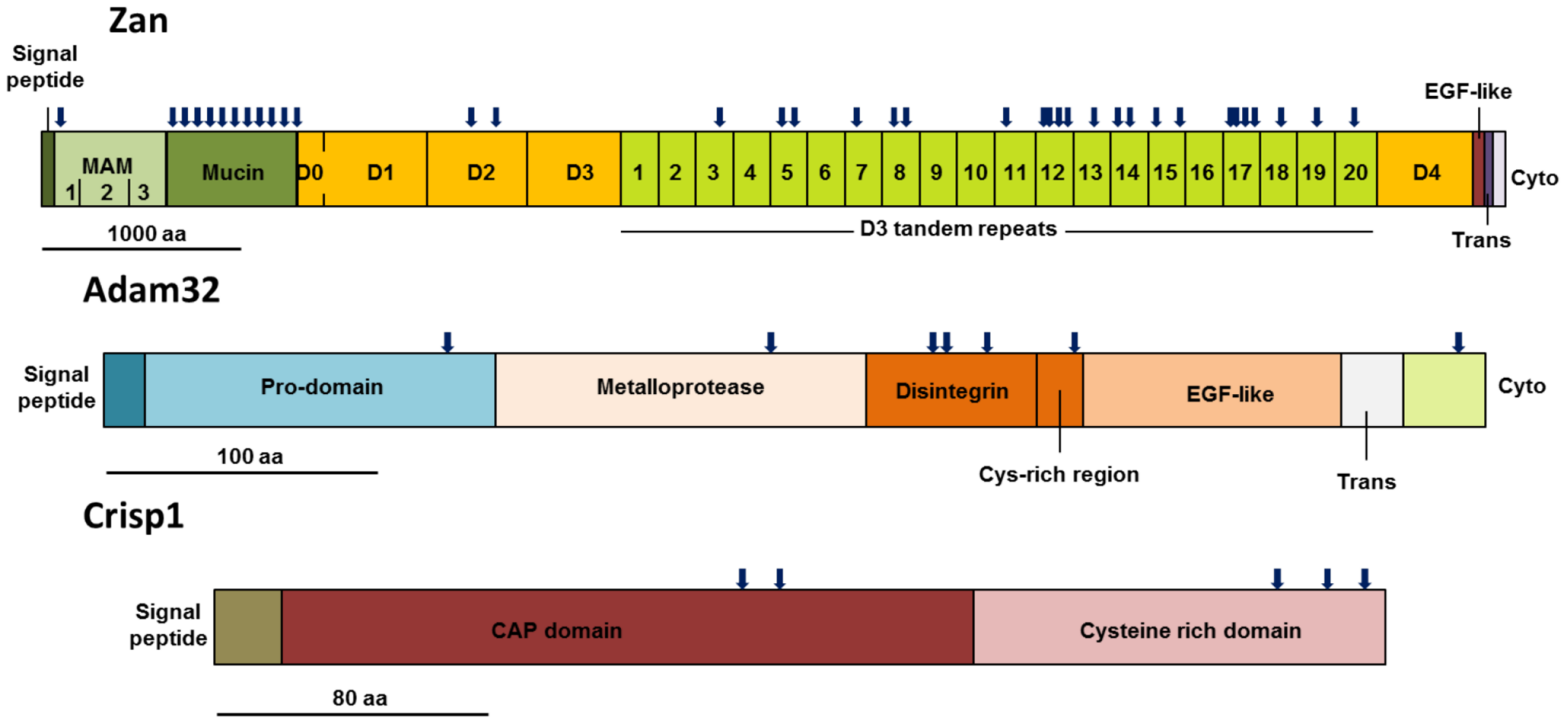
Schematic representation of the secondary structure of three sperm proteins subjected to positive selection. Positions of positively selected sites with a Bayesian posterior probability >0.95 are indicated with arrows. The relative scale in amino acids is shown for each protein. Domain organization was drawn based on information from UniProt.

Examples of sites under positive selection in three proteins involved in sperm-egg interaction (zonadhesin, Adam32 and Crisp1) are presented in Fig. 4. In zonadhesin, an intense signal of positive selection was detected in the mucin-like domain and a high number of positively selected sites were found along the partial D3 repeats generated by tandem duplication. In the case of Adam32, four out of the six codons under positive selection lie in the disintegrin/cysteine-rich adhesion domains, the region presumably involved in interacting with egg integrins. In Crisp1, the five positively selected codons were spread between the CAP domain and the C-terminal region of the cysteine-rich domain, whereas the 16 characteristic cysteine residues of the CRISP family were conserved.

Taken together, these results suggest that a heterogeneous adaptation also exists among the different groups of genes related to different events in the path to fertilization, and that genes related to sperm motility and sperm-egg interaction tend to experience a more intense positive selection.

## Discussion

The results of this study provide evidence that integral sperm proteins show a differential evolutionary rate depending on their role in steps leading to and including fertilization. Mouse sperm proteins with a putative role in sperm-egg interaction revealed an accelerated evolution, with an elevated proportion of proteins exhibiting positive selection. In addition, we detected a high number of proteins involved in sperm motility containing residues subjected to putative positive selection. Proteins related to other events in the sequence leading to fertilization did not exhibit such accelerated evolution nor intensified selection.

Previous studies in *Drosophila* and the mouse have led to the hypotheses that differential selection across sperm cells results in the compartmentalization of adaptation in different sperm components or subcellular domains, and that such compartmentalized adaptation could take place in response to sexual selection [10], [13]. As shown in this study, a compartmentalization seems to arise in connection to the role of proteins in different events taking place during the life of the sperm cell. Taken together, results from previous work and the current study, suggest that both a subcellular and a functional compartmentalization exists in spermatozoa, most likely as a response to multiple selective forces acting differentially on the sperm cell.

From an initial dataset of 1,350 mouse sperm proteins identified in previous analyses of the sperm proteome, we concentrated on a subset of proteins whose function has been clearly associated to the train of events ending in fertilization. A total of 165 sperm proteins were identified in this subset and they were categorized in six groups according to their involvement in the main steps in the life of spermatozoa that span from their formation in the testis to their interaction with the female gamete. A drawback of assigning genes to individual groups is that some proteins are now clearly associated to more than one sperm function. One example is the sperm surface protein PKDREJ, originally thought to be involved in the interaction with the egg zona pellucida, but recently characterized as a possible regulator of the acrosome reaction [25], [30]. Another example is the soluble adenylyl cyclase (Sacy), an essential component of cAMP-signaling cascades that activates both sperm motility and sperm capacitation [31]. On the other hand, the allocation of a protein to multiple groups would have violated the principle of independency between categories required for statistical comparisons. Thus, we assigned each protein to a single group based on the preponderance of evidence for a given sperm function. To verify that initial assignments did not influenced the outcome of analyses, we repeated the tests after changing the group to which multifunction proteins were assigned. The same evolutionary trends were observed and, thus, we conclude that our results are robust.

Mouse sperm genes with a putative role in sperm-egg interaction exhibited clear evidence of accelerated evolution. In addition, an elevated proportion of genes under positive selection were detected in this group. Some of the most rapidly evolving genes identified in this group are known to be either essential or important in binding, penetration and fusion with the egg.

Interactions between sperm and egg must be maintained through a coevolutionary process, whereby rapid evolution of a protein requires rapid changes in the other partner. The coevolution of interacting female and male proteins could contribute to the establishment of barriers to fertilization, leading to reproductive isolation of populations and consequently to the formation of new species [1]. The process of coevolution of gamete interacting proteins has been described in detail for free-spawning marine invertebrates [32], [33]. Our results for closely related mouse strains and species suggest that the rapid divergence of gamete interacting proteins is also a feature of the evolution of reproductive systems in internally fertilizing species. Among species with internal fertilization, such as mammals, in which sperm traverse the female reproductive tract before reaching the egg, there are additional events in the life of the sperm cell including the need to negotiate several barriers and a suite of molecular changes that prepare spermatozoa to interact with the egg. Thus, in species with internal fertilization, sperm traits other than those linked to the ability to bind and interact with the egg could also be subjected to higher selective pressures.

The driving force promoting such rapid divergence of sperm-egg interaction proteins remains unknown. Several selective forces, such as sperm competition, cryptic female choice, reinforcement, or sexual conflict, have been proposed [34]–[37]. In mammals, in which polyandry is a common mating system, sperm competition may represent a powerful selective force shaping reproductive processes [38]. Sperm competition may drive evolution of sperm proteins towards conditions that are optimal for males, but these may present disadvantages for female fitness due to polyspermy (i.e., fertilization by more than one sperm, which is lethal in many taxa). Sexual conflict between adaptive optima is then thought to lead females and males to counter-adapt, creating a characteristic coevolutionary antagonism between male and female traits [37]. This may be the case of positive selection in the mammalian egg coat proteins ZP2 and ZP3, which are involved in blocking the binding of multiple sperm and thus prevent polyspermy [39], [40]. Altogether, it is possible that proteins involved in sperm-egg interaction may experience both concerted evolution, thus explaining specificity in gamete interaction, which is most clear in interspecfic crosses in which there is strong conspecific sperm precedence [41], [42] and, concomitantly, antagonistic coevolution linked to the prevention of polyspermy [27]. Future functional studies focused on the identification of signatures of coevolution on pairs of sperm and egg interacting proteins will be required to understand the evolutionary forces driving divergence in fertilization proteins in mammals, as has been previously done for invertebrates [33].

The results of our study agree with earlier findings in which some proteins mediating gamete interaction were found to evolve rapidly [2], [22]. The molecular evolution of the ADAM gene family has been examined in primates, and sites under positive selection were identified in the disintegrin/cysteine-rich adhesion domains (the region presumably binding to integrins in the egg membrane) of sperm surface proteins ADAM2 and ADAM32 [43]. In mice, we observed positively selected sites concentrated in this region in ADAM 32, supporting the idea that selection on reproductive ADAMs is driven by male-female interactions. Zonadhesin, a sperm-specific protein implicated in zona pellucida binding, has been analyzed in different mammalian groups. In the mouse, a strong signal of positive selection was observed in the D3 tandem repeats [44], a result that was also obtained in our study examining several mouse strains and species. The rapid evolution of these zonadhesin duplicated regions may contribute to species-specificity in rodents [45].

An intensified signal of positive selection in proteins related to sperm motility was also identified. Sperm motility is important to ensure fertilization and the velocity at which sperm swim is a key factor for male reproductive success as the first sperm cell to reach the ovum is more likely to engage in fertilization [12]. Postcopulatory selective forces such as sperm competition promote increases in sperm swimming velocity in many taxa [46], [47] and such increases may be achieved by changes in sperm design [17], [48], [49]. Nonetheless, in addition to these phenotypic adaptations, it is possible that increases in sperm swimming velocity may be also achieved by adaptive molecular changes in flagellar proteins participating in sperm motility.

A previous study comparing mouse with distant species of mammals identified that a set of proteins related to sperm motility exhibit a signature of positive selection [10]. Our analyses on mouse strains identified residues under positive selection in a variety of proteins involved in sperm motility with diverse molecular functions. They include protein kinases (Smky, Smok2a, Smok2b), cation channels (Atp1a4, Nhe5), motor dyneins (Tctex5) and structural proteins of the flagellar microtubules (Tekt4). These proteins also showed a wide distribution across the sperm flagellum; positively selected proteins are located either in the midpiece or in the principal piece, as well as in different subcellular structures of the sperm flagellum (axoneme, outer dense fibers, mitochondrial and fibrous sheat). Furthermore, signature of positive selection was observed both in proteins involved in activated motility (as mentioned above) and also in those regulating hyperactivated sperm motility, such as Catsper2 and Catsper3 [50]. Therefore, the enhanced signal of positive selection in proteins sharing a role in sperm motility might be a response at the molecular level to the pressure of sperm competition on sperm swimming velocity as an adaptive trait in fertilization success. Future molecular and genomic approaches may serve to functionally characterize proteins involved in sperm motility and to test the evolutionary forces that act on them.

Despite the intensified positive selection in proteins related to sperm motility, it was somewhat surprising to observe a high degree of conservation in the group of proteins involved in sperm metabolism. This can be so because metabolic pathways are highly conserved and because ATP generated in the sperm flagellum is essential for multiple cellular and biochemical processes regardless of sperm motility, such as protein phosphorylation, ion regulation, capacitation and acrosome reaction [51]. Nonetheless, 3 out of 6 metabolic genes showing evidence of positive selection were *G6pdh2*, *Gapdhs* and *Pgk2*, which are isoforms of glycolytic enzymes expressed only in spermatogenic cells [52]–[54]. This suggests that metabolic proteins with unique expression in sperm could fix adaptive changes in order to improve the efficiency of ATP production and supply a higher energy repository for motility generation. Indeed, recent work has demonstrated that increases in sperm competition are associated with higher sperm ATP content in muroid rodents, and that ATP content is linked to increases in sperm swimming velocity [55].

In earlier studies, evolutionary rates of mouse sperm genes have been estimated through sequence comparisons with rat and other distant mammalian orthologs [10]. The use of mouse strains and closely related species, as done in our study, provides a more suitable model to develop evolutionary analyses [20]. Our work showed similar results when all strains and species were included and when only strains derived from wild species were considered. Therefore, analyses of mouse strains did not bias the estimation of synonymous and nonsynonymous substitutions in codon-based analyses and these strains may prove useful in future studies of genomic variation and its phenotypic effects [19].

In any case, it should be borne in mind that, despite finding clear differences in proteins associated to different reproductive processes, the strains and species analyzed have not necessarily evolved under the influence of sexual selection. Thus, to assess the impact of selective forces such as sperm competition or sexual conflict on the evolution of sperm proteins, future studies could examine the existence of positive selection of candidate proteins identified in this study among species with varying levels of promiscuity. This approach will most likely yield relevant information because analyses of a small subset of sperm proteins have shown a relationship between their evolution and the intensity of sperm competition in mammals [43], [56], [57].

In conclusion, our study has shown that many integral sperm proteins involved in sperm-egg interaction experience accelerated evolution in the mouse and that a high proportion of these proteins undergo positive selection. Furthermore, our comparative analyses revealed an enhanced positive selection on proteins with a putative role in sperm motility. In contrast, a lower proportion of proteins participating in other events in the sequence leading to fertilization exhibited a signature of adaptive evolution. Nonetheless, this fact does not imply that processes such as spermatogenesis, sperm capacitation or acrosome reaction are not important in terms of sperm evolution since this and other studies have revealed evidence of adaptive evolution in proteins associated with such processes [2], [10], [58]. Mouse strains analyzed in the present work, including some recently derived from wild species, seem to be a good model in comparative genomic studies of the so-called reproductive genes, as well as of genes expressed in other tissues. The rapidly evolving sperm genes identified in this study will serve as candidates for future studies on genotype-phenotype relationships of reproductive processes. In addition, our identification of amino acid sites subjected to positive selection in a large set of reproductive proteins may shed light on regions that are important for fertilization. Empirical data about the functional effects of changes in amino acids under selection in reproductive proteins is still very scarce. Future analyses characterizing the functionality of such protein regions and their importance in fertility will be useful to reveal the adaptive significance of changes occurring in sperm proteins.

## Materials and Methods

### Selection of Sperm Proteins

An initial dataset comprising 1,350 proteins was gathered based on evidence of their presence in mouse sperm (Table S1). Data were collected from previously published mouse sperm proteomes [10], [14], [59], [60] as well as a comprehensive review of the literature. Within this dataset, a total of 165 proteins were selected for analyses based on a confirmed or strongly supported association of these sperm proteins to an event in the sequence leading to and including fertilization (Table S2). Information regarding the roles of these proteins in sperm was collected from different resources, namely UniProt (http://www.uniprot.org), Mouse Genome Informatics (http://www.informaticsjax.org), and Gene Ontology annotations (AmiGO, http://www.geneontology.org) as well as a critical review of the literature. Six groups were established according to the different cellular processes in which the proteins are involved: spermatogenesis, sperm metabolism, sperm motility, sperm capacitation, acrosome reaction, and sperm-egg interaction. Each protein was assigned to a single group to guarantee the independency between categories. Proteins which have been associated to more than one reproductive event were placed in the group with stronger evidence for their functions (Table S2). Proteins involved in cell defense or immunity were not included in this study because, despite the fact that they can respond to selection associated with sperm functions [61], detection of positive selection in a set of these proteins could be due to a response to host defense mechanism rather than to sexual selection. Seminal fluid proteins and epydidimal proteins were also not included in this study because we focused exclusively on integral sperm proteins.

### Mouse Genome Sequences

Nucleotide sequences of genes coding for the selected sperm proteins were extracted from the genomes of 17 mouse strains and species. The genomes include those of the classic laboratory strains (1295SvEv, 129P2/OlaHsd, 129S1/SvImJ, C3H/HeJ, CBA/J, A/J, AKR/J, DBA/2J, LP/J, BALB/cJ, NZO/HlLtJ and NOD/ShiLtJ), and those of four wild-derived strains (PWK/PhJ, WSB/EiJ CAST/EiJ and SPRET/EiJ) which include the progenitors of common laboratory strains and represent *Mus musculus musculus, M. m. domesticus, M. m. castaneus* and *M. spretus*, respectively. Whole-genome sequences were downloaded in FASTA format from the FTP server of Mouse Genomes Project of the Sanger Institute (http://www.sanger.ac.uk) [19]. Transcript sequences were extracted in tandem from whole genome files using 5′ and 3′ terminal fragments as queries in searches using text processing software. Transcript queries were retrieved from *Mus musculus* using the BioMart tool (http://www.ensembl.org/). Introns and UTRs were removed from transcript sequences based on Ensembl information and exons were assembled to profile the protein-coding sequences using the sequence alignment editor BioEdit [62]. Correct assembling was checked comparing the strains extracted sequences to the corresponding coding sequence for *M. musculus* registered in the nucleotide database of NCBI (http://www.ncbi.nlm.nih.gov). Coding sequences for *Rattus norvegicus* were also obtained from NCBI nucleotide database. Coding sequences were aligned in-frame using ClustalW implemented in BioEdit [62].

### Evolutionary Analysis

Evolutionary analyses were conducted on 165 sperm gene sequences in 17 mouse strains and species using the Codeml program implemented in the PAML4 package [63]. A phylogenetic tree of the mouse strains analyzed (Fig. 1) was constructed based on previously published genealogies and phylogenies for these strains [19], [64]–[66]. Nucleotide alignments of the coding sequences and the reconstructed phylogenetic tree, when required, were used as inputs in evolutionary analyses. *Rattus norvegicus* was used as outgroup. The evolutionary rate was estimated as the nonsynonymous/synonymous substitution ratio (dN/dS), resulting in the omega parameter (ω). Average dN, dS and ω values were estimated through the whole alignment using the basic model of PAML implemented in Codeml [63]. To test for positive selection, likelihood site models were compared using Codeml. The selection model M8 (assuming a beta distribution for ω with values greater than 1) was compared with the null hypothesis, M8a (with ω_s_ fixed to 1). The likelihood ratio test (LRT) comparing M8-M8a was carried out with the 50∶50 mixture of point mass 0 and χ^2^ with a degree of freedom. We established a conservative significance level of p = 0.01 to reduce the number of false positives to 1% of total number of genes showing a signal positive selection. When the LRT was significant, it implied that selection models showed a better fit and thus positive selection could be inferred. For those genes undergoing positive selection, Bayes empirical Bayes (BEB) analyses were performed to identify positively selected residues with a BEB posterior probability >95%.

### Statistical Analysis

Shapiro-Wilks normality tests were conducted to determine whether the evolutionary parameters (dN, dS, ω) were modeled by a normal distribution using InfoStat software (http://www.infostat.com.ar). Statistical comparisons between distributions of dN, dS and ω estimated for the different groups of reproductive processes were conducted using the non-parametric Kruskal-Wallis test (InfoStat). Multiple testing correction for pairwise comparisons were carried out applying the *kruskalmc* function [67] implemented in the package Pgirmess for the statistical software R [68]. Proportions of both rapidly evolving genes and genes under putative positive selection in each group were compared to expected values applying a chi-square test with 5 degrees of freedom and a threshold of p = 0.01. Expected values were calculated as the proportion of proteins of each group with regards to the total number of proteins.

## Supporting Information

Table S1 Lists of compiled proteins.
(PDF)

Table S2 List of proteins used in the study.
(PDF)

Table S3 Values of non-synonymous (dN), synonymous (dS) and dN/dS (ω) ratios estimated for sperm genes. Sperm genes with a dN/dS ratio >0.5 are shown in bold.
(PDF)

Table S4 Analysis of positive selection in sperm genes. Results of likelihood ratio test (LRT) comparing the likelihood values obtained in models M8 and M8a are presented. Evidence of positive selection is detected as the comparison of the LRT with the 50∶50 mixture of point mass 0 and χ2 is greater than the critical value 5.41 at 1% of significance. Infered positively selected sites with BEB posterior probabilities >0.95 (*) and >0.99 (**) are shown.
(PDF)
