# Supplementary material for: Proteins Involved in Motility and Sperm-Egg Interaction Evolve More Rapidly in Mouse Spermatozoa
Source: PLoS One. 2014 Mar 7;9(3):e91302. doi: 10.1371/journal.pone.0091302 (PMC3948348; doi:10.1371/journal.pone.0091302)
Supplement: Table S1 — Lists of compiled proteins. (PDF) [file pone.0091302.s001.pdf]

Table S1. Lists of compiled proteins.

Proteins from previous mouse sperm proteomes		
**Protein Symbol**	**Protein Name**	**Reference**
0710001P09 Rik	coiled-coil-helix-coiled-coil-helix domain containing 6	[2]
1100001H23Rik	phospholipase B domain containing 1	[1]
1110017D15Rik	RIKEN cDNA 1110017D15 gene	[2]
1110020P15Rik	RIKEN cDNA 1110020P15 gene	[2]
1190002A17Rik	RIKEN cDNA 1190002A17 gene	[2]
1500001M20Rik	RIKEN cDNA 1500001M20 gene	[2]
1600016N20Rik	RIKEN cDNA 1600016N20 gene	[2]
1700001L19Rik	RIKEN cDNA 1700001L19 gene	[2]
1700001O22Rik	RIKEN cDNA 1700001O22 gene	[2]
1700007K13Rik	RIKEN cDNA 1700007K13 gene	[2]
1700009P17Rik	RIKEN cDNA 1700009P17 gene	[2]
1700011F03Rik	RIKEN cDNA 1700011F03 gene	[2]
1700013F07Rik	RIKEN cDNA 1700013F07 gene	[2]
1700014D04Rik	RIKEN cDNA 1700014D04 gene	[2]
1700018L24Rik	RIKEN cDNA 1700018L24 gene	[1],[2]
1700019B03Rik	RIKEN cDNA 1700019B03 gene	[2]
1700019D03Rik	RIKEN cDNA 1700019D03 gene	[2]
1700019L03Rik	RIKEN cDNA 1700019L03 gene	[2]
1700019N19Rik	RIKEN cDNA 1700019N19 gene	[2]
1700019O17Rik	RIKEN cDNA 1700019O17 gene	[2]
1700020K04Rik	RIKEN cDNA 1700020K04 gene	[2]
1700021F05Rik	RIKEN cDNA 1700021F05 gene	[2]
1700022A21Rik	RIKEN cDNA 1700022A21 gene	[2]
1700023D08Rik	RIKEN cDNA 1700023D08 gene	[2]
1700023E05Rik	RIKEN cDNA 1700023E05 gene	[2]
1700024P04Rik	RIKEN cDNA 1700024P04 gene	[2]
1700026D08Rik	RIKEN cDNA 1700026D08 gene	[2]
1700026L06Rik	RIKEN cDNA 1700026L06 gene	[2]
1700027A15Rik	RIKEN cDNA 1700027A15 gene	[2]
1700027D21Rik	RIKEN cDNA 1700027D21 gene	[2]
1700034I23Rik	RIKEN cDNA 1700034I23 gene	[2]
1700049K14Rik	RIKEN cDNA 1700049K14 gene	[2]

Proteins from previous mouse sperm proteomes		
**Protein Symbol**	**Protein Name**	**Reference**
1700055M20Rik	testis, prostate and placenta expressed	[2]
1700058C13Rik	RIKEN cDNA 1700058C13 gene	[2]
1700061G19Rik	RIKEN cDNA 1700061G19 gene	[2]
1700065I17Rik	RIKEN cDNA 1700065I17 gene	[2]
1700071K01Rik	RIKEN cDNA 1700071K01 gene	[2]
1700080O16Rik	RIKEN cDNA 1700080O16 gene	[2]
1700081D17Rik	phosphatidylethanolamine binding protein 4	[1]
1700101E01Rik	RIKEN cDNA 1700101E01 gene	[2]
1700110M21Rik	RIKEN cDNA 1700110M21 gene	[2]
1700112C13Rik	RIKEN cDNA 1700112C13 gene	[1],[2]
1700113O17Rik	RIKEN cDNA 1700113O17 gene	[2]
2810002D13Rik	leucine rich repeat containing 57	[2]
2810408A11Rik	RIKEN cDNA 2810408A11 gene	[2]
4732415M23Rik	RIKEN cDNA 4732415M23 gene	[2]
4921507P07Rik	RIKEN cDNA 4921507P07 gene	[2]
4921517D21Rik	RIKEN cDNA 4921517D21 gene	[2]
4921523A10Rik	RIKEN cDNA 4921523A10 gene	[2]
4921524E03Rik	aldolase 1, A isoform, retrogene 1	[2]
4921530D09Rik	RIKEN cDNA 4921530D09 gene	[2]
4930404N11Rik	RIKEN cDNA 4930404N11 gene	[2]
4930408G06Rik	RIKEN cDNA 4930408G06 gene	[2]
4930415O20Rik	RIKEN cDNA 4930415O20 gene	[2]
4930435E12Rik	RIKEN cDNA 4930435E12 gene	[2]
4930443G12Rik	RIKEN cDNA 4930443G12 gene	[2]
4930511I11Rik	RIKEN cDNA 4930511I11 gene	[2],[3]
4930519F16Rik	RIKEN cDNA 4930519F16 gene	[2]
4930522H14Rik	RIKEN cDNA 4930522H14 gene	[2]
4930523C11Rik	a disintegrin and metallopeptidase domain 6B	[1],[2]
4930550C14Rik	RIKEN cDNA 4930550C14 gene	[2]
4930555I21Rik	RIKEN cDNA 4930555I21 gene	[2]
4930579C15Rik	RIKEN cDNA 4930579C15 gene	[2]
4930579J09Rik	RIKEN cDNA 4930579J09 gene	[2]
4931408C20Rik	RIKEN cDNA 4931408C20 gene	[2]
4931423N10Rik	RIKEN cDNA 4931423N10 gene	[2]

Proteins from previous mouse sperm proteomes		
**Protein Symbol**	**Protein Name**	**Reference**
4931432M23Rik	RIKEN cDNA 4931432M23 gene	[2]
4932425I24Rik	RIKEN cDNA 4932425I24 gene	[2]
4932441B19Rik	RIKEN cDNA 4932441B19 gene	[2]
4933400C05Rik	RIKEN cDNA 4933400C05 gene	[2]
4933405O20Rik	RIKEN cDNA 4933405O20 gene	[2]
4933411K16Rik	RIKEN cDNA 4933411K16 gene	[2]
5730437N04Rik	RIKEN cDNA 5730437N04 gene	[2]
5730469M10Rik	RIKEN cDNA 5730469M10 gene	[2]
9030607L17Rik	RIKEN cDNA 9030607L17 gene	[2]
9130227C08Rik	RIKEN cDNA 9130227C08Rik gene	[2]
9230002F21Rik	defensin beta 22	[1]
AA467197	expressed sequence AA467197	[2]
Abca13	ATP-binding cassette, sub-family A (ABC1), member 13	[2]
Abcb10	ATP-binding cassette, sub-family B (MDR/TAP), member 10	[2]
Abcc12	ATP-binding cassette, sub-family C (CFTR/MRP), member 12	[1]
Abcg2	ATP-binding cassette, sub-family G (WHITE), member 2	[1],[3]
Abhd11	abhydrolase domain containing 11	[2]
Acad9	acyl-Coenzyme A dehydrogenase family, member 9	[2]
Acadvl	acyl-Coenzyme A dehydrogenase, very long chain	[2]
Acat1	acetyl-Coenzyme A acetyltransferase 1	[2]
Ace	angiotensin I converting enzyme (peptidyl-dipeptidase A) 1	[1],[2]
Ace3	angiotensin I converting enzyme (peptidyl-dipeptidase A) 3	[1],[2]
Aco2	aconitase 2, mitochondrial	[2]
Acot10	acyl-CoA thioesterase 10	[2]
Acot7	acyl-CoA thioesterase 7	[2]
Acot9	acyl-CoA thioesterase 9	[2]
Acr	acrosin prepropeptide	[1],[2]
Acrbp	proacrosin binding protein	[1],[2]
Acrv1	acrosomal vesicle protein 1	[1],[2]
Acsbg2	acyl-CoA synthetase bubblegum family member 2	[2]
Acsl1	acyl-CoA synthetase long-chain family member 1	[2],[3]
Acta1	actin, alpha 1, skeletal muscle	[2]
Actb	actin, beta	[2],[3]
Actc1	actin, alpha, cardiac muscle 1	[3]

Proteins from previous mouse sperm proteomes		
**Protein Symbol**	**Protein Name**	**Reference**
Actl7a	actin-like 7a	[2]
Actn4	actinin alpha 4	[3]
Actrt1	actin-related protein T1	[2]
Actrt2	actin-related protein T2	[2]
Acyp1	acylphosphatase 1, erythrocyte (common) type	[2]
Adam1b	a disintegrin and metallopeptidase domain 1b	[1],[2]
Adam2	a disintegrin and metallopeptidase domain 2	[1],[2]
Adam24	a disintegrin and metallopeptidase domain 24 (testase 1)	[1]
Adam3	a disintegrin and metallopeptidase domain 3 (cyritestin)	[1],[2]
Adam4	a disintegrin and metallopeptidase domain 4	[1]
Adam5	a disintegrin and metallopeptidase domain 5	[1]
Adam6	a disintegrin and metallopeptidase domain 6A	[1],[2]
Adh1	alcohol dehydrogenase 1 (class I)	[3]
Agfg1	ArfGAP with FG repeats 1	[2]
Agt	angiotensinogen (serpin peptidase inhibitor, clade A, member 8)	[3]
Ahcy	S-adenosylhomocysteine hydrolase	[2]
Aim1l	absent in melanoma 1-like	[2]
Ak1	adenylate kinase 1	[2]
Ak2	adenylate kinase 2	[2]
Ak7	adenylate kinase 7	[2]
Akap13	A kinase (PRKA) anchor protein 13	[2]
Akap3	A kinase (PRKA) anchor protein 3	[2],[3]
Akap4	A kinase (PRKA) anchor protein 4	[2],[3]
Akr1a4	aldo-keto reductase family 1, member A4 (aldehyde reductase)	[3]
Akr1b10	aldo-keto reductase family 1, member B10 (aldose reductase)	[2]
Akr1b3	aldo-keto reductase family 1, member B3 (aldose reductase)	[2],[3]
Akr1b7	aldo-keto reductase family 1, member B7	[3]
Akr7a5	aldo-keto reductase family 7, member A5 (aflatoxin aldehyde reductase)	[2]
Alad	aminolevulinate, delta-, dehydratase	[3]
Alb	albumin	[2],[3]
Aldh1a1	aldehyde dehydrogenase family 1, subfamily A1	[3]
Aldh1a2	aldehyde dehydrogenase family 1, subfamily A2	[2]
Aldh9a1	aldehyde dehydrogenase 9, subfamily A1	[2]

Proteins from previous mouse sperm proteomes		
**Protein Symbol**	**Protein Name**	**Reference**
Aldoa	aldolase A, fructose-bisphosphate	[2],[3]
Aldoart2	aldolase 1, A isoform, retrogene 2	[3]
Aldoc	aldolase C, fructose-bisphosphate	[2]
Alms1	Alstrom syndrome 1 homolog (human)	[2]
Als2cr11	amyotrophic lateral sclerosis 2 (juvenile) chromosome region, candidate 11 (human)	[2]
Amdhd2	amidohydrolase domain containing 2	[2]
Anapc5	anaphase-promoting complex subunit 5	[2]
Ank1	ankyrin 1, erythroid	[2]
Ankrd11	ankyrin repeat domain 11	[2]
Ankrd5	ankyrin repeat domain 5	[2]
Ankrd57	ankyrin repeat domain 57	[2]
Anxa2	annexin A2	[3]
Anxa4	annexin A4	[3]
Anxa5	annexin A5	[3]
Anxa6	annexin A6	[3]
Ap3d1	adaptor-related protein complex 3, delta 1 subunit	[2]
Apbb2	amyloid beta (A4) precursor protein-binding, family B, member 2	[2]
Apoa1	apolipoprotein A-I	[3]
Apoa1bp	apolipoprotein A-I binding protein	[1],[2]
Apoa2	apolipoprotein A-II	[3]
Apoa4	apolipoprotein A-IV	[3]
Apof	apolipoprotein F	[2]
Apoo	apolipoprotein O	[2]
Apool	apolipoprotein O-like	[2]
Aqp7	aquaporin 7	[1],[2]
Arf2	ADP-ribosylation factor 2	[2]
Arf6	ADP-ribosylation factor 6	[2]
Arhgap17	Rho GTPase activating protein 17	[2]
Arhgdia	Rho GDP dissociation inhibitor (GDI) alpha	[3]
Arhgef1	Rho guanine nucleotide exchange factor (GEF) 1	[2]
Armc3	armadillo repeat containing 3	[2]
Arpm1	actin related protein M1	[2]
Arsa	arylsulfatase A	[1],[2]
Art3	ADP-ribosyltransferase 3	[2]

Proteins from previous mouse sperm proteomes		
**Protein Symbol**	**Protein Name**	**Reference**
Asb9	ankyrin repeat and SOCS box-containing 9	[2]
Asrgl1	asparaginase like 1	[2]
Atg16l1	autophagy-related 16-like 1 (yeast)	[2]
Atn1	atrophin 1	[2]
Atox1	ATX1 (antioxidant protein 1) homolog 1 (yeast)	[3]
Atp13a1	ATPase type 13A1	[2]
Atp1a1	ATPase, Na+/K+ transporting, alpha 1 polypeptide	[1]
Atp1a2	ATPase, Na+/K+ transporting, alpha 2 polypeptide	[2]
Atp1a3	ATPase, Na+/K+ transporting, alpha 3 polypeptide	[2]
Atp1a4	ATPase, Na+/K+ transporting, alpha 4 polypeptide	[1]
Atp1b3	ATPase, Na+/K+ transporting, beta 3 polypeptide	[1],[2]
Atp2b2	ATPase, Ca++ transporting, plasma membrane 2	[2]
Atp2b4	ATPase, Ca++ transporting, plasma membrane 4	[1],[2]
Atp5a1	ATP synthase, H+ transporting, mitochondrial F1 complex, alpha subunit, isoform 1	[2],[3]
Atp5b	ATP synthase, H+ transporting mitochondrial F1 complex, beta subunit	[2],[3]
Atp5c1	ATP synthase, H+ transporting, mitochondrial F1 complex, gamma polypeptide 1	[2]
Atp5d	ATP synthase, H+ transporting, mitochondrial F1 complex, delta subunit	[2]
Atp5f1	ATP synthase, H+ transporting, mitochondrial F0 complex, subunit b, isoform 1	[2]
Atp5h	ATP synthase, H+ transporting, mitochondrial F0 complex, subunit d	[2]
Atp5k	ATP synthase, H+ transporting, mitochondrial F1F0 complex, subunit e	[2]
Atp5l	ATP synthase, H+ transporting, mitochondrial F0 complex, subunit g	[2]
Atp5o	ATP synthase, H+ transporting, mitochondrial F1 complex, O subunit	[2]
Atp5s	ATP synthase, H+ transporting, mitochondrial F0 complex, subunit s	[2]
Atp6v1a	ATPase, H+ transporting, lysosomal V1 subunit A	[2]
Atp6v1b2	ATPase, H+ transporting, lysosomal V1 subunit B2	[2]
Atp6v1e1	ATPase, H+ transporting, lysosomal V1 subunit E1	[2]
Atp6v1h	ATPase, H+ transporting, lysosomal V1 subunit H	[2]
Atp8b3	ATPase, class I, type 8B, member 3	[1]
B2m	beta-2 microglobulin	[3]
B4galt4	UDP-Gal:betaGlcNAc beta 1,4-galactosyltransferase, polypeptide 4	[2]
Basp1	brain abundant, membrane attached signal protein 1	[2],[3]
Bat3	HLA-B-associated transcript 3	[2]
BB014433	expressed sequence BB014433	[2]

Proteins from previous mouse sperm proteomes		
**Protein Symbol**	**Protein Name**	**Reference**
BC005764	cDNA sequence BC005764	[2]
BC049635	cDNA sequence BC049635	[2]
BC051142	cDNA sequence BC051142	[2]
BC089491	cDNA sequence BC089491	[2]
Bcs1l	BCS1-like (yeast)	[2]
Bdh1	3-hydroxybutyrate dehydrogenase, type 1	[2]
Birc6	baculoviral IAP repeat-containing 6	[2]
Blvra	biliverdin reductase A	[2]
Bpgm	2,3-bisphosphoglycerate mutase	[2]
Brp44	brain protein 44	[2]
Bsg	basigin	[1],[2],[3]
Bspry	B-box and SPRY domain containing	[2]
Bzrap1	benzodiazapine receptor associated protein 1	[2]
C130030J05		[2]
C130090K23Rik	cell wall biogenesis 43 C-terminal homolog (S. cerevisiae)	[1]
C1qbp	complement component 1, q subcomponent binding protein	[2]
C3	complement component 3	[3]
C77370	expressed sequence C77370	[2]
C920008N22Rik	RIKEN cDNA C920008N22 gene	[2]
Cabyr	calcium-binding tyrosine-(Y)-phosphorylation regulated (fibrousheathin 2)	[2]
Cacng3	calcium channel, voltage-dependent, gamma subunit 3	[2]
Calm1	calmodulin 1	[2]
Calm2	calmodulin 2	[3]
Calm3	calmodulin 3	[3]
Calr	calreticulin	[1],[3]
Camk4	calcium/calmodulin-dependent protein kinase IV	[2]
Cand1	cullin associated and neddylation disassociated 1	[2]
Canx	calnexin	[1],[2],[3]
Cap1	CAP, adenylate cyclase-associated protein 1 (yeast)	[3]
Capza3	capping protein (actin filament) muscle Z-line, alpha 3	[2]
Capzb	capping protein (actin filament) muscle Z-line, beta	[2]
Car2	carbonic anhydrase 2	[2]
Car3	carbonic anhydrase 3	[3]
Car4	carbonic anhydrase 4	[1],[2]

Proteins from previous mouse sperm proteomes		
**Protein Symbol**	**Protein Name**	**Reference**
Casc1	cancer susceptibility candidate 1	[2]
Catsper4	cation channel, sperm associated 4	[2]
Cbr1	carbonyl reductase 1	[3]
Cbr4	carbonyl reductase 4	[2]
Ccdc105	coiled-coil domain containing 105	[2]
Ccdc11	coiled-coil domain containing 11	[2]
Ccdc116	coiled-coil domain containing 116	[2]
Ccdc135	coiled-coil domain containing 135	[2]
Ccdc136	coiled-coil domain containing 136	[2]
Ccdc151	coiled-coil domain containing 151	[2]
Ccdc19	coiled-coil domain containing 19	[2]
Ccdc21	coiled-coil domain containing 21	[2]
Ccdc27	coiled-coil domain containing 27	[2]
Ccdc40	coiled-coil domain containing 40	[2]
Ccdc58	coiled-coil domain containing 58	[2]
Ccdc63	coiled-coil domain containing 63	[2]
Ccdc81	coiled-coil domain containing 81	[2]
Ccdc88c	coiled-coil domain containing 88C	[2]
Ccin	calicin	[2]
Cct2	chaperonin containing Tcp1, subunit 2 (beta)	[2]
Cct3	chaperonin containing Tcp1, subunit 3 (gamma)	[2]
Cct4	chaperonin containing Tcp1, subunit 4 (delta)	[2]
Cct5	chaperonin containing Tcp1, subunit 5 (epsilon)	[2]
Cct6a	chaperonin containing Tcp1, subunit 6a (zeta)	[2]
Cct7	chaperonin containing Tcp1, subunit 7 (eta)	[2]
Cct8	chaperonin containing Tcp1, subunit 8 (theta)	[2]
Cd109	CD109 antigen	[1],[2]
Cd46	CD46 antigen, complement regulatory protein	[1],[2]
Cd55	CD55 antigen	[2]
Cdc34	cell division cycle 34 homolog (S. cerevisiae)	[2]
Ceacam2	carcinoembryonic antigen-related cell adhesion molecule 2	[2]
Cecr5	cat eye syndrome chromosome region, candidate 5 homolog (human)	[2]
Cenpe	centromere protein E	[2]
Cep152	centrosomal protein 152	[2]

Proteins from previous mouse sperm proteomes		
**Protein Symbol**	**Protein Name**	**Reference**
Cep350	centrosomal protein 350	[2]
Ces3	carboxylesterase 3	[1],[3]
Ces7	carboxylesterase 7	[1],[3]
Cfh	complement component factor h	[2]
Cfl1	cofilin 1, non-muscle	[3]
Chchd6	coiled-coil-helix-coiled-coil-helix domain containing 6	[2]
Chdh	choline dehydrogenase	[2]
Chit1	chitinase 1 (chitotriosidase)	[1]
Cisd1	CDGSH iron sulfur domain 1	[2]
Ckb	creatine kinase, brain	[3]
Ckm	creatine kinase, muscle	[3]
Clca1	chloride channel calcium activated 1	[1]
Clca2	chloride channel calcium activated 2	[1]
Clic1	chloride intracellular channel 1	[3]
Clmn	calmin	[2]
Cltc	clathrin, heavy polypeptide (Hc)	[2]
Clu	clusterin	[1],[2],[3]
Cmpk1	cytidine monophosphate (UMP-CMP) kinase 1	[2]
Cnn1	calponin 1	[3]
Coil	coilin	[2]
Col8a2	collagen, type VIII, alpha 2	[2]
Commd10	COMM domain containing 10	[2]
Copb1	coatomer protein complex, subunit beta 1	[3]
Cotl1	coactosin-like 1 (Dictyostelium)	[3]
Cox4i1	cytochrome c oxidase subunit IV isoform 1	[2]
Cox5a	cytochrome c oxidase, subunit Va	[2]
Cox6b2	cytochrome c oxidase subunit VIb polypeptide 2	[2]
Cox6c	cytochrome c oxidase, subunit VIc	[2]
Cox7a2	cytochrome c oxidase, subunit VIIa 2	[2]
Cox7a2l	cytochrome c oxidase subunit VIIa polypeptide 2-like	[2]
Cp	ceruloplasmin	[3]
Cpa5	carboxypeptidase A5	[1],[2]
Cpt1b	carnitine palmitoyltransferase 1b, muscle	[2]
Cpt2	carnitine palmitoyltransferase 2	[2]

Proteins from previous mouse sperm proteomes		
**Protein Symbol**	**Protein Name**	**Reference**
Cpvl	carboxypeptidase, vitellogenic-like	[1]
Crat	carnitine acetyltransferase	[2]
Crisp1	cysteine-rich secretory protein 1	[2],[3]
Cryl1	crystallin, lambda 1	[2]
Cryzl1	crystallin, zeta (quinone reductase)-like 1	[2]
Cs	citrate synthase	[2],[3]
Csl	citrate synthase like	[2],[3]
Csnk1a1	casein kinase 1, alpha 1	[2]
Csnk2a2	casein kinase 2, alpha prime polypeptide	[2]
Csnk2b	casein kinase 2, beta polypeptide	[2]
Csrp1	cysteine and glycine-rich protein 1	[3]
Cubn	cubilin (intrinsic factor-cobalamin receptor)	[2]
Cul3	cullin 3	[2]
Cutc	cutC copper transporter homolog (E.coli)	[2]
Cuzd1	CUB and zona pellucida-like domains 1	[1],[2]
Cwf19l1	CWF19-like 1, cell cycle control (S. pombe)	[2]
Cyb5	cytochrome b-5	[3]
Cyb5b	cytochrome b5 type B	[2]
Cyc1	cytochrome c-1	[2]
Cycs	cytochrome c, somatic	[2]
Cyct	cytochrome c, testis	[2]
Cylc1	cylicin, basic protein of sperm head cytoskeleton 1	[2]
Cylc2	cylicin, basic protein of sperm head cytoskeleton 2	[2]
D10Jhu81e	DNA segment, Chr 10, Johns Hopkins University 81 expressed	[2]
D11Wsu47e	DNA segment, Chr 11, Wayne State University 47, expressed	[2]
D230040A04Rik	RIKEN cDNA D230040A04 gene	[2]
Dak	dihydroxyacetone kinase 2 homolog (yeast)	[2]
Dbil5	diazepam binding inhibitor-like 5	[3]
Ddt	D-dopachrome tautomerase	[2],[3]
Dhrs1	dehydrogenase/reductase (SDR family) member 1	[2]
Dhrs4	dehydrogenase/reductase (SDR family) member 4	[2]
Dhrs7b	dehydrogenase/reductase (SDR family) member 7B	[2]
Dhx29	DEAH (Asp-Glu-Ala-His) box polypeptide 29	[2]
Disc1	disrupted in schizophrenia 1	[2]

Proteins from previous mouse sperm proteomes		
**Protein Symbol**	**Protein Name**	**Reference**
Dlat	dihydrolipoamide S-acetyltransferase (E2 component of pyruvate dehydrogenase complex)	[2],[3]
Dld	dihydrolipoamide dehydrogenase	[2]
Dlst	dihydrolipoamide S-succinyltransferase (E2 component of 2-oxo-glutarate complex)	[2]
Dmc1	DMC1 dosage suppressor of mck1 homolog, meiosis-specific homologous recombination (yeast)	[2]
Dmxl2	Dmx-like 2	[2]
Dnahc1	dynein, axonemal, heavy chain 1	[2]
Dnahc10	dynein, axonemal, heavy chain 10	[2]
Dnahc17	dynein, axonemal, heavy chain 17	[2]
Dnahc2	dynein, axonemal, heavy chain 2	[2]
Dnahc3	dynein, axonemal, heavy chain 3	[2]
Dnahc5	dynein, axonemal, heavy chain 5	[2]
Dnahc6	dynein, axonemal, heavy chain 6	[2]
Dnahc7a	dynein, axonemal, heavy chain 7A	[2]
Dnahc7b	dynein, axonemal, heavy chain 7B	[2]
Dnahc7l	dynein, axonemal, heavy chain 12	[2]
Dnahc8	dynein, axonemal, heavy chain 8	[2]
Dnahc9	dynein, axonemal, heavy chain 9	[2]
Dnaic1	dynein, axonemal, intermediate chain 1	[2]
Dnaic2	dynein, axonemal, intermediate chain 2	[2]
Dnaja4	DnaJ (Hsp40) homolog, subfamily A, member 4	[2]
Dnajb11	DnaJ (Hsp40) homolog, subfamily B, member 11	[2]
Dnajb13	DnaJ (Hsp40) related, subfamily B, member 13	[2]
Dnajb3	DnaJ (Hsp40) homolog, subfamily B, member 3	[2]
Dnajb6	DnaJ (Hsp40) homolog, subfamily B, member 6	[2]
Dnajc11	DnaJ (Hsp40) homolog, subfamily C, member 11	[2]
Dnali1	dynein, axonemal, light intermediate polypeptide 1	[2]
Dnpep	aspartyl aminopeptidase	[2],[3]
Dock11	dedicator of cytokinesis 11	[2]
Dpep3	dipeptidase 3	[1],[2],[3]
Dpp3	dipeptidylpeptidase 3	[2]
Drd2	dopamine receptor 2	[2]
Dsg1c	desmoglein 1 gamma	[2]
Dstn	destrin	[3]
Dynll1	dynein light chain LC8-type 1	[2]

Proteins from previous mouse sperm proteomes		
**Protein Symbol**	**Protein Name**	**Reference**
Dynlt1	dynein light chain Tctex-type 1D	[2]
E130112L23Rik	RIKEN cDNA E130112L23 gene	[2]
E430028B21Rik	phosphodiesterase 12	[2]
Eef1a1	eukaryotic translation elongation factor 1 alpha 1	[2],[3]
Eef1d	eukaryotic translation elongation factor 1 delta (guanine nucleotide exchange protein)	[3]
Eef1g	eukaryotic translation elongation factor 1 gamma	[2]
Efcab3	EF-hand calcium binding domain 3	[2]
Efhb	EF hand domain family, member B	[2]
Efhc1	EF-hand domain (C-terminal) containing 1	[2]
Efhc2	EF-hand domain (C-terminal) containing 2	[2]
EG433182	predicted gene 5506	[3]
Eif2ak4	eukaryotic translation initiation factor 2 alpha kinase 4	[2]
Elmo3	engulfment and cell motility 3, ced-12 homolog (C. elegans)	[2]
Enah	enabled homolog (Drosophila)	[2]
Enkur	enkurin, TRPC channel interacting protein	[2]
Eno1	enolase 1, alpha non-neuron	[2],[3]
Eno2	enolase 2, gamma neuronal	[2]
Eno3	enolase 3, beta muscle	[2]
Enpp5	ectonucleotide pyrophosphatase/phosphodiesterase 5	[1]
Eprs	glutamyl-prolyl-tRNA synthetase	[2]
Ercc6	excision repair cross-complementing rodent repair deficiency, complementation group 6	[2]
Ergic1	endoplasmic reticulum-golgi intermediate compartment (ERGIC) 1	[3]
Erlin2	ER lipid raft associated 2	[2]
Es1	esterase 1	[3]
Etfa	electron transferring flavoprotein, alpha polypeptide	[2]
Etfdh	electron transferring flavoprotein, dehydrogenase	[2]
Fabp4	fatty acid binding protein 4, adipocyte	[3]
Fabp9	fatty acid binding protein 9, testis	[2],[3]
Fahd2a	fumarylacetoacetate hydrolase domain containing 2A	[2]
Fam154a	family with sequence similarity 154, member A	[2]
Fam166a	family with sequence similarity 166, member A	[2]
Fam170b	family with sequence similarity 170, member B	[2]
Fam178a	family with sequence similarity 178, member A	[2]
Fam71a	family with sequence similarity 71, member A	[2]

Proteins from previous mouse sperm proteomes		
**Protein Symbol**	**Protein Name**	**Reference**
Fam71b	family with sequence similarity 71, member B	[2],[3]
Fam78a	family with sequence similarity 78, member A	[2]
Fank1	fibronectin type 3 and ankyrin repeat domains 1	[2]
Fbn1	fibrillin 1	[2]
Fbp1	fructose bisphosphatase 1	[2]
Fdx1l	ferredoxin 1-like	[2]
Fgd1	FYVE, RhoGEF and PH domain containing 1	[3]
Fh1	fumarate hydratase 1	[2]
fh15		[2]
Fhl4	four and a half LIM domains 4	[2]
Fis1	fission 1 (mitochondrial outer membrane) homolog (yeast)	[2]
Fn1	fibronectin 1	[1]
Fndc3a	fibronectin type III domain containing 3A	[2]
Fndc8	fibronectin type III domain containing 8	[2]
Foxo3	forkhead box O3	[2]
Frmd4a	FERM domain containing 4A	[2]
Fscb	fibrous sheath CABYR binding protein	[2]
Fscn3	fascin homolog 3, actin-bundling protein, testicular (Strongylocentrotus purpuratus)	[2]
Fsip2	fibrous sheath-interacting protein 2	[2]
Ftmt	ferritin mitochondrial	[2]
Fxn	frataxin	[2]
G6pd2	glucose-6-phosphate dehydrogenase 2	[2]
Ganab	alpha glucosidase 2 alpha neutral subunit	[1]
Gapdh	glyceraldehyde-3-phosphate dehydrogenase	[3]
Gapdhs	glyceraldehyde-3-phosphate dehydrogenase, spermatogenic	[3]
Gas8	growth arrest specific 8	[2]
Gc	group specific component	[3]
Gcc2	GRIP and coiled-coil domain containing 2	[2]
Gdi2	guanosine diphosphate (GDP) dissociation inhibitor 2	[3]
Gfap	glial fibrillary acidic protein	[2]
Gk2	glycerol kinase 2	[2]
Glb1l	galactosidase, beta 1-like	[1]
Glcci1	glucocorticoid induced transcript 1	[2]
glg1	golgi apparatus protein 1	[2]

Proteins from previous mouse sperm proteomes		
**Protein Symbol**	**Protein Name**	**Reference**
Glipr1l1	GLI pathogenesis-related 1 like 1	[2]
Glipr1l2	GLI pathogenesis-related 1 like 2	[2]
Glud1	glutamate dehydrogenase 1	[2]
Glul	glutamate-ammonia ligase (glutamine synthetase)	[2]
Gm128	predicted gene 128	[2]
Gm1281		[2]
Gm13334	predicted gene 13334	[2]
Gm136	predicted gene 136	[2]
Gm166	predicted gene 166	[2]
Gm1673	predicted gene 1673	[2]
Gm3336	predicted gene 3336	[2]
Gm4535	predicted gene 4535	[2]
Gm46	predicted gene 46	[1],[2]
Gm4764	predicted gene 4764	[2]
Gm595	predicted gene 595	[2]
Gm6316	predicted gene 6316	[2]
Gm6413	predicted gene 6413	[2]
Gm6981	predicted gene 6981	[2]
Gm8394	predicted gene 8394	[2]
Gm884	predicted gene 884	[2]
Gm9047	predicted gene 9047	[2]
Gm9832	predicted gene 9832	[2]
Gnpda1	glucosamine-6-phosphate deaminase 1	[2]
Gnpda2	glucosamine-6-phosphate deaminase 2	[2]
Gpd1	glycerol-3-phosphate dehydrogenase 1 (soluble)	[2]
gpd2	glycerol phosphate dehydrogenase 2, mitochondrial	[2]
Gpi1	glucose phosphate isomerase 1	[2],[3]
Gpx3	glutathione peroxidase 3	[1],[2]
Gpx4	glutathione peroxidase 4	[2],[3]
Gpx5	glutathione peroxidase 5	[1],[3]
Gsn	gelsolin	[3]
Gstm1	glutathione S-transferase, mu 1	[3]
Gstm2	glutathione S-transferase, mu 2	[3]
Gstm5	glutathione S-transferase, mu 5	[2],[3]

Proteins from previous mouse sperm proteomes		
**Protein Symbol**	**Protein Name**	**Reference**
Gsto1	glutathione S-transferase omega 1	[2]
Gsto2	glutathione S-transferase omega 2	[2]
Gstp1	glycerol kinase-like 1	[3]
Gstt2	glutathione S-transferase, theta 2	[2]
Gstt3	glutathione S-transferase, theta 3	[2]
Gtl3	gene trap locus 3	[2]
Gyk	glycerol kinase	[2]
Gykl1	glycerol kinase-like 1	[2],[3]
H2-Ke6	H2-K region expressed gene 6	[2]
Hadha	hydroxyacyl-Coenzyme A dehydrogenase/3-ketoacyl-Coenzyme A thiolase/enoyl-Coenzyme A hydratase (trifunctional protein), alpha subunit	[2]
Hadhb	hydroxyacyl-Coenzyme A dehydrogenase/3-ketoacyl-Coenzyme A thiolase/enoyl-Coenzyme A hydratase (trifunctional protein), beta subunit	[2]
Hba-a1	hemoglobin alpha, adult chain 1	[2],[3]
Hba-a2	hemoglobin, beta adult major chain	[3]
Hbb-b1	hemoglobin, beta adult major chain	[2],[3]
Hbb-b2	hexokinase 1	[3]
Hc	hemolytic complement	[2]
Hdac1	histone deacetylase 1	[2]
Hdhd1a	haloacid dehalogenase-like hydrolase domain containing 1A	[2]
Hdhd3	haloacid dehalogenase-like hydrolase domain containing 3	[2]
Heatr7a	HEAT repeat containing 7A	[2]
Heatr7b2	XVHEAT repeat family member 7B2	[2]
Hibadh	3-hydroxyisobutyrate dehydrogenase	[2]
Hibch	3-hydroxyisobutyryl-Coenzyme A hydrolase	[2]
Hint1	histidine triad nucleotide binding protein 1	[2]
Hira	histone cell cycle regulation defective homolog A (S. cerevisiae)	[2]
Hist1h1t	histone cluster 1, H1t	[2]
Hist1h2ba	histone cluster 1, H2ba	[2]
Hist1h4i	histone cluster 1, H4i	[2]
Hk1	hexokinase 1	[2],[3]
Hk2	hexokinase 2	[2]
Hkdc1	hexokinase domain containing 1	[2]
Hmox2	heme oxygenase (decycling) 2	[2]

Proteins from previous mouse sperm proteomes		
**Protein Symbol**	**Protein Name**	**Reference**
Hnrnpu	heterogeneous nuclear ribonucleoprotein U	[2]
Hnrpa2b1	hypoxanthine guanine phosphoribosyl transferase 1	[3]
Hprt1	hemopexin	[3]
Hpx	heat shock protein 90 alpha (cytosolic), class B member 1	[3]
Hsd17b10	hydroxysteroid (17-beta) dehydrogenase 10	[2]
Hsp90aa1	heat shock protein 90, alpha (cytosolic), class A member 1	[2]
Hsp90ab1	heat shock protein 90 alpha (cytosolic), class B member 1	[2],[3]
Hsp90b1	heat shock protein 90, beta (Grp94), member 1	[1],[2]
Hspa1b	heat shock protein 5	[3]
Hspa1l	heat shock protein 1-like	[2]
Hspa2	heat shock protein 2	[2]
Hspa4l	heat shock protein 4 like	[2]
Hspa5	heat shock protein 5	[1],[2],[3]
Hspa8	heat shock protein 8	[2],[3]
Hspa9	heat shock protein 9	[2]
Hspb9	heat shock protein, alpha-crystallin-related, B9	[2]
Hspbp1	HSPA (heat shock 70kDa) binding protein, cytoplasmic cochaperone 1	[2]
Hspd1	heat shock protein 1 (chaperonin)	[2]
Htra2	HtrA serine peptidase 2	[2]
Htt	huntingtin	[2]
Hyal5	hyaluronoglucosaminidase 5	[1],[2]
Hydin	hydrocephalus inducing	[2]
Hyou1	hypoxia up-regulated 1	[1]
Idh1	isocitrate dehydrogenase 1 (NADP+), soluble	[2],[3]
Idh3a	isocitrate dehydrogenase 3 (NAD+) alpha	[2]
Idh3b	isocitrate dehydrogenase 3 (NAD+) beta	[2]
Igbp1b	immunoglobulin (CD79A) binding protein 1b	[2]
Ighg1	keratin 1	[3]
Il4i1	interleukin 4 induced 1	[1]
Immt	inner membrane protein, mitochondrial	[2]
Impa1	inositol (myo)-1(or 4)-monophosphatase 1	[2]
Ing4	inhibitor of growth family, member 4	[2]
Inpp5a	inositol polyphosphate-5-phosphatase A	[2]
Iqcd	IQ motif containing D	[2]

Proteins from previous mouse sperm proteomes		
**Protein Symbol**	**Protein Name**	**Reference**
Iqcf5	IQ motif containing F5	[2]
Irgc1	immunity-related GTPase family, cinema 1	[2]
Isoc2a	isochorismatase domain containing 2a	[2]
Isyna1	myo-inositol 1-phosphate synthase A1	[2]
Itga5	integrin alpha 5 (fibronectin receptor alpha)	[1]
Itgb2	integrin beta 2	[1]
Itpr2	inositol 1,4,5-triphosphate receptor 2	[2]
Izumo1	izumo sperm-egg fusion 1	[1],[2]
Kcnk4	potassium channel, subfamily K, member 4	[2]
Kif24	kinesin family member 24	[2]
Kif9	kinesin family member 9	[2]
Klhl10	kelch-like 10 (Drosophila)	[2]
Kndc1	kinase non-catalytic C-lobe domain (KIND) containing 1	[2]
Krt1	keratin 1	[2],[3]
Krt10	keratin 10	[2]
Krt19	keratin 19	[2]
Krt2	keratin 2	[2]
Krt24	keratin 24	[2]
Krt79	keratin 79	[2],[3]
Krt8	keratin 8	[3]
Krt81	keratin 81	[2]
Krt85	keratin 85	[2]
L2hgdh	L-2-hydroxyglutarate dehydrogenase	[2]
Lamb1-1	laminin B1 subunit 1	[2]
Lamb2	laminin, beta 2	[2]
Lap3	leucine aminopeptidase 3	[2]
Lcn5	lipocalin 5	[2],[3]
Ldha	lactate dehydrogenase A-like 6B	[3]
Ldhal6b	lactate dehydrogenase C	[3]
Ldhb	lactate dehydrogenase B	[2]
Ldhc	lactate dehydrogenase C	[2],[3]
Letm1	leucine zipper-EF-hand containing transmembrane protein 1	[2]
Lipe	lipase, hormone sensitive	[2]
Lman2	lectin, mannose-binding 2	[1]

Proteins from previous mouse sperm proteomes		
**Protein Symbol**	**Protein Name**	**Reference**
Lpp	LIM domain containing preferred translocation partner in lipoma	[2]
Lrch3	leucine-rich repeats and calponin homology (CH) domain containing 3	[2]
Lrguk	leucine-rich repeats and guanylate kinase domain containing	[2]
Lrrc18	leucine rich repeat containing 18	[2]
Lrrc24	leucine rich repeat containing 24	[2]
Lrrc34	leucine rich repeat containing 34	[2]
Lrrc37a	leucine rich repeat containing 37A	[1]
Lrrc57	leucine rich repeat containing 57	[2]
Lrrc7	leucine rich repeat containing 7	[2]
Lta4h	leukotriene A4 hydrolase	[2]
Ltb4dh	leukotriene B4 dehydrogenase	[3]
Ltf	lactotransferrin	[3]
Lum	lumican	[3]
Luzp1	leucine zipper protein 1	[2]
Ly6g5b	lymphocyte antigen 6 complex, locus G5B	[2]
Lypd4	Ly6/Plaur domain containing 4	[2]
Lypla1	lysophospholipase 1	[2]
Lyzl1	lysozyme-like 1	[2]
Lyzl6	lysozyme-like 6	[1]
M6pr	mannose-6-phosphate receptor, cation dependent	[1]
Macf1	microtubule-actin crosslinking factor 1	[2]
Man2b2	mannosidase 2, alpha B2	[1],[2]
Man2c1	mannosidase, alpha, class 2C, member 1	[1],[2]
Mdh1	mucin 5, subtype B, tracheobronchial	[3]
Mdh2	malate dehydrogenase 2, NAD (mitochondrial)	[2]
Mdn1	midasin homolog (yeast)	[2]
Me1	malic enzyme 1, NADP(+)-dependent, cytosolic	[2]
Me2	malic enzyme 2, NAD(+)-dependent, mitochondrial	[2]
Mecr	mitochondrial trans-2-enoyl-CoA reductase	[2]
Mfge8	milk fat globule-EGF factor 8 protein	[1]
Mink1	misshapen-like kinase 1 (zebrafish)	[2]
Mipol1	mirror-image polydactyly gene 1 homolog (human)	[2]
Mll2	myeloid/lymphoid or mixed-lineage leukemia 2	[2]
Mmel1	membrane metallo-endopeptidase-like 1	[1]

Proteins from previous mouse sperm proteomes		
**Protein Symbol**	**Protein Name**	**Reference**
Mns1	meiosis-specific nuclear structural protein 1	[2]
Mpi	mannose phosphate isomerase	[2]
Mrps36	mitochondrial ribosomal protein S36	[2]
Ms4a14	membrane-spanning 4-domains, subfamily A, member 14	[2]
Mtap1a	microtubule-associated protein 1 A	[2]
Mtch2	mitochondrial carrier homolog 2 (C. elegans)	[2]
Mtx2	metaxin 2	[2]
Muc5	murinoglobulin 1	[3]
Mug1	myosin, heavy polypeptide 10, non-muscle	[3]
Mybl2	myeloblastosis oncogene-like 2	[2]
Myh10	myosin, heavy polypeptide 11, smooth muscle	[3]
Myh11	myosin, heavy polypeptide 9, non-muscle	[3]
Myh9	myosin, light polypeptide 6, alkali, smooth muscle and non-muscle	[3]
Myl6	myosin, light polypeptide 9, regulatory	[3]
Myl9	myosin, light chain 12B, regulatory	[3]
Mylc2b	myosin, light polypeptide kinase	[3]
Mylk	myosin IB	[3]
Myo1b	family with sequence similarity 129, member A	[3]
Nagk	N-acetylglucosamine kinase	[2]
Naglu	alpha-N-acetylglucosaminidase (Sanfilippo disease IIIB)	[1],[2]
Ndufa10	NADH dehydrogenase (ubiquinone) 1 alpha subcomplex 10	[2]
Ndufa11	NADH dehydrogenase (ubiquinone) 1 alpha subcomplex 11	[2]
Ndufa12	NADH dehydrogenase (ubiquinone) 1 alpha subcomplex, 12	[2]
Ndufa13	NADH dehydrogenase (ubiquinone) 1 alpha subcomplex, 13	[2]
Ndufa2	NADH dehydrogenase (ubiquinone) 1 alpha subcomplex, 2	[2]
Ndufa5	NADH dehydrogenase (ubiquinone) 1 alpha subcomplex, 5	[2]
Ndufa6	NADH dehydrogenase (ubiquinone) 1 alpha subcomplex, 6 (B14)	[2]
Ndufa7	NADH dehydrogenase (ubiquinone) 1 alpha subcomplex, 7 (B14.5a)	[2]
Ndufa8	NADH dehydrogenase (ubiquinone) 1 alpha subcomplex, 8	[2]
Ndufa9	NADH dehydrogenase (ubiquinone) 1 alpha subcomplex, 9	[2]
Ndufab1	NADH dehydrogenase (ubiquinone) 1, alpha/beta subcomplex, 1	[2]
Ndufb10	NADH dehydrogenase (ubiquinone) 1 beta subcomplex, 10	[2]

Proteins from previous mouse sperm proteomes		
**Protein Symbol**	**Protein Name**	**Reference**
Ndufb4	NADH dehydrogenase (ubiquinone) 1 beta subcomplex 4	[2]
Ndufb5	NADH dehydrogenase (ubiquinone) 1 beta subcomplex, 5	[2]
Ndufb7	NADH dehydrogenase (ubiquinone) 1 beta subcomplex, 7	[2]
Ndufc2	NADH dehydrogenase (ubiquinone) 1, subcomplex unknown, 2	[2]
Ndufs1	NADH dehydrogenase (ubiquinone) Fe-S protein 1	[2]
Ndufs2	NADH dehydrogenase (ubiquinone) Fe-S protein 2	[2]
Ndufs3	NADH dehydrogenase (ubiquinone) Fe-S protein 3	[2]
Ndufs4	NADH dehydrogenase (ubiquinone) Fe-S protein 4	[2]
Ndufs5	NADH dehydrogenase (ubiquinone) Fe-S protein 5	[2]
Ndufs6	NADH dehydrogenase (ubiquinone) Fe-S protein 6	[2]
Ndufs7	NADH dehydrogenase (ubiquinone) Fe-S protein 7	[2]
Ndufv1	NADH dehydrogenase (ubiquinone) flavoprotein 1	[2]
Ndufv2	NADH dehydrogenase (ubiquinone) flavoprotein 2	[2]
Nfs1	nitrogen fixation gene 1 (S. cerevisiae)	[2]
Nhlrc1	NHL repeat containing 1	[2]
Niban	non-metastatic cells 2, protein (NM23B) expressed in	[3]
Nipbl	Nipped-B homolog (Drosophila)	[2]
Nipsnap3a	nipsnap homolog 3A (C. elegans)	[2]
Nme2	non-metastatic cells 2, protein (NM23B) expressed in	[2],[3]
Nme5	non-metastatic cells 5, protein expressed in (nucleoside-diphosphate kinase)	[2]
Nme7	non-metastatic cells 7, protein expressed in (nucleoside-diphosphate kinase)	[2]
Nsf	N-ethylmaleimide sensitive fusion protein	[2]
Nt5c1b	5'-nucleotidase, cytosolic IB	[2]
Nup153	nucleoporin 153	[2]
Nup210l	nucleoporin 210-like	[2]
Nup54	nucleoporin 54	[2]
Odf1	outer dense fiber of sperm tails 1	[2]
Odf2	outer dense fiber of sperm tails 2	[2],[3]
Odf3	outer dense fiber of sperm tails 3	[2]
Ogdh	oxoglutarate dehydrogenase (lipoamide)	[2]
Ogdhl	oxoglutarate dehydrogenase-like	[2]
Olfr504	olfactory receptor 504	[2]
Olfr905	olfactory receptor 905	[2]
Oplah	5-oxoprolinase (ATP-hydrolysing)	[2]

Proteins from previous mouse sperm proteomes		
**Protein Symbol**	**Protein Name**	**Reference**
OTTMUSG00000005300	predicted gene 12070	[3]
Oxct2a	3-oxoacid CoA transferase 2A	[3]
Oxct2b	3-oxoacid CoA transferase 2B	[2],[3]
P4hb	prolyl 4-hydroxylase, beta polypeptide	[1],[3]
Pacrg	PARK2 co-regulated	[2]
Park7	Parkinson disease (autosomal recessive, early onset) 7	[2]
Pcdh7	protocadherin 7	[2]
Pclo	piccolo (presynaptic cytomatrix protein)	[2]
Pcmt1	protein-L-isoaspartate (D-aspartate) O-methyltransferase 1	[2]
Pcp4	Purkinje cell protein 4	[3]
Pcsk6	proprotein convertase subtilisin/kexin type 6	[2]
Pcx	pyruvate carboxylase	[2]
Pcyox1	prenylcysteine oxidase 1	[1]
Pde1b	phosphodiesterase 1B, Ca2+-calmodulin dependent	[2]
Pdha1	pyruvate dehydrogenase E1 alpha 1	[2]
Pdha2	pyruvate dehydrogenase E1 alpha 2	[2],[3]
Pdhb	pyruvate dehydrogenase (lipoamide) beta	[2],[3]
Pdhx	pyruvate dehydrogenase complex, component X	[2]
Pdia3	protein disulfide isomerase associated 3	[1],[3]
Pdia4	protein disulfide isomerase associated 4	[1],[2]
Pdia6	protein disulfide isomerase associated 6	[1],[3]
Pdpk1	3-phosphoinositide dependent protein kinase-1	[2]
Pdzk1	PDZ domain containing 1	[2]
Pebp1	phosphatidylethanolamine binding protein 1	[2],[3]
Pfkm	phosphofructokinase, muscle	[2]
Pfkp	phosphofructokinase, platelet	[2]
Pfn1	profilin 1	[3]
Pfn3	profilin 3	[2]
Pgam1	phosphoglycerate mutase 1	[3]
Pgam2	phosphoglycerate mutase 2	[2],[3]
Pgcp	plasma glutamate carboxypeptidase	[1]
Pgd	phosphogluconate dehydrogenase	[2]
Pgk1	phosphoglycerate kinase 1	[3]
Pgk2	phosphoglycerate kinase 2	[2],[3]

Proteins from previous mouse sperm proteomes		
**Protein Symbol**	**Protein Name**	**Reference**
Pgrmc1	progesterone receptor membrane component 1	[1],[2]
Pgs1	phosphatidylglycerophosphate synthase 1	[2]
Phb	prohibitin	[2]
Phb2	prohibitin 2	[2]
Phospho1	phosphatase, orphan 1	[2]
Pik3r3	phosphatidylinositol 3 kinase, regulatory subunit, polypeptide 3 (p55)	[2]
Pik3r4	phosphatidylinositol 3 kinase, regulatory subunit, polypeptide 4, p150	[2]
Pitrm1	pitrilysin metallepetidase 1	[2]
Pkm2	pyruvate kinase, muscle	[2],[3]
Plb1	phospholipase B1	[1],[2]
Plbd1	phospholipase B domain containing 1	[1],[2],[3]
Plg	plasminogen	[3]
Pls3	plastin 3 (T-isoform)	[3]
Pnpla7	patatin-like phospholipase domain containing 7	[2]
Ppap2a	phosphatidic acid phosphatase type 2A	[1],[2]
Ppia	peptidylprolyl isomerase A	[3]
Ppm1b	protein phosphatase 1B, magnesium dependent, beta isoform	[2]
Ppp1cc	protein phosphatase 1, catalytic subunit, gamma isoform	[2],[3]
Ppp1r11	protein phosphatase 1, regulatory (inhibitor) subunit 11	[2]
Ppp1r7	protein phosphatase 1, regulatory (inhibitor) subunit 7	[2]
Ppp2r1a	protein phosphatase 2 (formerly 2A), regulatory subunit A (PR 65), alpha isoform	[2]
Ppp3cc	protein phosphatase 3, catalytic subunit, gamma isoform	[2]
Ppp3r2	protein phosphatase 3, regulatory subunit B, alpha isoform (calcineurin B, type II)	[2]
Ppp6c	protein phosphatase 6, catalytic subunit	[2]
Prcp	prolylcarboxypeptidase (angiotensinase C)	[1],[2]
Prdx1	peroxiredoxin 1	[3]
Prdx2	peroxiredoxin 2	[3]
Prdx5	peroxiredoxin 5	[2],[3]
Prdx6	peroxiredoxin 6	[2],[3]
Prdx6-rs1	peroxiredoxin 6, related sequence 1	[2]
Prkaca	protein kinase, cAMP dependent, catalytic, alpha	[2]
Prkar1a	protein kinase, cAMP dependent regulatory, type I, alpha	[2]
Prkar2a	protein kinase, cAMP dependent regulatory, type II alpha	[2],[3]

Proteins from previous mouse sperm proteomes		
**Protein Symbol**	**Protein Name**	**Reference**
Prkcsh	protein kinase C substrate 80K-H	[1],[3]
Prkrir	protein-kinase, interferon-inducible double stranded RNA dependent inhibitor, repressor of (P58 repressor)	[2]
Prm2	protamine 2	[2]
Prom1	prominin 1	[1]
Prpsap2	phosphoribosyl pyrophosphate synthetase-associated protein 2	[2]
Prss21	protease, serine, 21	[1]
Prss32	protease, serine, 32	[2]
Psip1	PC4 and SFRS1 interacting protein 1	[2]
Psma1	proteasome (prosome, macropain) subunit, alpha type 1	[2]
Psma2	proteasome (prosome, macropain) subunit, alpha type 2	[2]
Psma3	proteasome (prosome, macropain) subunit, alpha type 3	[2]
Psma5	proteasome (prosome, macropain) subunit, alpha type 5	[2]
Psma6	proteasome (prosome, macropain) subunit, alpha type 6	[2]
Psma8	proteasome (prosome, macropain) subunit, alpha type, 8	[2]
Psmb1	proteasome (prosome, macropain) subunit, beta type 1	[2]
Psmb2	proteasome (prosome, macropain) subunit, beta type 2	[2]
Psmb3	proteasome (prosome, macropain) subunit, beta type 3	[2]
Psmb4	proteasome (prosome, macropain) subunit, beta type 4	[2]
Psmb5	proteasome (prosome, macropain) subunit, beta type 5	[2]
Psmb6	proteasome (prosome, macropain) subunit, beta type 6	[2]
Psmb7	proteasome (prosome, macropain) subunit, beta type 7	[2]
Psmc1	protease (prosome, macropain) 26S subunit, ATPase 1	[2]
Psmc2	proteasome (prosome, macropain) 26S subunit, ATPase 2	[2]
Psmc3	proteasome (prosome, macropain) 26S subunit, ATPase 3	[2]
Psmc4	proteasome (prosome, macropain) 26S subunit, ATPase, 4	[2]
Psmc5	protease (prosome, macropain) 26S subunit, ATPase 5	[2]
Psmc6	proteasome (prosome, macropain) 26S subunit, ATPase, 6	[2]
Psmd1	proteasome (prosome, macropain) 26S subunit, non-ATPase, 1	[2]
Psmd11	proteasome (prosome, macropain) 26S subunit, non-ATPase, 11	[2]
Psmd13	proteasome (prosome, macropain) 26S subunit, non-ATPase, 13	[2]
Psmd14	proteasome (prosome, macropain) 26S subunit, non-ATPase, 14	[2]
Psmd2	proteasome (prosome, macropain) 26S subunit, non-ATPase, 2	[2]
Psmd3	proteasome (prosome, macropain) 26S subunit, non-ATPase, 3	[2]
Psmd6	proteasome (prosome, macropain) 26S subunit, non-ATPase, 6	[2]

Proteins from previous mouse sperm proteomes		
**Protein Symbol**	**Protein Name**	**Reference**
Psmd7	proteasome (prosome, macropain) 26S subunit, non-ATPase, 7	[2]
Psmd8	proteasome (prosome, macropain) 26S subunit, non-ATPase, 8	[2]
Psme1	proteasome (prosome, macropain) 28 subunit, alpha	[3]
Ptchd3	patched domain containing 3	[1],[2]
Ptgds	prostaglandin D2 synthase (brain)	[3]
ptgs1	prostaglandin-endoperoxide synthase 1	[1],[3]
Ptgs2	prostaglandin-endoperoxide synthase 2	[3]
Ptrh2	peptidyl-tRNA hydrolase 2	[2]
Pvrl3	poliovirus receptor-related 3	[2]
Pzp	pregnancy zone protein	[3]
Qsox1	quiescin Q6 sulfhydryl oxidase 1	[1],[3]
Rab10	RAB10, member RAS oncogene family	[3]
Rab11b	RAB11B, member RAS oncogene family	[2]
Rab14	RAB14, member RAS oncogene family	[2]
Rab22a	RAB22A, member RAS oncogene family	[2]
Rab2a	RAB2A, member RAS oncogene family	[2]
Rab3gap2	RAB3 GTPase activating protein subunit 2	[2]
Rab5c	RAB5C, member RAS oncogene family	[2]
Rab8a	RAB8A, member RAS oncogene family	[2]
Rab8b	RAB8B, member RAS oncogene family	[2]
Rabggtb	RAB geranylgeranyl transferase, b subunit	[2]
Rad17	RAD17 homolog (S. pombe)	[2]
Rae1	RAE1 RNA export 1 homolog (S. pombe)	[2]
Rala	v-ral simian leukemia viral oncogene homolog A (ras related)	[2]
Rangrf	RAN guanine nucleotide release factor	[2]
Rbm12	RNA binding motif protein 12	[2]
Rcn1	reticulocalbin 1	[3]
Reep6	receptor accessory protein 6	[2]
Rfx7	regulatory factor X, 7	[2]
Rgs22	regulator of G-protein signalling 22	[2]
Ribc2	RIB43A domain with coiled-coils 2	[2]
Rif1	Rap1 interacting factor 1 homolog (yeast)	[2]
Rnls	renalase, FAD-dependent amine oxidase	[2]
Ropn1	ropporin, rhophilin associated protein 1	[2],[3]

Proteins from previous mouse sperm proteomes		
**Protein Symbol**	**Protein Name**	**Reference**
Ropn1l	ropporin 1-like	[2]
Rpn1	ribophorin I	[1]
Rpn2	ribophorin II	[1]
Rprml	reprimo-like	[2]
Rps27a	ribosomal protein S27A	[3]
Rrbp1	ribosome binding protein 1	[1]
Rshl1	radial spokehead-like 1	[2]
Rshl2b	radial spokehead-like 2B	[2]
Rsph1	radial spoke head 1 homolog (Chlamydomonas)	[2]
Rsph9	radial spoke head 9 homolog (Chlamydomonas)	[2]
Rtdr1	rhabdoid tumor deletion region gene 1	[2]
S100a6	S100 calcium binding protein A6 (calcyclin)	[3]
Sacm1l	SAC1 (suppressor of actin mutations 1, homolog)-like (S. cerevisiae)	[1]
Samm50	sorting and assembly machinery component 50 homolog (S. cerevisiae)	[2]
Satl1	spermidine/spermine N1-acetyl transferase-like 1	[2]
Scamp1	secretory carrier membrane protein 1	[2]
Scamp2	secretory carrier membrane protein 2	[1],[2]
Sccpdh	saccharopine dehydrogenase (putative)	[1],[2],[3]
Scnn1b	sodium channel, nonvoltage-gated 1 beta	[2]
Sdccag8	serologically defined colon cancer antigen 8	[2]
Sdha	succinate dehydrogenase complex, subunit A, flavoprotein (Fp)	[2],[3]
Sdhb	succinate dehydrogenase complex, subunit B, iron sulfur (Ip)	[2]
Sdhd	succinate dehydrogenase complex, subunit D, integral membrane protein	[2]
Sema6b	sema domain, transmembrane domain (TM), and cytoplasmic domain, (semaphorin) 6B	[2]
Senp8	SUMO/sentrin specific peptidase 8	[2]
Sept4	septin 4	[2]
Sept7	septin 7	[2]
Serpina1a	serine (or cysteine) peptidase inhibitor, clade A, member 1A	[3]
Serpina1b	serine (or cysteine) preptidase inhibitor, clade A, member 1B	[1],[3]
Serpina1d	serine (or cysteine) peptidase inhibitor, clade A, member 1D	[3]
Serpina1e	serine (or cysteine) peptidase inhibitor, clade A, member 1E	[1],[3]
Serpina1f	serine (or cysteine) peptidase inhibitor, clade A, member 1F	[1],[2],[3]
Serpina3k	serine (or cysteine) peptidase inhibitor, clade A, member 3K	[1],[3]
Serpina5	serine (or cysteine) peptidase inhibitor, clade A, member 5	[2]

Proteins from previous mouse sperm proteomes		
**Protein Symbol**	**Protein Name**	**Reference**
Serpinb6a	serine (or cysteine) peptidase inhibitor, clade B, member 6a	[2]
Sgta	small glutamine-rich tetratricopeptide repeat (TPR)-containing, alpha	[2]
Sh3bgrl	SH3-binding domain glutamic acid-rich protein like	[3]
Shmt1	serine hydroxymethyltransferase 1 (soluble)	[2]
Skiv2l	superkiller viralicidic activity 2-like (S. cerevisiae)	[2]
Slc16a7	solute carrier family 16 (monocarboxylic acid transporters), member 7	[1],[2]
Slc22a21	solute carrier family 22 (organic cation transporter), member 21	[1]
Slc22a5	solute carrier family 22 (organic cation transporter), member 5	[1]
Slc25a10	solute carrier family 25 (mitochondrial carrier, dicarboxylate transporter), member 10	[2]
Slc25a3	solute carrier family 25 (mitochondrial carrier, phosphate carrier), member 3	[2]
Slc25a31	solute carrier family 25 (mitochondrial carrier; adenine nucleotide translocator), member 31	[2],[3]
Slc25a4	solute carrier family 25 (mitochondrial carrier, adenine nucleotide translocator), member 4	[2]
Slc26a8	solute carrier family 26, member 8	[1]
Slc2a3	solute carrier family 2 (facilitated glucose transporter), member 3	[1],[2],[3]
Slc2a5	solute carrier family 2 (facilitated glucose transporter), member 5	[1],[2]
Smcp	sperm mitochondria-associated cysteine-rich protein	[2]
Sod1	superoxide dismutase 1, soluble	[2],[3]
Sod2	superoxide dismutase 2, mitochondrial	[2]
Sord	sorbitol dehydrogenase	[2],[3]
Sort1	sortilin 1	[1]
Spa17	sperm autoantigenic protein 17	[2]
Spaca1	sperm acrosome associated 1	[1],[2]
Spaca3	sperm acrosome associated 3	[2]
Spaca5	sperm acrosome associated 5	[2]
Spag16	sperm associated antigen 16	[2]
Spag17	sperm associated antigen 17	[2]
Spag6	sperm associated antigen 6	[2]
Spam1	sperm adhesion molecule 1	[2]
Spata18	spermatogenesis associated 18	[2]
Spata19	spermatogenesis associated 19	[2]
Spata6	spermatogenesis associated 6	[2]
Spatc1	spermatogenesis and centriole associated 1	[2]
Spcs2	signal peptidase complex subunit 2 homolog (S. cerevisiae)	[1]

Proteins from previous mouse sperm proteomes		
**Protein Symbol**	**Protein Name**	**Reference**
Spesp1	sperm equatorial segment protein 1	[1],[2]
Spg20	spastic paraplegia 20, spartin (Troyer syndrome) homolog (human)	[2]
Spink5	serine peptidase inhibitor, Kazal type 5	[3]
Spnb2	spectrin beta 2	[2]
Sqrdl	sulfide quinone reductase-like (yeast)	[2]
Srpk2	serine/arginine-rich protein specific kinase 2	[2]
Srpk3	serine/arginine-rich protein specific kinase 3	[2]
Ssna1	Sjogren's syndrome nuclear autoantigen 1	[2]
St13	suppression of tumorigenicity 13	[2]
Stard6	StAR-related lipid transfer (START) domain containing 6	[2]
Stub1	STIP1 homology and U-Box containing protein 1	[2]
Stxbp3a	syntaxin binding protein 3A	[2]
Sucla2	succinate-Coenzyme A ligase, ADP-forming, beta subunit	[2],[3]
Suclg1	succinate-CoA ligase, GDP-forming, alpha subunit	[2],[3]
Suox	sulfite oxidase	[2]
Sytl2	synaptotagmin-like 2	[2]
Tagln	transgelin	[3]
Tagln2	transgelin 2	[3]
Tbc1d17	TBC1 domain family, member 17	[2]
Tbc1d20	TBC1 domain family, member 20	[2]
Tbl1x	transducin (beta)-like 1 X-linked	[2]
Tbl2	transducin (beta)-like 2	[2]
Tcp1	t-complex protein 1	[2]
Tekt1	tektin 1	[2]
Tekt2	tektin 2	[2]
Tekt3	tektin 3	[2]
Tekt4	tektin 4	[2]
Tekt5	tektin 5	[2]
Tepp	testis, prostate and placenta expressed	[2]
Tex101	testis expressed gene 101	[2]
Thap4	THAP domain containing 4	[2]
Theg	testicular haploid expressed gene	[2]
Them4	thioesterase superfamily member 4	[2]
Thnsl1	threonine synthase-like 1 (bacterial)	[2]

Proteins from previous mouse sperm proteomes		
**Protein Symbol**	**Protein Name**	**Reference**
Timm44	translocase of inner mitochondrial membrane 44	[2]
Timm50	translocase of inner mitochondrial membrane 50 homolog (yeast)	[2]
Tkt	transketolase	[3]
Tmed10	transmembrane emp24-like trafficking protein 10 (yeast)	[2]
Tmem146	transmembrane protein 146	[2]
Tmem147	transmembrane protein 147	[2]
Tmem177	transmembrane protein 177	[2]
Tmem190	transmembrane protein 190	[1],[2]
Tmem30a	transmembrane protein 30A	[2]
Tnr	tenascin R	[2]
Tomm22	translocase of outer mitochondrial membrane 22 homolog (yeast)	[2]
Tomm40	translocase of outer mitochondrial membrane 40 homolog (yeast)	[2]
Tpi1	triosephosphate isomerase 1	[2],[3]
Tpm1	tropomyosin 1, alpha	[3]
Tpm3	tropomyosin 3, gamma	[3]
Tpp2	tripeptidyl peptidase II	[2]
Trappc6b	trafficking protein particle complex 6B	[2]
Trf	transferrin	[3]
Trpc3	transient receptor potential cation channel, subfamily C, member 3	[2]
try10	trypsin 10	[3]
Tsga10	testis specific 10	[2]
Tsp50	testes-specific protease 50	[1]
Tssk1	testis-specific serine kinase 1	[2]
Tssk4	testis-specific serine kinase 4	[2]
Tssk6	testis-specific serine kinase 6	[2]
Ttc25	tetratricopeptide repeat domain 25	[2]
Ttc39d	tetratricopeptide repeat domain 39D	[2]
Ttc8	tetratricopeptide repeat domain 8	[2]
Ttn	titin	[2]
Ttr	transthyretin	[3]
Tuba1a	tubulin, alpha 1A	[2],[3]
Tuba1b	tubulin, alpha 1B	[3]
Tuba1c	tubulin, alpha 1C	[3]
Tuba3a	tubulin, alpha 3A	[3]

Proteins from previous mouse sperm proteomes		
**Protein Symbol**	**Protein Name**	**Reference**
Tuba3b	tubulin, alpha 3B	[2],[3]
Tubb2b	tubulin, beta 2B	[2]
Tubb2c	tubulin, beta 2C	[2],[3]
Tubb3	tubulin, beta 3	[2]
Tubb4	tubulin, beta 4	[2]
Txndc3	thioredoxin domain containing 3 (spermatozoa)	[2]
Uba52	ubiquitin A-52 residue ribosomal protein fusion product 1	[2]
Uba6	ubiquitin-like modifier activating enzyme 6	[2]
Ubb	ubiquitin B	[3]
Ube1x	ubiquitin-like modifier activating enzyme 1	[2],[3]
Ube2v2	ubiquitin-conjugating enzyme E2 variant 2	[2]
Ubqln1	ubiquilin 1	[2]
Ubtd2	ubiquitin domain containing 2	[2]
Ubxn11	UBX domain protein 11	[2]
Ubxn8	UBX domain protein 8	[2]
Uchl1	ubiquitin carboxy-terminal hydrolase L1	[2]
Uchl3	ubiquitin carboxyl-terminal esterase L3 (ubiquitin thiolesterase)	[2]
Uchl4	ubiquitin carboxyl-terminal esterase L4	[2]
Uchl5	ubiquitin carboxyl-terminal esterase L5	[2]
Ugcgl1	UDP-glucose glycoprotein glucosyltransferase 1	[1],[3]
Ugp2	UDP-glucose pyrophosphorylase 2	[2]
Uqcr	ubiquinol-cytochrome c reductase (6.4kD) subunit	[2]
Uqcrb	ubiquinol-cytochrome c reductase binding protein	[2]
Uqcrc1	ubiquinol-cytochrome c reductase core protein 1	[2]
Uqcrc2	ubiquinol cytochrome c reductase core protein 2	[2]
Uqcrfs1	ubiquinol-cytochrome c reductase, Rieske iron-sulfur polypeptide 1	[2]
Uqcrq	ubiquinol-cytochrome c reductase, complex III subunit VII	[2]
Usp4	ubiquitin specific peptidase 4 (proto-oncogene)	[2]
Usp7	ubiquitin specific peptidase 7	[2]
Vamp3	vesicle-associated membrane protein 3	[2]
Vapa	vesicle-associated membrane protein, associated protein A	[2]
Vcl	vinculin	[3]
Vcp	valosin containing protein	[2],[3]
Vdac1	voltage-dependent anion channel 1	[2]

Proteins from previous mouse sperm proteomes		
**Protein Symbol**	**Protein Name**	**Reference**
Vdac2	voltage-dependent anion channel 2	[2]
Vdac3	voltage-dependent anion channel 3	[2]
Vil2	ezrin	[3]
Vim	vimentin	[3]
Vps13a	vacuolar protein sorting 13A (yeast)	[2]
Wars	tryptophanyl-tRNA synthetase	[3]
Was	Wiskott-Aldrich syndrome homolog (human)	[2]
Wbp7	WW domain binding protein 7	[2]
Wdr16	WD repeat domain 16	[2]
Wdr49	WD repeat domain 49	[2]
Wdr52	WD repeat domain 52	[2]
Wdr64	WD repeat domain 64	[2]
Wnk2	WNK lysine deficient protein kinase 2	[2]
Xpnpep1	X-prolyl aminopeptidase (aminopeptidase P) 1, soluble	[2]
Xpo7	exportin 7	[2]
Ywhab	tyrosine 3-monooxygenase/tryptophan 5-monooxygenase activation protein, beta polypeptide	[2],[3]
Ywhae	tyrosine 3-monooxygenase/tryptophan 5-monooxygenase activation protein, epsilon polypeptide	[2],[3]
Ywhaz	tyrosine 3-monooxygenase/tryptophan 5-monooxygenase activation protein, zeta polypeptide	[2],[3]
Zan	zonadhesin	[1],[2],[3]
Zfp112	zinc finger protein 112	[2]
Zfp395	zinc finger protein 395	[2]
Zfp407	zinc finger protein 407	[2]
Zp3r	zona pellucida 3 receptor	[1],[2],[3]
Zpbp	zona pellucida binding protein	[1],[2]
Zpbp2	zona pellucida binding protein 2	[1],[2]

References: [1] Stein et al. 2006, [2] Baker et al. 2008, [3] Dorus et al. 2010.

Additional proteins compìled in this study	
**Protein Symbol**	**Protein Name**
Acpp	acid phosphatase, prostate
Acrbp	proacrosin binding protein
Actg1	actin, gamma, cytoplasmic 1
Actg2	actin, gamma 2, smooth muscle, enteric
Adad1	adenosine deaminase domain containing 1 (testis specific)
Adam15	a disintegrin and metallopeptidase domain 15 (metargidin)
Adam17	a disintegrin and metallopeptidase domain 17
Adam18	a disintegrin and metallopeptidase domain 18
Adam1a	a disintegrin and metallopeptidase domain 1a
Adam1b	a disintegrin and metallopeptidase domain 1b
Adam21	a disintegrin and metallopeptidase domain 21
Adam23	a disintegrin and metallopeptidase domain 23
Adam25	a disintegrin and metallopeptidase domain 25 (testase 2)
Adam26a	a disintegrin and metallopeptidase domain 26A (testase 3)
Adam28	a disintegrin and metallopeptidase domain 28
Adam29	a disintegrin and metallopeptidase domain 29
Adam32	a disintegrin and metallopeptidase domain 32
Adam7	a disintegrin and metallopeptidase domain 7
Adcy10	adenylate cyclase 10
Adcy3	adenylate cyclase 3
Adcy8	adenylate cyclase 8
Agfg1	ArfGAP with FG repeats 1
Agtpbp1	ATP/GTP binding protein 1
Akap1	A kinase (PRKA) anchor protein 1
Akap10	A kinase (PRKA) anchor protein 10
Akap11	A kinase (PRKA) anchor protein 11
Akap12	A kinase (PRKA) anchor protein (gravin) 12
Akap8	A kinase (PRKA) anchor protein 8
Atm	ataxia telangiectasia mutated homolog (human)
Atp2b4	ATPase, Ca++ transporting, plasma membrane 4
B4galt1	UDP-Gal:betaGlcNAc beta 1,4- galactosyltransferase, polypeptide 1
Bag6	BCL2-associated athanogene 6

Additional proteins compiled in this study	
**Protein Symbol**	**Protein Name**
Bax	BCL2-associated X protein
Bbs2	Bardet-Biedl syndrome 2 (human)
Bbs4	Bardet-Biedl syndrome 4 (human)
BC100451	cDNA sequence BC100451
Boll	bol, boule-like (Drosophila)
Bpnt1	bisphosphate 3'-nucleotidase 1
Brs3	bombesin-like receptor 3
Cadm1	cell adhesion molecule 1
Calca	calcitonin/calcitonin-related polypeptide, alpha
Calcb	calcitonin-related polypeptide, beta
Camk2b	calcium/calmodulin-dependent protein kinase II, beta
Camk2d	calcium/calmodulin-dependent protein kinase II, delta
Camp	cathelicidin antimicrobial peptide
Catsper1	cation channel, sperm associated 1
Catsper2	cation channel, sperm associated 2
Catsper3	cation channel, sperm associated 3
Catsperb	catsper channel auxiliary subunit beta
Catsperd	catsper channel auxiliary subunit delta
Catsperg2	catsper channel auxiliary subunit gamma 2
Cav1	caveolin 1, caveolae protein
Cav2	caveolin 2
Cav3	caveolin 3
Ccdc54	coiled-coil domain containing 54
Ccna1	cyclin A1
Ccnd1	cyclin D1
Cd151	CD151 antigen
Cd52	CD52 antigen
Cd59a	CD59a antigen
Cd59b	CD59b antigen
Cdk2	cyclin-dependent kinase 2
Ceacam10	carcinoembryonic antigen-related cell adhesion molecule 10
Celf3	CUGBP, Elav-like family member 3
Chrna7	cholinergic receptor, nicotinic, alpha polypeptide 7
Clgn	calmegin

Additional proteins compiled in this study	
**Protein Symbol**	**Protein Name**
Cnga3	cyclic nucleotide gated channel alpha 3
Cngb1	cyclic nucleotide gated channel beta 1
Cpeb1	cytoplasmic polyadenylation element binding protein 1
Creb3	cAMP responsive element binding protein 3
Creb3l4	cAMP responsive element binding protein 3-like 4
Crisp1	cysteine-rich secretory protein 1
Crisp2	cysteine-rich secretory protein 2
Crisp3	cysteine-rich secretory protein 3
Crisp4	cysteine-rich secretory protein 4
Csk	c-src tyrosine kinase
Cst13	cystatin 13
Cst8	cystatin 8 (cystatin-related epididymal spermatogenic)
Cstf2	cleavage stimulation factor, 3' pre-RNA subunit 2
Ctsd	cathepsin D
Ctsl	cathepsin L
Cttn	cortactin
D3Bwg0562e	DNA segment, Chr 3, Brigham & Women's Genetics 0562 expressed
Dazap1	DAZ associated protein 1
Dazl	deleted in azoospermia-like
Dbi	diazepam binding inhibitor
Ddx25	DEAD (Asp-Glu-Ala-Asp) box polypeptide 25
Ddx4	DEAD (Asp-Glu-Ala-Asp) box polypeptide 4
Defb19	defensin beta 19
Defb22	defensin beta 22
Dffa	DNA fragmentation factor, alpha subunit
Dkkl1	dickkopf-like 1
Dnaaf1	dynein, axonemal assembly factor 1
Dnahc1	dynein, axonemal, heavy chain 1
Dnahc12	dynein, axonemal, heavy chain 12
Dnahc17	dynein, axonemal, heavy chain 17
Dnahc2	dynein, axonemal, heavy chain 2
Dnahc3	dynein, axonemal, heavy chain 3
Dnahc7a	dynein, axonemal, heavy chain 7A
Dnaja1	DnaJ (Hsp40) homolog, subfamily A, member 1

Additional proteins compiled in this study	
**Protein Symbol**	**Protein Name**
Dnmt3l	DNA (cytosine-5-)-methyltransferase 3-like
Dock1	dedicator of cytokinesis 1
Dpcd	deleted in primary ciliary dyskinesia
Dynlt1b	dynein light chain Tctex-type 1B
Egfl7	EGF-like domain 7
Eif4g3	eukaryotic translation initiation factor 4 gamma, 3
Eqtn	equatorin, sperm acrosome associated
Esx1	extraembryonic, spermatogenesis, homeobox 1
Etv5	ets variant gene 5
Fam65c	family with sequence similarity 65, member C
Fancl	Fanconi anemia, complementation group L
Fer	fer (fms/fps related) protein kinase
Fgfr1	fibroblast growth factor receptor 1
Fkbp10	FK506 binding protein 10
Fkbp6	FK506 binding protein 6
Fscn1	fascin homolog 1, actin bundling protein (Strongylocentrotus purpuratus)
Fyn	Fyn proto-oncogene
Gadd45a	growth arrest and DNA-damage-inducible 45 alpha
Galnt3	UDP-N-acetyl-alpha-D-galactosamine:polypeptide N-acetylgalactosaminyltransferase 3
Ggn	gametogenetin
Glra1	glycine receptor, alpha 1 subunit
Glra2	glycine receptor, alpha 2 subunit
Glrb	glycine receptor, beta subunit
Gm5803	predicted gene 5803
Gopc	golgi associated PDZ and coiled-coil motif containing
Gsg1	germ cell-specific gene 1
Gsk3b	glycogen synthase kinase 3 beta
Gusb	glucuronidase, beta
H1fnt	H1 histone family, member N, testis-specific
H2afx	H2A histone family, member X
Hc	hemolytic complement
Hils1	histone H1-like protein in spermatids 1
Hnrnpa1	heterogeneous nuclear ribonucleoprotein A1

Additional proteins compìled in this study	
**Protein Symbol**	**Protein Name**
Hsp90b1	heat shock protein 90, beta (Grp94), member 1
Hspa1l	heat shock protein 1-like
Hyal3	hyaluronoglucosaminidase 3
Hyal6	hyaluronoglucosaminidase 6
Ier3	immediate early response 3
Ift88	intraflagellar transport 88
Igsf8	immunoglobulin superfamily, member 8
Il5	interleukin 5
Inpp5b	inositol polyphosphate-5-phosphatase B
Insl6	insulin-like 6
Itga3	integrin alpha 3
Itga6	integrin alpha 6
Itgb1	integrin beta 1 (fibronectin receptor beta)
Itpr1	inositol 1,4,5-trisphosphate receptor 1
Kcnu1	potassium channel, subfamily U, member 1
Kdm3a	lysine (K)-specific demethylase 3A
Kdm5b	lysine (K)-specific demethylase 5B
Khdrbs1	KH domain containing, RNA binding, signal transduction associated 1
Kit	kit oncogene
Klk1b27	kallikrein 1-related peptidase b27
Klkb1	kallikrein B, plasma 1
Krt9	keratin 9
Lamb1	laminin B1
Mast2	microtubule associated serine/threonine kinase 2
Mcts2	malignant T cell amplified sequence 2
Mei1	meiosis defective 1
Meig1	meiosis expressed gene 1
Mfge8	milk fat globule-EGF factor 8 protein
Mgea5	meningioma expressed antigen 5 (hyaluronidase)
Mif4gd	MIF4G domain containing
Mkks	McKusick-Kaufman syndrome
Mlh1	mutL homolog 1 (E. coli)
Mlh3	mutL homolog 3 (E coli)
Mmp1a	matrix metallopeptidase 1a (interstitial collagenase)

Additional proteins compiled in this study	
**Protein Symbol**	**Protein Name**
Ms4a13	membrane-spanning 4-domains, subfamily A, member 13
Msh4	mutS homolog 4 (E. coli)
Msh5	mutS homolog 5 (E. coli)
Msmb	beta-microseminoprotein
Nanos1	nanos homolog 1 (Drosophila)
Nanos2	nanos homolog 2 (Drosophila)
Nanos3	nanos homolog 3 (Drosophila)
Nckipsd	NCK interacting protein with SH3 domain
Neu1	neuraminidase 1
Neurl1a	neuralized homolog 1A (Drosophila)
Nkx3-1	NK-3 transcription factor, locus 1 (Drosophila)
Nme8	NME/NM23 family member 8
Npepps	aminopeptidase puromycin sensitive
Nsun4	NOL1/NOP2/Sun domain family, member 4
Nup214	nucleoporin 214
Odf4	outer dense fiber of sperm tails 4
Ovgp1	oviductal glycoprotein 1
Pafah1b1	platelet-activating factor acetylhydrolase, isoform 1b, subunit 1
Papolb	poly (A) polymerase beta (testis specific)
Parg	poly (ADP-ribose) glycohydrolase
Park2	Parkinson disease (autosomal recessive, juvenile) 2, parkin
Parp1	poly (ADP-ribose) polymerase family, member 1
Parp2	poly (ADP-ribose) polymerase family, member 2
Pbp2	phosphatidylethanolamine binding protein 2
Pcsk4	proprotein convertase subtilisin/kexin type 4
Pde11a	phosphodiesterase 11A
Pde1a	phosphodiesterase 1A, calmodulin-dependent
Pde4a	phosphodiesterase 4A, cAMP specific
Pfkfb4	6-phosphofructo-2-kinase/fructose-2,6-biphosphatase 4
Pfn4	profilin family, member 4
Pgrmc2	progesterone receptor membrane component 2
Phf1	PHD finger protein 1
Pick1	protein interacting with C kinase 1

Additional proteins compiled in this study	
**Protein Symbol**	**Protein Name**
Pik3ca	phosphatidylinositol 3-kinase, catalytic, alpha polypeptide
Pik3cb	phosphatidylinositol 3-kinase, catalytic, beta polypeptide
Pik3cd	phosphatidylinositol 3-kinase catalytic delta polypeptide
Pik3cg	phosphoinositide-3-kinase, catalytic, gamma polypeptide
Pik3r1	phosphatidylinositol 3-kinase, regulatory subunit, polypeptide 1 (p85 alpha)
Pip	prolactin induced protein
Piwil1	piwi-like RNA-mediated gene silencing 1
Piwil2	piwi-like RNA-mediated gene silencing 2
Piwil4	piwi-like RNA-mediated gene silencing 4
Pkdrej	polycystic kidney disease (polycystin) and REJ (sperm receptor for egg jelly homolog, sea urchin)
Pla2g1b	phospholipase A2, group IB, pancreas
Plag1	pleiomorphic adenoma gene 1
Plau	plasminogen activator, urokinase
Plcd4	phospholipase C, delta 4
Plekha5	pleckstrin homology domain containing, family A member 5
Plscr1	phospholipid scramblase 1
Plscr4	phospholipid scramblase 4
Pmfbp1	polyamine modulated factor 1 binding protein 1
Pms2	postmeiotic segregation increased 2 (S. cerevisiae)
Ppp1r11	protein phosphatase 1, regulatory (inhibitor) subunit 11
Prdm6	PR domain containing 6
Prdx6b	peroxiredoxin 6B
Prkcg	protein kinase C, gamma
Prm1	protamine 1
Pros1	protein S (alpha)
Prrt2	proline-rich transmembrane protein 2
Psat1	phosphoserine aminotransferase 1
Psmc3ip	proteasome (prosome, macropain) 26S subunit, ATPase 3, interacting protein
Ptk2b	PTK2 protein tyrosine kinase 2 beta
Ptpn1	protein tyrosine phosphatase, non-receptor type 1
Pvrl2	poliovirus receptor-related 2
Rab3a	RAB3A, member RAS oncogene family
Rapgef3	Rap guanine nucleotide exchange factor (GEF) 3
Rapgef4	Rap guanine nucleotide exchange factor (GEF) 4

Additional proteins compiled in this study	
**Protein Symbol**	**Protein Name**
Rbmxl2	RNA binding motif protein, X-linked-like 2
Rbmy	RNA binding motif protein, Y chromosome
Rbpj	recombination signal binding protein for immunoglobulin kappa J region
Rec8	REC8 homolog (yeast)
repro2	reproductive mutant 2, JAX Reproductive Mutagenesis Program
repro3	reproductive mutation 3, JAX Reproductive Mutagenesis Program
Rhob	ras homolog gene family, member B
Rhox5	reproductive homeobox 5
Rhpn1	rhophilin, Rho GTPase binding protein 1
Rhpn2	rhophilin, Rho GTPase binding protein 2
Rpph1	ribonuclease P RNA component H1
Ryr3	ryanodine receptor 3
Sash3	SAM and SH3 domain containing 3
Senp6	SUMO/sentrin specific peptidase 6
Sepp1	selenoprotein P, plasma, 1
Serpine2	serine (or cysteine) peptidase inhibitor, clade E, member 2
Siah1a	seven in absentia 1A
Slc16a10	solute carrier family 16 (monocarboxylic acid transporters), member 10
Slc26a8	solute carrier family 26, member 8
Slc2a3	solute carrier family 2 (facilitated glucose transporter), member 3
Slc9a1	solute carrier family 9 (sodium/hydrogen exchanger), member 1
Slc9a5	solute carrier family 9 (sodium/hydrogen exchanger), member 5
Slc9b2	solute carrier family 9, subfamily B (NHA2, cation proton antiporter 2), member 2
Slx	Sycp3 like X-linked
Slxl1	Slx-like 1
Sly	Sycp3 like Y-linked
Smc1b	structural maintenance of chromosomes 1B
Smok1	sperm motility kinase 1
Smok2a	sperm motility kinase 2A
Smok2b	sperm motility kinase 2B
Smok4a	sperm motility kinase 4A
Snap25	synaptosomal-associated protein 25
Spa17	sperm autoantigenic protein 17
Spaca3	sperm acrosome associated 3

Additional proteins compiled in this study	
**Protein Symbol**	**Protein Name**
Spaca4	sperm acrosome associated 4
Spag1	sperm associated antigen 1
Spag11a	sperm associated antigen 11A
Spag4	sperm associated antigen 4
Spag5	sperm associated antigen 5
Spag7	sperm associated antigen 7
Spag8	sperm associated antigen 8
Spag9	sperm associated antigen 9
Spam1	sperm adhesion molecule 1
Spata16	spermatogenesis associated 16
Spata17	spermatogenesis associated 17
Spata20	spermatogenesis associated 20
Spata21	spermatogenesis associated 21
Spata24	spermatogenesis associated 24
Spata3	spermatogenesis associated 3
Spata5	spermatogenesis associated 5
Spata9	spermatogenesis associated 9
Spem1	sperm maturation 1
Sphk1	sphingosine kinase 1
Spinkl	serine protease inhibitor, Kazal type-like
Spo11	SPO11 meiotic protein covalently bound to DSB homolog (S. cerevisiae)
Srgn	serglycin
Srsf2	serine/arginine-rich splicing factor 2
Stx1b	syntaxin 1B
Stx2	syntaxin 2
Suco	SUN domain containing ossification factor
swm2	sperm without mobility 2
Syce1	synaptonemal complex central element protein 1
Syce2	synaptonemal complex central element protein 2
Sycp2	synaptonemal complex protein 2
Sycp3	synaptonemal complex protein 3
Syt1	synaptotagmin I
Syt6	synaptotagmin VI
Syt8	synaptotagmin VIII

Additional proteins compiled in this study	
**Protein Symbol**	**Protein Name**
Sytl1	synaptotagmin-like 1
Taf7l	TAF7-like RNA polymerase II, TATA box binding protein (TBP)-associated factor
Tcp11	t-complex protein 11
Tcte1	t-complex-associated testis expressed 1
Tcte3	t-complex-associated testis expressed 3
Tctex1d1	Tctex1 domain containing 1
Tctex1d2	Tctex1 domain containing 2
Tctex1d4	Tctex1 domain containing 4
Tesk1	testis specific protein kinase 1
Tex11	testis expressed gene 11
Tex14	testis expressed gene 14
Tg(PRG1)18Wlad	transgene insertion 18, Wyeth Lederle/Anne Deatly
Tgm4	transglutaminase 4 (prostate)
Tia1	cytotoxic granule-associated RNA binding protein 1
Tial1	Tia1 cytotoxic granule-associated RNA binding protein-like 1
Timp1	tissue inhibitor of metalloproteinase 1
Tmprss2	transmembrane protease, serine 2
Tnp1	transition protein 1
Tnp2	transition protein 2
Tra2b	transformer 2 beta homolog (Drosophila)
Trappc8	trafficking protein particle complex 8
Trim36	tripartite motif-containing 36
Trip13	thyroid hormone receptor interactor 13
Trpc1	transient receptor potential cation channel, subfamily C, member 1
Trpc2	transient receptor potential cation channel, subfamily C, member 2
Trpc4	transient receptor potential cation channel, subfamily C, member 4
Trpc6	transient receptor potential cation channel, subfamily C, member 6
Trrap	transformation/transcription domain-associated protein
Tsks	testis-specific serine kinase substrate
Tspo	translocator protein
Tssk2	testis-specific serine kinase 2
Tssk3	testis-specific serine kinase 3
Tssk6	testis-specific serine kinase 6
Tuba1a	tubulin, alpha 1A

Additional proteins compiled in this study	
**Protein Symbol**	**Protein Name**
Tuba3a	tubulin, alpha 3A
Tubd1	tubulin, delta 1
Txndc2	thioredoxin domain containing 2 (spermatozoa)
Tyk2	tyrosine kinase 2
Ube2b	ubiquitin-conjugating enzyme E2B
Usp26	ubiquitin specific peptidase 26
Vamp5	vesicle-associated membrane protein 5
Ybx2	Y box protein 2
Yes1	Yamaguchi sarcoma viral (v-yes) oncogene homolog 1
Zcchc8	zinc finger, CCHC domain containing 8
Zfa-ps	zinc finger protein, autosomal, pseudogene
Zfp474	zinc finger protein 474
Zfp828	zinc finger protein 828

Combined list of proteins	
**Protein Symbol**	**Protein Name**
0710001P09 Rik	coiled-coil-helix-coiled-coil-helix domain containing 6
1100001H23Rik	phospholipase B domain containing 1
1110017D15Rik	RIKEN cDNA 1110017D15 gene
1110020P15Rik	RIKEN cDNA 1110020P15 gene
1190002A17Rik	RIKEN cDNA 1190002A17 gene
1500001M20Rik	RIKEN cDNA 1500001M20 gene
1600016N20Rik	RIKEN cDNA 1600016N20 gene
1700001L19Rik	RIKEN cDNA 1700001L19 gene
1700001O22Rik	RIKEN cDNA 1700001O22 gene
1700007K13Rik	RIKEN cDNA 1700007K13 gene
1700009P17Rik	RIKEN cDNA 1700009P17 gene
1700011F03Rik	RIKEN cDNA 1700011F03 gene
1700013F07Rik	RIKEN cDNA 1700013F07 gene
1700014D04Rik	RIKEN cDNA 1700014D04 gene
1700018L24Rik	RIKEN cDNA 1700018L24 gene
1700019B03Rik	RIKEN cDNA 1700019B03 gene
1700019D03Rik	RIKEN cDNA 1700019D03 gene
1700019L03Rik	RIKEN cDNA 1700019L03 gene
1700019N19Rik	RIKEN cDNA 1700019N19 gene
1700019O17Rik	RIKEN cDNA 1700019O17 gene
1700020K04Rik	RIKEN cDNA 1700020K04 gene
1700021F05Rik	RIKEN cDNA 1700021F05 gene
1700022A21Rik	RIKEN cDNA 1700022A21 gene
1700023D08Rik	RIKEN cDNA 1700023D08 gene
1700023E05Rik	RIKEN cDNA 1700023E05 gene
1700024P04Rik	RIKEN cDNA 1700024P04 gene
1700026D08Rik	RIKEN cDNA 1700026D08 gene
1700026L06Rik	RIKEN cDNA 1700026L06 gene
1700027A15Rik	RIKEN cDNA 1700027A15 gene
1700027D21Rik	RIKEN cDNA 1700027D21 gene
1700034I23Rik	RIKEN cDNA 1700034I23 gene
1700049K14Rik	RIKEN cDNA 1700049K14 gene

Combined list of proteins	
**Protein Symbol**	**Protein Name**
1700055M20Rik	testis, prostate and placenta expressed
1700058C13Rik	RIKEN cDNA 1700058C13 gene
1700061G19Rik	RIKEN cDNA 1700061G19 gene
1700065I17Rik	RIKEN cDNA 1700065I17 gene
1700071K01Rik	RIKEN cDNA 1700071K01 gene
1700080O16Rik	RIKEN cDNA 1700080O16 gene
1700081D17Rik	phosphatidylethanolamine binding protein 4
1700101E01Rik	RIKEN cDNA 1700101E01 gene
1700110M21Rik	RIKEN cDNA 1700110M21 gene
1700112C13Rik	RIKEN cDNA 1700112C13 gene
1700113O17Rik	RIKEN cDNA 1700113O17 gene
2810002D13Rik	leucine rich repeat containing 57
2810408A11Rik	RIKEN cDNA 2810408A11 gene
4732415M23Rik	RIKEN cDNA 4732415M23 gene
4921507P07Rik	RIKEN cDNA 4921507P07 gene
4921517D21Rik	RIKEN cDNA 4921517D21 gene
4921523A10Rik	RIKEN cDNA 4921523A10 gene
4921524E03Rik	aldolase 1, A isoform, retrogene 1
4921530D09Rik	RIKEN cDNA 4921530D09 gene
4930404N11Rik	RIKEN cDNA 4930404N11 gene
4930408G06Rik	RIKEN cDNA 4930408G06 gene
4930415O20Rik	RIKEN cDNA 4930415O20 gene
4930435E12Rik	RIKEN cDNA 4930435E12 gene
4930443G12Rik	RIKEN cDNA 4930443G12 gene
4930511I11Rik	RIKEN cDNA 4930511I11 gene
4930519F16Rik	RIKEN cDNA 4930519F16 gene
4930522H14Rik	RIKEN cDNA 4930522H14 gene
4930523C11Rik	a disintegrin and metallopeptidase domain 6B
4930550C14Rik	RIKEN cDNA 4930550C14 gene
4930555I21Rik	RIKEN cDNA 4930555I21 gene
4930579C15Rik	RIKEN cDNA 4930579C15 gene
4930579J09Rik	RIKEN cDNA 4930579J09 gene
4931408C20Rik	RIKEN cDNA 4931408C20 gene
4931423N10Rik	RIKEN cDNA 4931423N10 gene

Combined list of proteins	
**Protein Symbol**	**Protein Name**
4931432M23Rik	RIKEN cDNA 4931432M23 gene
4932425I24Rik	RIKEN cDNA 4932425I24 gene
4932441B19Rik	RIKEN cDNA 4932441B19 gene
4933400C05Rik	RIKEN cDNA 4933400C05 gene
4933405O20Rik	RIKEN cDNA 4933405O20 gene
4933411K16Rik	RIKEN cDNA 4933411K16 gene
5730437N04Rik	RIKEN cDNA 5730437N04 gene
5730469M10Rik	RIKEN cDNA 5730469M10 gene
9030607L17Rik	RIKEN cDNA 9030607L17 gene
9130227C08Rik	RIKEN cDNA 9130227C08Rik gene
9230002F21Rik	defensin beta 22
AA467197	expressed sequence AA467197
Abca13	ATP-binding cassette, sub-family A (ABC1), member 13
Abcb10	ATP-binding cassette, sub-family B (MDR/TAP), member 10
Abcc12	ATP-binding cassette, sub-family C (CFTR/MRP), member 12
Abcg2	ATP-binding cassette, sub-family G (WHITE), member 2
Abhd11	abhydrolase domain containing 11
Acad9	acyl-Coenzyme A dehydrogenase family, member 9
Acadvl	acyl-Coenzyme A dehydrogenase, very long chain
Acat1	acetyl-Coenzyme A acetyltransferase 1
Ace	angiotensin I converting enzyme (peptidyl-dipeptidase A) 1
Ace3	angiotensin I converting enzyme (peptidyl-dipeptidase A) 3
Aco2	aconitase 2, mitochondrial
Acot10	acyl-CoA thioesterase 10
Acot7	acyl-CoA thioesterase 7
Acot9	acyl-CoA thioesterase 9
Acpp	acid phosphatase, prostate
Acr	acrosin prepropeptide
Acrbp	proacrosin binding protein
Acrbp	proacrosin binding protein
Acrv1	acrosomal vesicle protein 1
Acsbg2	acyl-CoA synthetase bubblegum family member 2
Acsl1	acyl-CoA synthetase long-chain family member 1
Acta1	actin, alpha 1, skeletal muscle

Combined list of proteins	
**Protein Symbol**	**Protein Name**
Actb	actin, beta
Actc1	actin, alpha, cardiac muscle 1
Actg1	actin, gamma, cytoplasmic 1
Actg2	actin, gamma 2, smooth muscle, enteric
Actl7a	actin-like 7a
Actn4	actinin alpha 4
Actrt1	actin-related protein T1
Actrt2	actin-related protein T2
Acyp1	acylphosphatase 1, erythrocyte (common) type
Adad1	adenosine deaminase domain containing 1 (testis specific)
Adam15	a disintegrin and metallopeptidase domain 15 (metargidin)
Adam17	a disintegrin and metallopeptidase domain 17
Adam18	a disintegrin and metallopeptidase domain 18
Adam1a	a disintegrin and metallopeptidase domain 1a
Adam1b	a disintegrin and metallopeptidase domain 1b
Adam1b	a disintegrin and metallopeptidase domain 1b
Adam2	a disintegrin and metallopeptidase domain 2
Adam21	a disintegrin and metallopeptidase domain 21
Adam23	a disintegrin and metallopeptidase domain 23
Adam24	a disintegrin and metallopeptidase domain 24 (testase 1)
Adam25	a disintegrin and metallopeptidase domain 25 (testase 2)
Adam26a	a disintegrin and metallopeptidase domain 26A (testase 3)
Adam28	a disintegrin and metallopeptidase domain 28
Adam29	a disintegrin and metallopeptidase domain 29
Adam3	a disintegrin and metallopeptidase domain 3 (cyritestin)
Adam32	a disintegrin and metallopeptidase domain 32
Adam4	a disintegrin and metallopeptidase domain 4
Adam5	a disintegrin and metallopeptidase domain 5
Adam6	a disintegrin and metallopeptidase domain 6A
Adam7	a disintegrin and metallopeptidase domain 7
Adcy10	adenylate cyclase 10
Adcy3	adenylate cyclase 3
Adcy8	adenylate cyclase 8

Combined list of proteins	
**Protein Symbol**	**Protein Name**
Adh1	alcohol dehydrogenase 1 (class I)
Agfg1	ArfGAP with FG repeats 1
Agfg1	ArfGAP with FG repeats 1
Agt	angiotensinogen (serpin peptidase inhibitor, clade A, member 8)
Agtpbp1	ATP/GTP binding protein 1
Ahcy	S-adenosylhomocysteine hydrolase
Aim1l	absent in melanoma 1-like
Ak1	adenylate kinase 1
Ak2	adenylate kinase 2
Ak7	adenylate kinase 7
Akap1	A kinase (PRKA) anchor protein 1
Akap10	A kinase (PRKA) anchor protein 10
Akap11	A kinase (PRKA) anchor protein 11
Akap12	A kinase (PRKA) anchor protein (gravin) 12
Akap13	A kinase (PRKA) anchor protein 13
Akap3	A kinase (PRKA) anchor protein 3
Akap4	A kinase (PRKA) anchor protein 4
Akap8	A kinase (PRKA) anchor protein 8
Akr1a4	aldo-keto reductase family 1, member A4 (aldehyde reductase)
Akr1b10	aldo-keto reductase family 1, member B10 (aldose reductase)
Akr1b3	aldo-keto reductase family 1, member B3 (aldose reductase)
Akr1b7	aldo-keto reductase family 1, member B7
Akr7a5	aldo-keto reductase family 7, member A5 (aflatoxin aldehyde reductase)
Alad	aminolevulinate, delta-, dehydratase
Alb	albumin
Aldh1a1	aldehyde dehydrogenase family 1, subfamily A1
Aldh1a2	aldehyde dehydrogenase family 1, subfamily A2
Aldh9a1	aldehyde dehydrogenase 9, subfamily A1
Aldoa	aldolase A, fructose-bisphosphate
Aldoart2	aldolase 1, A isoform, retrogene 2
Aldoc	aldolase C, fructose-bisphosphate
Alms1	Alstrom syndrome 1 homolog (human)
Als2cr11	amyotrophic lateral sclerosis 2 (juvenile) chromosome region, candidate 11 (human)
Amdhd2	amidohydrolase domain containing 2

Combined list of proteins	
**Protein Symbol**	**Protein Name**
Anapc5	anaphase-promoting complex subunit 5
Ank1	ankyrin 1, erythroid
Ankrd11	ankyrin repeat domain 11
Ankrd5	ankyrin repeat domain 5
Ankrd57	ankyrin repeat domain 57
Anxa2	annexin A2
Anxa4	annexin A4
Anxa5	annexin A5
Anxa6	annexin A6
Ap3d1	adaptor-related protein complex 3, delta 1 subunit
Apbb2	amyloid beta (A4) precursor protein-binding, family B, member 2
Apoa1	apolipoprotein A-I
Apoa1bp	apolipoprotein A-I binding protein
Apoa2	apolipoprotein A-II
Apoa4	apolipoprotein A-IV
Apof	apolipoprotein F
Apoo	apolipoprotein O
Apool	apolipoprotein O-like
Aqp7	aquaporin 7
Arf2	ADP-ribosylation factor 2
Arf6	ADP-ribosylation factor 6
Arhgap17	Rho GTPase activating protein 17
Arhgdia	Rho GDP dissociation inhibitor (GDI) alpha
Arhgef1	Rho guanine nucleotide exchange factor (GEF) 1
Armc3	armadillo repeat containing 3
Arpm1	actin related protein M1
Arsa	arylsulfatase A
Art3	ADP-ribosyltransferase 3
Asb9	ankyrin repeat and SOCS box-containing 9
Asrgl1	asparaginase like 1
Atg16l1	autophagy-related 16-like 1 (yeast)
Atm	ataxia telangiectasia mutated homolog (human)
Atn1	atrophin 1

Combined list of proteins	
**Protein Symbol**	**Protein Name**
Atox1	ATX1 (antioxidant protein 1) homolog 1 (yeast)
Atp13a1	ATPase type 13A1
Atp1a1	ATPase, Na+/K+ transporting, alpha 1 polypeptide
Atp1a2	ATPase, Na+/K+ transporting, alpha 2 polypeptide
Atp1a3	ATPase, Na+/K+ transporting, alpha 3 polypeptide
Atp1a4	ATPase, Na+/K+ transporting, alpha 4 polypeptide
Atp1b3	ATPase, Na+/K+ transporting, beta 3 polypeptide
Atp2b2	ATPase, Ca++ transporting, plasma membrane 2
Atp2b4	ATPase, Ca++ transporting, plasma membrane 4
Atp2b4	ATPase, Ca++ transporting, plasma membrane 4
Atp5a1	ATP synthase, H+ transporting, mitochondrial F1 complex, alpha subunit, isoform 1
Atp5b	ATP synthase, H+ transporting mitochondrial F1 complex, beta subunit
Atp5c1	ATP synthase, H+ transporting, mitochondrial F1 complex, gamma polypeptide 1
Atp5d	ATP synthase, H+ transporting, mitochondrial F1 complex, delta subunit
Atp5f1	ATP synthase, H+ transporting, mitochondrial F0 complex, subunit b, isoform 1
Atp5h	ATP synthase, H+ transporting, mitochondrial F0 complex, subunit d
Atp5k	ATP synthase, H+ transporting, mitochondrial F1F0 complex, subunit e
Atp5l	ATP synthase, H+ transporting, mitochondrial F0 complex, subunit g
Atp5o	ATP synthase, H+ transporting, mitochondrial F1 complex, O subunit
Atp5s	ATP synthase, H+ transporting, mitochondrial F0 complex, subunit s
Atp6v1a	ATPase, H+ transporting, lysosomal V1 subunit A
Atp6v1b2	ATPase, H+ transporting, lysosomal V1 subunit B2
Atp6v1e1	ATPase, H+ transporting, lysosomal V1 subunit E1
Atp6v1h	ATPase, H+ transporting, lysosomal V1 subunit H
Atp8b3	ATPase, class I, type 8B, member 3
B2m	beta-2 microglobulin
B4galt1	UDP-Gal:betaGlcNAc beta 1,4- galactosyltransferase, polypeptide 1
B4galt4	UDP-Gal:betaGlcNAc beta 1,4-galactosyltransferase, polypeptide 4
Bag6	BCL2-associated athanogene 6
Basp1	brain abundant, membrane attached signal protein 1
Bat3	HLA-B-associated transcript 3
Bax	BCL2-associated X protein
BB014433	expressed sequence BB014433
Bbs2	Bardet-Biedl syndrome 2 (human)

Combined list of proteins	
**Protein Symbol**	**Protein Name**
Bbs4	Bardet-Biedl syndrome 4 (human)
BC005764	cDNA sequence BC005764
BC049635	cDNA sequence BC049635
BC051142	cDNA sequence BC051142
BC089491	cDNA sequence BC089491
BC100451	cDNA sequence BC100451
Bcs1l	BCS1-like (yeast)
Bdh1	3-hydroxybutyrate dehydrogenase, type 1
Birc6	baculoviral IAP repeat-containing 6
Blvra	biliverdin reductase A
Boll	bol, boule-like (Drosophila)
Bpgm	2,3-bisphosphoglycerate mutase
Bpnt1	bisphosphate 3'-nucleotidase 1
Brp44	brain protein 44
Brs3	bombesin-like receptor 3
Bsg	basigin
Bspry	B-box and SPRY domain containing
Bzrap1	benzodiazapine receptor associated protein 1
C130090K23Rik	cell wall biogenesis 43 C-terminal homolog (S. cerevisiae)
C1qbp	complement component 1, q subcomponent binding protein
C3	complement component 3
C77370	expressed sequence C77370
C920008N22Rik	RIKEN cDNA C920008N22 gene
Cabyr	calcium-binding tyrosine-(Y)-phosphorylation regulated (fibrousheathin 2)
Cacng3	calcium channel, voltage-dependent, gamma subunit 3
Cadm1	cell adhesion molecule 1
Calca	calcitonin/calcitonin-related polypeptide, alpha
Calcb	calcitonin-related polypeptide, beta
Calm1	calmodulin 1
Calm2	calmodulin 2
Calm3	calmodulin 3
Calr	calreticulin
Camk2b	calcium/calmodulin-dependent protein kinase II, beta
Camk2d	calcium/calmodulin-dependent protein kinase II, delta

Combined list of proteins	
**Protein Symbol**	**Protein Name**
Camk4	calcium/calmodulin-dependent protein kinase IV
Camp	cathelicidin antimicrobial peptide
Cand1	cullin associated and neddylation disassociated 1
Canx	calnexin
Cap1	CAP, adenylate cyclase-associated protein 1 (yeast)
Capza3	capping protein (actin filament) muscle Z-line, alpha 3
Capzb	capping protein (actin filament) muscle Z-line, beta
Car2	carbonic anhydrase 2
Car3	carbonic anhydrase 3
Car4	carbonic anhydrase 4
Casc1	cancer susceptibility candidate 1
Catsper1	cation channel, sperm associated 1
Catsper2	cation channel, sperm associated 2
Catsper3	cation channel, sperm associated 3
Catsper4	cation channel, sperm associated 4
Catsperb	catsper channel auxiliary subunit beta
Catsperd	catsper channel auxiliary subunit delta
Catsperg2	catsper channel auxiliary subunit gamma 2
Cav1	caveolin 1, caveolae protein
Cav2	caveolin 2
Cav3	caveolin 3
Cbr1	carbonyl reductase 1
Cbr4	carbonyl reductase 4
Ccdc105	coiled-coil domain containing 105
Ccdc11	coiled-coil domain containing 11
Ccdc116	coiled-coil domain containing 116
Ccdc135	coiled-coil domain containing 135
Ccdc136	coiled-coil domain containing 136
Ccdc151	coiled-coil domain containing 151
Ccdc19	coiled-coil domain containing 19
Ccdc21	coiled-coil domain containing 21
Ccdc27	coiled-coil domain containing 27
Ccdc40	coiled-coil domain containing 40
Ccdc54	coiled-coil domain containing 54

Combined list of proteins	
**Protein Symbol**	**Protein Name**
Ccdc58	coiled-coil domain containing 58
Ccdc63	coiled-coil domain containing 63
Ccdc81	coiled-coil domain containing 81
Ccdc88c	coiled-coil domain containing 88C
Ccin	calicin
Ccna1	cyclin A1
Ccnd1	cyclin D1
Cct2	chaperonin containing Tcp1, subunit 2 (beta)
Cct3	chaperonin containing Tcp1, subunit 3 (gamma)
Cct4	chaperonin containing Tcp1, subunit 4 (delta)
Cct5	chaperonin containing Tcp1, subunit 5 (epsilon)
Cct6a	chaperonin containing Tcp1, subunit 6a (zeta)
Cct7	chaperonin containing Tcp1, subunit 7 (eta)
Cct8	chaperonin containing Tcp1, subunit 8 (theta)
Cd109	CD109 antigen
Cd151	CD151 antigen
Cd46	CD46 antigen, complement regulatory protein
Cd52	CD52 antigen
Cd55	CD55 antigen
Cd59a	CD59a antigen
Cd59b	CD59b antigen
Cdc34	cell division cycle 34 homolog (S. cerevisiae)
Cdk2	cyclin-dependent kinase 2
Ceacam10	carcinoembryonic antigen-related cell adhesion molecule 10
Ceacam2	carcinoembryonic antigen-related cell adhesion molecule 2
Cecr5	cat eye syndrome chromosome region, candidate 5 homolog (human)
Celf3	CUGBP, Elav-like family member 3
Cenpe	centromere protein E
Cep152	centrosomal protein 152
Cep350	centrosomal protein 350
Ces3	carboxylesterase 3
Ces7	carboxylesterase 7
Cfh	complement component factor h
Cfl1	cofilin 1, non-muscle

Combined list of proteins	
**Protein Symbol**	**Protein Name**
Chchd6	coiled-coil-helix-coiled-coil-helix domain containing 6
Chdh	choline dehydrogenase
Chit1	chitinase 1 (chitotriosidase)
Chrna7	cholinergic receptor, nicotinic, alpha polypeptide 7
Cisd1	CDGSH iron sulfur domain 1
Ckb	creatine kinase, brain
Ckm	creatine kinase, muscle
Clca1	chloride channel calcium activated 1
Clca2	chloride channel calcium activated 2
Clgn	calmegin
Clic1	chloride intracellular channel 1
Clmn	calmin
Cltc	clathrin, heavy polypeptide (Hc)
Clu	clusterin
Cmpk1	cytidine monophosphate (UMP-CMP) kinase 1
Cnga3	cyclic nucleotide gated channel alpha 3
Cngb1	cyclic nucleotide gated channel beta 1
Cnn1	calponin 1
Coil	coilin
Col8a2	collagen, type VIII, alpha 2
Commd10	COMM domain containing 10
Copb1	coatomer protein complex, subunit beta 1
Cotl1	coactosin-like 1 (Dictyostelium)
Cox4i1	cytochrome c oxidase subunit IV isoform 1
Cox5a	cytochrome c oxidase, subunit Va
Cox6b2	cytochrome c oxidase subunit VIb polypeptide 2
Cox6c	cytochrome c oxidase, subunit VIc
Cox7a2	cytochrome c oxidase, subunit VIIa 2
Cox7a2l	cytochrome c oxidase subunit VIIa polypeptide 2-like
Cp	ceruloplasmin
Cpa5	carboxypeptidase A5
Cpeb1	cytoplasmic polyadenylation element binding protein 1
Cpt1b	carnitine palmitoyltransferase 1b, muscle
Cpt2	carnitine palmitoyltransferase 2

Combined list of proteins	
**Protein Symbol**	**Protein Name**
Cpvl	carboxypeptidase, vitellogenic-like
Crat	carnitine acetyltransferase
Creb3	cAMP responsive element binding protein 3
Creb3l4	cAMP responsive element binding protein 3-like 4
Crisp1	cysteine-rich secretory protein 1
Crisp1	cysteine-rich secretory protein 1
Crisp2	cysteine-rich secretory protein 2
Crisp3	cysteine-rich secretory protein 3
Crisp4	cysteine-rich secretory protein 4
Cryl1	crystallin, lambda 1
Cryzl1	crystallin, zeta (quinone reductase)-like 1
Cs	citrate synthase
Csk	c-src tyrosine kinase
Csl	citrate synthase like
Csnk1a1	casein kinase 1, alpha 1
Csnk2a2	casein kinase 2, alpha prime polypeptide
Csnk2b	casein kinase 2, beta polypeptide
Csrp1	cysteine and glycine-rich protein 1
Cst13	cystatin 13
Cst8	cystatin 8 (cystatin-related epididymal spermatogenic)
Cstf2	cleavage stimulation factor, 3' pre-RNA subunit 2
Ctsd	cathepsin D
Ctsl	cathepsin L
Cttn	cortactin
Cubn	cubilin (intrinsic factor-cobalamin receptor)
Cul3	cullin 3
Cutc	cutC copper transporter homolog (E.coli)
Cuzd1	CUB and zona pellucida-like domains 1
Cwf19l1	CWF19-like 1, cell cycle control (S. pombe)
Cyb5	cytochrome b-5
Cyb5b	cytochrome b5 type B
Cyc1	cytochrome c-1
Cycs	cytochrome c, somatic
Cyct	cytochrome c, testis

Combined list of proteins	
**Protein Symbol**	**Protein Name**
Cylc1	cylicin, basic protein of sperm head cytoskeleton 1
Cylc2	cylicin, basic protein of sperm head cytoskeleton 2
D10Jhu81e	DNA segment, Chr 10, Johns Hopkins University 81 expressed
D11Wsu47e	DNA segment, Chr 11, Wayne State University 47, expressed
D230040A04Rik	RIKEN cDNA D230040A04 gene
D3Bwg0562e	DNA segment, Chr 3, Brigham & Women's Genetics 0562 expressed
Dak	dihydroxyacetone kinase 2 homolog (yeast)
Dazap1	DAZ associated protein 1
Dazl	deleted in azoospermia-like
Dbi	diazepam binding inhibitor
Dbil5	diazepam binding inhibitor-like 5
Ddt	D-dopachrome tautomerase
Ddx25	DEAD (Asp-Glu-Ala-Asp) box polypeptide 25
Ddx4	DEAD (Asp-Glu-Ala-Asp) box polypeptide 4
Defb19	defensin beta 19
Defb22	defensin beta 22
Dffa	DNA fragmentation factor, alpha subunit
Dhrs1	dehydrogenase/reductase (SDR family) member 1
Dhrs4	dehydrogenase/reductase (SDR family) member 4
Dhrs7b	dehydrogenase/reductase (SDR family) member 7B
Dhx29	DEAH (Asp-Glu-Ala-His) box polypeptide 29
Disc1	disrupted in schizophrenia 1
Dkkl1	dickkopf-like 1
Dlat	dihydrolipoamide S-acetyltransferase (E2 component of pyruvate dehydrogenase complex)
Dld	dihydrolipoamide dehydrogenase
Dlst	dihydrolipoamide S-succinyltransferase (E2 component of 2-oxo-glutarate complex)
Dmc1	DMC1 dosage suppressor of mck1 homolog, meiosis-specific homologous recombination (yeast)
Dmxl2	Dmx-like 2
Dnaaf1	dynein, axonemal assembly factor 1
Dnahc1	dynein, axonemal, heavy chain 1
Dnahc1	dynein, axonemal, heavy chain 1
Dnahc10	dynein, axonemal, heavy chain 10
Dnahc12	dynein, axonemal, heavy chain 12
Dnahc17	dynein, axonemal, heavy chain 17

Combined list of proteins	
**Protein Symbol**	**Protein Name**
Dnahc17	dynein, axonemal, heavy chain 17
Dnahc2	dynein, axonemal, heavy chain 2
Dnahc2	dynein, axonemal, heavy chain 2
Dnahc3	dynein, axonemal, heavy chain 3
Dnahc3	dynein, axonemal, heavy chain 3
Dnahc5	dynein, axonemal, heavy chain 5
Dnahc6	dynein, axonemal, heavy chain 6
Dnahc7a	dynein, axonemal, heavy chain 7A
Dnahc7a	dynein, axonemal, heavy chain 7A
Dnahc7b	dynein, axonemal, heavy chain 7B
Dnahc7l	dynein, axonemal, heavy chain 12
Dnahc8	dynein, axonemal, heavy chain 8
Dnahc9	dynein, axonemal, heavy chain 9
Dnaic1	dynein, axonemal, intermediate chain 1
Dnaic2	dynein, axonemal, intermediate chain 2
Dnaja1	DnaJ (Hsp40) homolog, subfamily A, member 1
Dnaja4	DnaJ (Hsp40) homolog, subfamily A, member 4
Dnajb11	DnaJ (Hsp40) homolog, subfamily B, member 11
Dnajb13	DnaJ (Hsp40) related, subfamily B, member 13
Dnajb3	DnaJ (Hsp40) homolog, subfamily B, member 3
Dnajb6	DnaJ (Hsp40) homolog, subfamily B, member 6
Dnajc11	DnaJ (Hsp40) homolog, subfamily C, member 11
Dnali1	dynein, axonemal, light intermediate polypeptide 1
Dnmt3l	DNA (cytosine-5-)-methyltransferase 3-like
Dnpep	aspartyl aminopeptidase
Dock1	dedicator of cytokinesis 1
Dock11	dedicator of cytokinesis 11
Dpcd	deleted in primary ciliary dyskinesia
Dpep3	dipeptidase 3
Dpp3	dipeptidylpeptidase 3
Drd2	dopamine receptor 2
Dsg1c	desmoglein 1 gamma
Dstn	destrin
Dynll1	dynein light chain LC8-type 1

Combined list of proteins	
**Protein Symbol**	**Protein Name**
Dynlt1	dynein light chain Tctex-type 1D
Dynlt1b	dynein light chain Tctex-type 1B
E130112L23Rik	RIKEN cDNA E130112L23 gene
E430028B21Rik	phosphodiesterase 12
Eef1a1	eukaryotic translation elongation factor 1 alpha 1
Eef1d	eukaryotic translation elongation factor 1 delta (guanine nucleotide exchange protein)
Eef1g	eukaryotic translation elongation factor 1 gamma
Efcab3	EF-hand calcium binding domain 3
Efhb	EF hand domain family, member B
Efhc1	EF-hand domain (C-terminal) containing 1
Efhc2	EF-hand domain (C-terminal) containing 2
EG433182	predicted gene 5506
Egfl7	EGF-like domain 7
Eif2ak4	eukaryotic translation initiation factor 2 alpha kinase 4
Eif4g3	eukaryotic translation initiation factor 4 gamma, 3
Elmo3	engulfment and cell motility 3, ced-12 homolog (C. elegans)
Enah	enabled homolog (Drosophila)
Enkur	enkurin, TRPC channel interacting protein
Eno1	enolase 1, alpha non-neuron
Eno2	enolase 2, gamma neuronal
Eno3	enolase 3, beta muscle
Enpp5	ectonucleotide pyrophosphatase/phosphodiesterase 5
Eprs	glutamyl-prolyl-tRNA synthetase
Eqtn	equatorin, sperm acrosome associated
Ercc6	excision repair cross-complementing rodent repair deficiency, complementation group 6
Ergic1	endoplasmic reticulum-golgi intermediate compartment (ERGIC) 1
Erlin2	ER lipid raft associated 2
Es1	esterase 1
Esx1	extraembryonic, spermatogenesis, homeobox 1
Etfa	electron transferring flavoprotein, alpha polypeptide
Etfdh	electron transferring flavoprotein, dehydrogenase
Etv5	ets variant gene 5

Combined list of proteins	
**Protein Symbol**	**Protein Name**
Fabp4	fatty acid binding protein 4, adipocyte
Fabp9	fatty acid binding protein 9, testis
Fahd2a	fumarylacetoacetate hydrolase domain containing 2A
Fam154a	family with sequence similarity 154, member A
Fam166a	family with sequence similarity 166, member A
Fam170b	family with sequence similarity 170, member B
Fam178a	family with sequence similarity 178, member A
Fam65c	family with sequence similarity 65, member C
Fam71a	family with sequence similarity 71, member A
Fam71b	family with sequence similarity 71, member B
Fam78a	family with sequence similarity 78, member A
Fancl	Fanconi anemia, complementation group L
Fank1	fibronectin type 3 and ankyrin repeat domains 1
Fbn1	fibrillin 1
Fbp1	fructose bisphosphatase 1
Fdx1l	ferredoxin 1-like
Fer	fer (fms/fps related) protein kinase
Fgd1	FYVE, RhoGEF and PH domain containing 1
Fgfr1	fibroblast growth factor receptor 1
Fh1	fumarate hydratase 1
fh15	
Fhl4	four and a half LIM domains 4
Fis1	fission 1 (mitochondrial outer membrane) homolog (yeast)
Fkbp10	FK506 binding protein 10
Fkbp6	FK506 binding protein 6
Fn1	fibronectin 1
Fndc3a	fibronectin type III domain containing 3A
Fndc8	fibronectin type III domain containing 8
Foxo3	forkhead box O3
Frmd4a	FERM domain containing 4A
Fscb	fibrous sheath CABYR binding protein
Fscn1	fascin homolog 1, actin bundling protein (Strongylocentrotus purpuratus)
Fscn3	fascin homolog 3, actin-bundling protein, testicular (Strongylocentrotus purpuratus)
Fsip2	fibrous sheath-interacting protein 2

Combined list of proteins	
**Protein Symbol**	**Protein Name**
Ftmt	ferritin mitochondrial
Fxn	frataxin
Fyn	Fyn proto-oncogene
G6pd2	glucose-6-phosphate dehydrogenase 2
Gadd45a	growth arrest and DNA-damage-inducible 45 alpha
Galnt3	UDP-N-acetyl-alpha-D-galactosamine:polypeptide N-acetylgalactosaminyltransferase 3
Ganab	alpha glucosidase 2 alpha neutral subunit
Gapdh	glyceraldehyde-3-phosphate dehydrogenase
Gapdhs	glyceraldehyde-3-phosphate dehydrogenase, spermatogenic
Gas8	growth arrest specific 8
Gc	group specific component
Gcc2	GRIP and coiled-coil domain containing 2
Gdi2	guanosine diphosphate (GDP) dissociation inhibitor 2
Gfap	glial fibrillary acidic protein
Ggn	gametogenetin
Gk2	glycerol kinase 2
Glb1l	galactosidase, beta 1-like
Glcci1	glucocorticoid induced transcript 1
glg1	golgi apparatus protein 1
Glipr1l1	GLI pathogenesis-related 1 like 1
Glipr1l2	GLI pathogenesis-related 1 like 2
Glra1	glycine receptor, alpha 1 subunit
Glra2	glycine receptor, alpha 2 subunit
Glrb	glycine receptor, beta subunit
Glud1	glutamate dehydrogenase 1
Glul	glutamate-ammonia ligase (glutamine synthetase)
Gm128	predicted gene 128
Gm1281	
Gm13334	predicted gene 13334
Gm136	predicted gene 136
Gm166	predicted gene 166
Gm1673	predicted gene 1673
Gm3336	predicted gene 3336
Gm4535	predicted gene 4535

Combined list of proteins	
**Protein Symbol**	**Protein Name**
Gm46	predicted gene 46
Gm4764	predicted gene 4764
Gm5803	predicted gene 5803
Gm595	predicted gene 595
Gm6316	predicted gene 6316
Gm6413	predicted gene 6413
Gm6981	predicted gene 6981
Gm8394	predicted gene 8394
Gm884	predicted gene 884
Gm9047	predicted gene 9047
Gm9832	predicted gene 9832
Gnpda1	glucosamine-6-phosphate deaminase 1
Gnpda2	glucosamine-6-phosphate deaminase 2
Gopc	golgi associated PDZ and coiled-coil motif containing
Gpd1	glycerol-3-phosphate dehydrogenase 1 (soluble)
gpd2	glycerol phosphate dehydrogenase 2, mitochondrial
Gpi1	glucose phosphate isomerase 1
Gpx3	glutathione peroxidase 3
Gpx4	glutathione peroxidase 4
Gpx5	glutathione peroxidase 5
Gsg1	germ cell-specific gene 1
Gsk3b	glycogen synthase kinase 3 beta
Gsn	gelsolin
Gstm1	glutathione S-transferase, mu 1
Gstm2	glutathione S-transferase, mu 2
Gstm5	glutathione S-transferase, mu 5
Gsto1	glutathione S-transferase omega 1
Gsto2	glutathione S-transferase omega 2
Gstp1	glycerol kinase-like 1
Gstt2	glutathione S-transferase, theta 2
Gstt3	glutathione S-transferase, theta 3
Gtl3	gene trap locus 3
Gusb	glucuronidase, beta
Gyk	glycerol kinase

Combined list of proteins	
**Protein Symbol**	**Protein Name**
Gykl1	glycerol kinase-like 1
H1fnt	H1 histone family, member N, testis-specific
H2afx	H2A histone family, member X
H2-Ke6	H2-K region expressed gene 6
Hadha	hydroxyacyl-Coenzyme A dehydrogenase/3-ketoacyl-Coenzyme A thiolase/enoyl-Coenzyme A hydratase (trifunctional protein), alpha subunit
Hadhb	hydroxyacyl-Coenzyme A dehydrogenase/3-ketoacyl-Coenzyme A thiolase/enoyl-Coenzyme A hydratase (trifunctional protein), beta subunit
Hba-a1	hemoglobin alpha, adult chain 1
Hba-a2	hemoglobin, beta adult major chain
Hbb-b1	hemoglobin, beta adult major chain
Hbb-b2	hexokinase 1
Hc	hemolytic complement
Hc	hemolytic complement
Hdac1	histone deacetylase 1
Hdhd1a	haloacid dehalogenase-like hydrolase domain containing 1A
Hdhd3	haloacid dehalogenase-like hydrolase domain containing 3
Heatr7a	HEAT repeat containing 7A
Heatr7b2	XVHEAT repeat family member 7B2
Hibadh	3-hydroxyisobutyrate dehydrogenase
Hibch	3-hydroxyisobutyryl-Coenzyme A hydrolase
Hils1	histone H1-like protein in spermatids 1
Hint1	histidine triad nucleotide binding protein 1
Hira	histone cell cycle regulation defective homolog A (S. cerevisiae)
Hist1h1t	histone cluster 1, H1t
Hist1h2ba	histone cluster 1, H2ba
Hist1h4i	histone cluster 1, H4i
Hk1	hexokinase 1
Hk2	hexokinase 2
Hkdc1	hexokinase domain containing 1
Hmox2	heme oxygenase (decycling) 2
Hnrnpa1	heterogeneous nuclear ribonucleoprotein A1
Hnrnpu	heterogeneous nuclear ribonucleoprotein U
Hnrpa2b1	hypoxanthine guanine phosphoribosyl transferase 1

Combined list of proteins	
**Protein Symbol**	**Protein Name**
Hprt1	hemopexin
Hpx	heat shock protein 90 alpha (cytosolic), class B member 1
Hsd17b10	hydroxysteroid (17-beta) dehydrogenase 10
Hsp90aa1	heat shock protein 90, alpha (cytosolic), class A member 1
Hsp90ab1	heat shock protein 90 alpha (cytosolic), class B member 1
Hsp90b1	heat shock protein 90, beta (Grp94), member 1
Hsp90b1	heat shock protein 90, beta (Grp94), member 1
Hspa1b	heat shock protein 5
Hspa1l	heat shock protein 1-like
Hspa1l	heat shock protein 1-like
Hspa2	heat shock protein 2
Hspa4l	heat shock protein 4 like
Hspa5	heat shock protein 5
Hspa8	heat shock protein 8
Hspa9	heat shock protein 9
Hspb9	heat shock protein, alpha-crystallin-related, B9
Hspbp1	HSPA (heat shock 70kDa) binding protein, cytoplasmic cochaperone 1
Hspd1	heat shock protein 1 (chaperonin)
Htra2	HtrA serine peptidase 2
Htt	huntingtin
Hyal3	hyaluronoglucosaminidase 3
Hyal5	hyaluronoglucosaminidase 5
Hyal6	hyaluronoglucosaminidase 6
Hydin	hydrocephalus inducing
Hyou1	hypoxia up-regulated 1
Idh1	isocitrate dehydrogenase 1 (NADP+), soluble
Idh3a	isocitrate dehydrogenase 3 (NAD+) alpha
Idh3b	isocitrate dehydrogenase 3 (NAD+) beta
Ier3	immediate early response 3
Ift88	intraflagellar transport 88
Igbp1b	immunoglobulin (CD79A) binding protein 1b
Ighg1	keratin 1
Igsf8	immunoglobulin superfamily, member 8
Il4i1	interleukin 4 induced 1

Combined list of proteins	
**Protein Symbol**	**Protein Name**
Il5	interleukin 5
Immt	inner membrane protein, mitochondrial
Impa1	inositol (myo)-1(or 4)-monophosphatase 1
Ing4	inhibitor of growth family, member 4
Inpp5a	inositol polyphosphate-5-phosphatase A
Inpp5b	inositol polyphosphate-5-phosphatase B
Insl6	insulin-like 6
Iqcd	IQ motif containing D
Iqcf5	IQ motif containing F5
Irgc1	immunity-related GTPase family, cinema 1
Isoc2a	isochorismatase domain containing 2a
Isyna1	myo-inositol 1-phosphate synthase A1
Itga3	integrin alpha 3
Itga5	integrin alpha 5 (fibronectin receptor alpha)
Itga6	integrin alpha 6
Itgb1	integrin beta 1 (fibronectin receptor beta)
Itgb2	integrin beta 2
Itpr1	inositol 1,4,5-trisphosphate receptor 1
Itpr2	inositol 1,4,5-triphosphate receptor 2
Izumo1	izumo sperm-egg fusion 1
Kcnk4	potassium channel, subfamily K, member 4
Kcnu1	potassium channel, subfamily U, member 1
Kdm3a	lysine (K)-specific demethylase 3A
Kdm5b	lysine (K)-specific demethylase 5B
Khdrbs1	KH domain containing, RNA binding, signal transduction associated 1
Kif24	kinesin family member 24
Kif9	kinesin family member 9
Kit	kit oncogene
Klhl10	kelch-like 10 (Drosophila)
Klk1b27	kallikrein 1-related peptidase b27
Klkb1	kallikrein B, plasma 1
Kndc1	kinase non-catalytic C-lobe domain (KIND) containing 1
Krt1	keratin 1
Krt10	keratin 10

Combined list of proteins	
**Protein Symbol**	**Protein Name**
Krt19	keratin 19
Krt2	keratin 2
Krt24	keratin 24
Krt79	keratin 79
Krt8	keratin 8
Krt81	keratin 81
Krt85	keratin 85
Krt9	keratin 9
L2hgdh	L-2-hydroxyglutarate dehydrogenase
Lamb1	laminin B1
Lamb1-1	laminin B1 subunit 1
Lamb2	laminin, beta 2
Lap3	leucine aminopeptidase 3
Lcn5	lipocalin 5
Ldha	lactate dehydrogenase A-like 6B
Ldhal6b	lactate dehydrogenase C
Ldhb	lactate dehydrogenase B
Ldhc	lactate dehydrogenase C
Letm1	leucine zipper-EF-hand containing transmembrane protein 1
Lipe	lipase, hormone sensitive
Lman2	lectin, mannose-binding 2
Lpp	LIM domain containing preferred translocation partner in lipoma
Lrch3	leucine-rich repeats and calponin homology (CH) domain containing 3
Lrguk	leucine-rich repeats and guanylate kinase domain containing
Lrrc18	leucine rich repeat containing 18
Lrrc24	leucine rich repeat containing 24
Lrrc34	leucine rich repeat containing 34
Lrrc37a	leucine rich repeat containing 37A
Lrrc57	leucine rich repeat containing 57
Lrrc7	leucine rich repeat containing 7
Lta4h	leukotriene A4 hydrolase
Ltb4dh	leukotriene B4 dehydrogenase
Ltf	lactotransferrin

Combined list of proteins	
**Protein Symbol**	**Protein Name**
Lum	lumican
Luzp1	leucine zipper protein 1
Ly6g5b	lymphocyte antigen 6 complex, locus G5B
Lypd4	Ly6/Plaur domain containing 4
Lypla1	lysophospholipase 1
Lyzl1	lysozyme-like 1
Lyzl6	lysozyme-like 6
M6pr	mannose-6-phosphate receptor, cation dependent
Macf1	microtubule-actin crosslinking factor 1
Man2b2	mannosidase 2, alpha B2
Man2c1	mannosidase, alpha, class 2C, member 1
Mast2	microtubule associated serine/threonine kinase 2
Mcts2	malignant T cell amplified sequence 2
Mdh1	mucin 5, subtype B, tracheobronchial
Mdh2	malate dehydrogenase 2, NAD (mitochondrial)
Mdn1	midasin homolog (yeast)
Me1	malic enzyme 1, NADP(+)-dependent, cytosolic
Me2	malic enzyme 2, NAD(+)-dependent, mitochondrial
Mecr	mitochondrial trans-2-enoyl-CoA reductase
Mei1	meiosis defective 1
Meig1	meiosis expressed gene 1
Mfge8	milk fat globule-EGF factor 8 protein
Mfge8	milk fat globule-EGF factor 8 protein
Mgea5	meningioma expressed antigen 5 (hyaluronidase)
Mif4gd	MIF4G domain containing
Mink1	misshapen-like kinase 1 (zebrafish)
Mipol1	mirror-image polydactyly gene 1 homolog (human)
Mkks	McKusick-Kaufman syndrome
Mlh1	mutL homolog 1 (E. coli)
Mlh3	mutL homolog 3 (E coli)
Mll2	myeloid/lymphoid or mixed-lineage leukemia 2
Mmel1	membrane metallo-endopeptidase-like 1
Mmp1a	matrix metallopeptidase 1a (interstitial collagenase)
Mns1	meiosis-specific nuclear structural protein 1

Combined list of proteins	
**Protein Symbol**	**Protein Name**
Mpi	mannose phosphate isomerase
Mrps36	mitochondrial ribosomal protein S36
Ms4a13	membrane-spanning 4-domains, subfamily A, member 13
Ms4a14	membrane-spanning 4-domains, subfamily A, member 14
Msh4	mutS homolog 4 (E. coli)
Msh5	mutS homolog 5 (E. coli)
Msmb	beta-microseminoprotein
Mtap1a	microtubule-associated protein 1 A
Mtch2	mitochondrial carrier homolog 2 (C. elegans)
Mtx2	metaxin 2
Muc5	murinoglobulin 1
Mug1	myosin, heavy polypeptide 10, non-muscle
Mybl2	myeloblastosis oncogene-like 2
Myh10	myosin, heavy polypeptide 11, smooth muscle
Myh11	myosin, heavy polypeptide 9, non-muscle
Myh9	myosin, light polypeptide 6, alkali, smooth muscle and non-muscle
Myl6	myosin, light polypeptide 9, regulatory
Myl9	myosin, light chain 12B, regulatory
Mylc2b	myosin, light polypeptide kinase
Mylk	myosin IB
Myo1b	family with sequence similarity 129, member A
Nagk	N-acetylglucosamine kinase
Naglu	alpha-N-acetylglucosaminidase (Sanfilippo disease IIIB)
Nanos1	nanos homolog 1 (Drosophila)
Nanos2	nanos homolog 2 (Drosophila)
Nanos3	nanos homolog 3 (Drosophila)
Nckipsd	NCK interacting protein with SH3 domain
Ndufa10	NADH dehydrogenase (ubiquinone) 1 alpha subcomplex 10
Ndufa11	NADH dehydrogenase (ubiquinone) 1 alpha subcomplex 11
Ndufa12	NADH dehydrogenase (ubiquinone) 1 alpha subcomplex, 12
Ndufa13	NADH dehydrogenase (ubiquinone) 1 alpha subcomplex, 13
Ndufa2	NADH dehydrogenase (ubiquinone) 1 alpha subcomplex, 2
Ndufa5	NADH dehydrogenase (ubiquinone) 1 alpha subcomplex, 5
Ndufa6	NADH dehydrogenase (ubiquinone) 1 alpha subcomplex, 6 (B14)

Combined list of proteins	
**Protein Symbol**	**Protein Name**
Ndufa7	NADH dehydrogenase (ubiquinone) 1 alpha subcomplex, 7 (B14.5a)
Ndufa8	NADH dehydrogenase (ubiquinone) 1 alpha subcomplex, 8
Ndufa9	NADH dehydrogenase (ubiquinone) 1 alpha subcomplex, 9
Ndufab1	NADH dehydrogenase (ubiquinone) 1, alpha/beta subcomplex, 1
Ndufb10	NADH dehydrogenase (ubiquinone) 1 beta subcomplex, 10
Ndufb4	NADH dehydrogenase (ubiquinone) 1 beta subcomplex 4
Ndufb5	NADH dehydrogenase (ubiquinone) 1 beta subcomplex, 5
Ndufb7	NADH dehydrogenase (ubiquinone) 1 beta subcomplex, 7
Ndufc2	NADH dehydrogenase (ubiquinone) 1, subcomplex unknown, 2
Ndufs1	NADH dehydrogenase (ubiquinone) Fe-S protein 1
Ndufs2	NADH dehydrogenase (ubiquinone) Fe-S protein 2
Ndufs3	NADH dehydrogenase (ubiquinone) Fe-S protein 3
Ndufs4	NADH dehydrogenase (ubiquinone) Fe-S protein 4
Ndufs5	NADH dehydrogenase (ubiquinone) Fe-S protein 5
Ndufs6	NADH dehydrogenase (ubiquinone) Fe-S protein 6
Ndufs7	NADH dehydrogenase (ubiquinone) Fe-S protein 7
Ndufv1	NADH dehydrogenase (ubiquinone) flavoprotein 1
Ndufv2	NADH dehydrogenase (ubiquinone) flavoprotein 2
Neu1	neuraminidase 1
Neurl1a	neuralized homolog 1A (Drosophila)
Nfs1	nitrogen fixation gene 1 (S. cerevisiae)
Nhlrc1	NHL repeat containing 1
Niban	non-metastatic cells 2, protein (NM23B) expressed in
Nipbl	Nipped-B homolog (Drosophila)
Nipsnap3a	nipsnap homolog 3A (C. elegans)
Nkx3-1	NK-3 transcription factor, locus 1 (Drosophila)
Nme2	non-metastatic cells 2, protein (NM23B) expressed in
Nme5	non-metastatic cells 5, protein expressed in (nucleoside-diphosphate kinase)
Nme7	non-metastatic cells 7, protein expressed in (nucleoside-diphosphate kinase)
Nme8	NME/NM23 family member 8
Npepps	aminopeptidase puromycin sensitive
Nsf	N-ethylmaleimide sensitive fusion protein
Nsun4	NOL1/NOP2/Sun domain family, member 4
Nt5c1b	5'-nucleotidase, cytosolic IB

Combined list of proteins	
**Protein Symbol**	**Protein Name**
Nup153	nucleoporin 153
Nup210l	nucleoporin 210-like
Nup214	nucleoporin 214
Nup54	nucleoporin 54
Odf1	outer dense fiber of sperm tails 1
Odf2	outer dense fiber of sperm tails 2
Odf3	outer dense fiber of sperm tails 3
Odf4	outer dense fiber of sperm tails 4
Ogdh	oxoglutarate dehydrogenase (lipoamide)
Ogdhl	oxoglutarate dehydrogenase-like
Olfr504	olfactory receptor 504
Olfr905	olfactory receptor 905
Oplah	5-oxoprolinase (ATP-hydrolysing)
OTTMUSG00000005300	predicted gene 12070
Ovgp1	oviductal glycoprotein 1
Oxct2a	3-oxoacid CoA transferase 2A
Oxct2b	3-oxoacid CoA transferase 2B
P4hb	prolyl 4-hydroxylase, beta polypeptide
Pacrg	PARK2 co-regulated
Pafah1b1	platelet-activating factor acetylhydrolase, isoform 1b, subunit 1
Papolb	poly (A) polymerase beta (testis specific)
Parg	poly (ADP-ribose) glycohydrolase
Park2	Parkinson disease (autosomal recessive, juvenile) 2, parkin
Park7	Parkinson disease (autosomal recessive, early onset) 7
Parp1	poly (ADP-ribose) polymerase family, member 1
Parp2	poly (ADP-ribose) polymerase family, member 2
Pbp2	phosphatidylethanolamine binding protein 2
Pcdh7	protocadherin 7
Pclo	piccolo (presynaptic cytomatrix protein)
Pcmt1	protein-L-isoaspartate (D-aspartate) O-methyltransferase 1
Pcp4	Purkinje cell protein 4
Pcsk4	proprotein convertase subtilisin/kexin type 4
Pcsk6	proprotein convertase subtilisin/kexin type 6

Combined list of proteins	
**Protein Symbol**	**Protein Name**
Pcx	pyruvate carboxylase
Pcyox1	prenylcysteine oxidase 1
Pde11a	phosphodiesterase 11A
Pde1a	phosphodiesterase 1A, calmodulin-dependent
Pde1b	phosphodiesterase 1B, Ca2+-calmodulin dependent
Pde4a	phosphodiesterase 4A, cAMP specific
Pdha1	pyruvate dehydrogenase E1 alpha 1
Pdha2	pyruvate dehydrogenase E1 alpha 2
Pdhb	pyruvate dehydrogenase (lipoamide) beta
Pdhx	pyruvate dehydrogenase complex, component X
Pdia3	protein disulfide isomerase associated 3
Pdia4	protein disulfide isomerase associated 4
Pdia6	protein disulfide isomerase associated 6
Pdpk1	3-phosphoinositide dependent protein kinase-1
Pdzk1	PDZ domain containing 1
Pebp1	phosphatidylethanolamine binding protein 1
Pfkfb4	6-phosphofructo-2-kinase/fructose-2,6-biphosphatase 4
Pfkm	phosphofructokinase, muscle
Pfkp	phosphofructokinase, platelet
Pfn1	profilin 1
Pfn3	profilin 3
Pfn4	profilin family, member 4
Pgam1	phosphoglycerate mutase 1
Pgam2	phosphoglycerate mutase 2
Pgcp	plasma glutamate carboxypeptidase
Pgd	phosphogluconate dehydrogenase
Pgk1	phosphoglycerate kinase 1
Pgk2	phosphoglycerate kinase 2
Pgrmc1	progesterone receptor membrane component 1
Pgrmc2	progesterone receptor membrane component 2
Pgs1	phosphatidylglycerophosphate synthase 1
Phb	prohibitin
Phb2	prohibitin 2
Phf1	PHD finger protein 1

Combined list of proteins	
**Protein Symbol**	**Protein Name**
Phospho1	phosphatase, orphan 1
Pick1	protein interacting with C kinase 1
Pik3ca	phosphatidylinositol 3-kinase, catalytic, alpha polypeptide
Pik3cb	phosphatidylinositol 3-kinase, catalytic, beta polypeptide
Pik3cd	phosphatidylinositol 3-kinase catalytic delta polypeptide
Pik3cg	phosphoinositide-3-kinase, catalytic, gamma polypeptide
Pik3r1	phosphatidylinositol 3-kinase, regulatory subunit, polypeptide 1 (p85 alpha)
Pik3r3	phosphatidylinositol 3 kinase, regulatory subunit, polypeptide 3 (p55)
Pik3r4	phosphatidylinositol 3 kinase, regulatory subunit, polypeptide 4, p150
Pip	prolactin induced protein
Pitrm1	pitrilysin metallepetidase 1
Piwil1	piwi-like RNA-mediated gene silencing 1
Piwil2	piwi-like RNA-mediated gene silencing 2
Piwil4	piwi-like RNA-mediated gene silencing 4
Pkdrej	polycystic kidney disease (polycystin) and REJ (sperm receptor for egg jelly homolog, sea urchin)
Pkm2	pyruvate kinase, muscle
Pla2g1b	phospholipase A2, group IB, pancreas
Plag1	pleiomorphic adenoma gene 1
Plau	plasminogen activator, urokinase
Plb1	phospholipase B1
Plbd1	phospholipase B domain containing 1
Plcd4	phospholipase C, delta 4
Plekha5	pleckstrin homology domain containing, family A member 5
Plg	plasminogen
Pls3	plastin 3 (T-isoform)
Plscr1	phospholipid scramblase 1
Plscr4	phospholipid scramblase 4
Pmfbp1	polyamine modulated factor 1 binding protein 1
Pms2	postmeiotic segregation increased 2 (S. cerevisiae)
Pnpla7	patatin-like phospholipase domain containing 7
Ppap2a	phosphatidic acid phosphatase type 2A
Ppia	peptidylprolyl isomerase A
Ppm1b	protein phosphatase 1B, magnesium dependent, beta isoform
Ppp1cc	protein phosphatase 1, catalytic subunit, gamma isoform

Combined list of proteins	
**Protein Symbol**	**Protein Name**
Ppp1r11	protein phosphatase 1, regulatory (inhibitor) subunit 11
Ppp1r11	protein phosphatase 1, regulatory (inhibitor) subunit 11
Ppp1r7	protein phosphatase 1, regulatory (inhibitor) subunit 7
Ppp2r1a	protein phosphatase 2 (formerly 2A), regulatory subunit A (PR 65), alpha isoform
Ppp3cc	protein phosphatase 3, catalytic subunit, gamma isoform
Ppp3r2	protein phosphatase 3, regulatory subunit B, alpha isoform (calcineurin B, type II)
Ppp6c	protein phosphatase 6, catalytic subunit
Prcp	prolylcarboxypeptidase (angiotensinase C)
Prdm6	PR domain containing 6
Prdx1	peroxiredoxin 1
Prdx2	peroxiredoxin 2
Prdx5	peroxiredoxin 5
Prdx6	peroxiredoxin 6
Prdx6b	peroxiredoxin 6B
Prdx6-rs1	peroxiredoxin 6, related sequence 1
Prkaca	protein kinase, cAMP dependent, catalytic, alpha
Prkar1a	protein kinase, cAMP dependent regulatory, type I, alpha
Prkar2a	protein kinase, cAMP dependent regulatory, type II alpha
Prkcg	protein kinase C, gamma
Prkcsh	protein kinase C substrate 80K-H
Prkrir	protein-kinase, interferon-inducible double stranded RNA dependent inhibitor, repressor of (P58 repressor)
Prm1	protamine 1
Prm2	protamine 2
Prom1	prominin 1
Pros1	protein S (alpha)
Prpsap2	phosphoribosyl pyrophosphate synthetase-associated protein 2
Prrt2	proline-rich transmembrane protein 2
Prss21	protease, serine, 21
Prss32	protease, serine, 32
Psat1	phosphoserine aminotransferase 1
Psip1	PC4 and SFRS1 interacting protein 1
Psma1	proteasome (prosome, macropain) subunit, alpha type 1
Psma2	proteasome (prosome, macropain) subunit, alpha type 2
Psma3	proteasome (prosome, macropain) subunit, alpha type 3

Combined list of proteins	
**Protein Symbol**	**Protein Name**
Psma5	proteasome (prosome, macropain) subunit, alpha type 5
Psma6	proteasome (prosome, macropain) subunit, alpha type 6
Psma8	proteasome (prosome, macropain) subunit, alpha type, 8
Psmb1	proteasome (prosome, macropain) subunit, beta type 1
Psmb2	proteasome (prosome, macropain) subunit, beta type 2
Psmb3	proteasome (prosome, macropain) subunit, beta type 3
Psmb4	proteasome (prosome, macropain) subunit, beta type 4
Psmb5	proteasome (prosome, macropain) subunit, beta type 5
Psmb6	proteasome (prosome, macropain) subunit, beta type 6
Psmb7	proteasome (prosome, macropain) subunit, beta type 7
Psmc1	protease (prosome, macropain) 26S subunit, ATPase 1
Psmc2	proteasome (prosome, macropain) 26S subunit, ATPase 2
Psmc3	proteasome (prosome, macropain) 26S subunit, ATPase 3
Psmc3ip	proteasome (prosome, macropain) 26S subunit, ATPase 3, interacting protein
Psmc4	proteasome (prosome, macropain) 26S subunit, ATPase, 4
Psmc5	protease (prosome, macropain) 26S subunit, ATPase 5
Psmc6	proteasome (prosome, macropain) 26S subunit, ATPase, 6
Psmd1	proteasome (prosome, macropain) 26S subunit, non-ATPase, 1
Psmd11	proteasome (prosome, macropain) 26S subunit, non-ATPase, 11
Psmd13	proteasome (prosome, macropain) 26S subunit, non-ATPase, 13
Psmd14	proteasome (prosome, macropain) 26S subunit, non-ATPase, 14
Psmd2	proteasome (prosome, macropain) 26S subunit, non-ATPase, 2
Psmd3	proteasome (prosome, macropain) 26S subunit, non-ATPase, 3
Psmd6	proteasome (prosome, macropain) 26S subunit, non-ATPase, 6
Psmd7	proteasome (prosome, macropain) 26S subunit, non-ATPase, 7
Psmd8	proteasome (prosome, macropain) 26S subunit, non-ATPase, 8
Psme1	proteasome (prosome, macropain) 28 subunit, alpha
Ptchd3	patched domain containing 3
Ptgds	prostaglandin D2 synthase (brain)
ptgs1	prostaglandin-endoperoxide synthase 1
Ptgs2	prostaglandin-endoperoxide synthase 2
Ptk2b	PTK2 protein tyrosine kinase 2 beta
Ptpn1	protein tyrosine phosphatase, non-receptor type 1
Ptrh2	peptidyl-tRNA hydrolase 2

Combined list of proteins	
**Protein Symbol**	**Protein Name**
Pvrl2	poliovirus receptor-related 2
Pvrl3	poliovirus receptor-related 3
Pzp	pregnancy zone protein
Qsox1	quiescin Q6 sulfhydryl oxidase 1
Rab10	RAB10, member RAS oncogene family
Rab11b	RAB11B, member RAS oncogene family
Rab14	RAB14, member RAS oncogene family
Rab22a	RAB22A, member RAS oncogene family
Rab2a	RAB2A, member RAS oncogene family
Rab3a	RAB3A, member RAS oncogene family
Rab3gap2	RAB3 GTPase activating protein subunit 2
Rab5c	RAB5C, member RAS oncogene family
Rab8a	RAB8A, member RAS oncogene family
Rab8b	RAB8B, member RAS oncogene family
Rabggtb	RAB geranylgeranyl transferase, b subunit
Rad17	RAD17 homolog (S. pombe)
Rae1	RAE1 RNA export 1 homolog (S. pombe)
Rala	v-ral simian leukemia viral oncogene homolog A (ras related)
Rangrf	RAN guanine nucleotide release factor
Rapgef3	Rap guanine nucleotide exchange factor (GEF) 3
Rapgef4	Rap guanine nucleotide exchange factor (GEF) 4
Rbm12	RNA binding motif protein 12
Rbmxl2	RNA binding motif protein, X-linked-like 2
Rbmy	RNA binding motif protein, Y chromosome
Rbpj	recombination signal binding protein for immunoglobulin kappa J region
Rcn1	reticulocalbin 1
Rec8	REC8 homolog (yeast)
Reep6	receptor accessory protein 6
repro2	reproductive mutant 2, JAX Reproductive Mutagenesis Program
repro3	reproductive mutation 3, JAX Reproductive Mutagenesis Program
Rfx7	regulatory factor X, 7
Rgs22	regulator of G-protein signalling 22
Rhob	ras homolog gene family, member B
Rhox5	reproductive homeobox 5

Combined list of proteins	
**Protein Symbol**	**Protein Name**
Rhpn1	rhophilin, Rho GTPase binding protein 1
Rhpn2	rhophilin, Rho GTPase binding protein 2
Ribc2	RIB43A domain with coiled-coils 2
Rif1	Rap1 interacting factor 1 homolog (yeast)
Rnls	renalase, FAD-dependent amine oxidase
Ropn1	ropporin, rhophilin associated protein 1
Ropn1l	ropporin 1-like
Rpn1	ribophorin I
Rpn2	ribophorin II
Rpph1	ribonuclease P RNA component H1
Rprml	reprimo-like
Rps27a	ribosomal protein S27A
Rrbp1	ribosome binding protein 1
Rshl1	radial spokehead-like 1
Rshl2b	radial spokehead-like 2B
Rsph1	radial spoke head 1 homolog (Chlamydomonas)
Rsph9	radial spoke head 9 homolog (Chlamydomonas)
Rtdr1	rhabdoid tumor deletion region gene 1
Ryr3	ryanodine receptor 3
S100a6	S100 calcium binding protein A6 (calcyclin)
Sacm1l	SAC1 (suppressor of actin mutations 1, homolog)-like (S. cerevisiae)
Samm50	sorting and assembly machinery component 50 homolog (S. cerevisiae)
Sash3	SAM and SH3 domain containing 3
Satl1	spermidine/spermine N1-acetyl transferase-like 1
Scamp1	secretory carrier membrane protein 1
Scamp2	secretory carrier membrane protein 2
Sccpdh	saccharopine dehydrogenase (putative)
Scnn1b	sodium channel, nonvoltage-gated 1 beta
Sdccag8	serologically defined colon cancer antigen 8
Sdha	succinate dehydrogenase complex, subunit A, flavoprotein (Fp)
Sdhb	succinate dehydrogenase complex, subunit B, iron sulfur (Ip)
Sdhd	succinate dehydrogenase complex, subunit D, integral membrane protein
Sema6b	sema domain, transmembrane domain (TM), and cytoplasmic domain, (semaphorin) 6B
Senp6	SUMO/sentrin specific peptidase 6

Combined list of proteins	
**Protein Symbol**	**Protein Name**
Senp8	SUMO/sentrin specific peptidase 8
Sepp1	selenoprotein P, plasma, 1
Sept4	septin 4
Sept7	septin 7
Serpina1a	serine (or cysteine) peptidase inhibitor, clade A, member 1A
Serpina1b	serine (or cysteine) preptidase inhibitor, clade A, member 1B
Serpina1d	serine (or cysteine) peptidase inhibitor, clade A, member 1D
Serpina1e	serine (or cysteine) peptidase inhibitor, clade A, member 1E
Serpina1f	serine (or cysteine) peptidase inhibitor, clade A, member 1F
Serpina3k	serine (or cysteine) peptidase inhibitor, clade A, member 3K
Serpina5	serine (or cysteine) peptidase inhibitor, clade A, member 5
Serpinb6a	serine (or cysteine) peptidase inhibitor, clade B, member 6a
Serpine2	serine (or cysteine) peptidase inhibitor, clade E, member 2
Sgta	small glutamine-rich tetratricopeptide repeat (TPR)-containing, alpha
Sh3bgrl	SH3-binding domain glutamic acid-rich protein like
Shmt1	serine hydroxymethyltransferase 1 (soluble)
Siah1a	seven in absentia 1A
Skiv2l	superkiller viralicidic activity 2-like (S. cerevisiae)
Slc16a10	solute carrier family 16 (monocarboxylic acid transporters), member 10
Slc16a7	solute carrier family 16 (monocarboxylic acid transporters), member 7
Slc22a21	solute carrier family 22 (organic cation transporter), member 21
Slc22a5	solute carrier family 22 (organic cation transporter), member 5
Slc25a10	solute carrier family 25 (mitochondrial carrier, dicarboxylate transporter), member 10
Slc25a3	solute carrier family 25 (mitochondrial carrier, phosphate carrier), member 3
Slc25a31	solute carrier family 25 (mitochondrial carrier; adenine nucleotide translocator), member 31
Slc25a4	solute carrier family 25 (mitochondrial carrier, adenine nucleotide translocator), member 4
Slc26a8	solute carrier family 26, member 8
Slc26a8	solute carrier family 26, member 8
Slc2a3	solute carrier family 2 (facilitated glucose transporter), member 3
Slc2a3	solute carrier family 2 (facilitated glucose transporter), member 3
Slc2a5	solute carrier family 2 (facilitated glucose transporter), member 5
Slc9a1	solute carrier family 9 (sodium/hydrogen exchanger), member 1
Slc9a5	solute carrier family 9 (sodium/hydrogen exchanger), member 5
Slc9b2	solute carrier family 9, subfamily B (NHA2, cation proton antiporter 2), member 2

Combined list of proteins	
**Protein Symbol**	**Protein Name**
Slx	Sycp3 like X-linked
Slxl1	Slx-like 1
Sly	Sycp3 like Y-linked
Smc1b	structural maintenance of chromosomes 1B
Smcp	sperm mitochondria-associated cysteine-rich protein
Smok1	sperm motility kinase 1
Smok2a	sperm motility kinase 2A
Smok2b	sperm motility kinase 2B
Smok4a	sperm motility kinase 4A
Snap25	synaptosomal-associated protein 25
Sod1	superoxide dismutase 1, soluble
Sod2	superoxide dismutase 2, mitochondrial
Sord	sorbitol dehydrogenase
Sort1	sortilin 1
Spa17	sperm autoantigenic protein 17
Spa17	sperm autoantigenic protein 17
Spaca1	sperm acrosome associated 1
Spaca3	sperm acrosome associated 3
Spaca3	sperm acrosome associated 3
Spaca4	sperm acrosome associated 4
Spaca5	sperm acrosome associated 5
Spag1	sperm associated antigen 1
Spag11a	sperm associated antigen 11A
Spag16	sperm associated antigen 16
Spag17	sperm associated antigen 17
Spag4	sperm associated antigen 4
Spag5	sperm associated antigen 5
Spag6	sperm associated antigen 6
Spag7	sperm associated antigen 7
Spag8	sperm associated antigen 8
Spag9	sperm associated antigen 9
Spam1	sperm adhesion molecule 1
Spam1	sperm adhesion molecule 1
Spata16	spermatogenesis associated 16

Combined list of proteins	
**Protein Symbol**	**Protein Name**
Spata17	spermatogenesis associated 17
Spata18	spermatogenesis associated 18
Spata19	spermatogenesis associated 19
Spata20	spermatogenesis associated 20
Spata21	spermatogenesis associated 21
Spata24	spermatogenesis associated 24
Spata3	spermatogenesis associated 3
Spata5	spermatogenesis associated 5
Spata6	spermatogenesis associated 6
Spata9	spermatogenesis associated 9
Spatc1	spermatogenesis and centriole associated 1
Spcs2	signal peptidase complex subunit 2 homolog (S. cerevisiae)
Spem1	sperm maturation 1
Spesp1	sperm equatorial segment protein 1
Spg20	spastic paraplegia 20, spartin (Troyer syndrome) homolog (human)
Sphk1	sphingosine kinase 1
Spink5	serine peptidase inhibitor, Kazal type 5
Spinkl	serine protease inhibitor, Kazal type-like
Spnb2	spectrin beta 2
Spo11	SPO11 meiotic protein covalently bound to DSB homolog (S. cerevisiae)
Sqrdl	sulfide quinone reductase-like (yeast)
Srgn	serglycin
Srpk2	serine/arginine-rich protein specific kinase 2
Srpk3	serine/arginine-rich protein specific kinase 3
Srsf2	serine/arginine-rich splicing factor 2
Ssna1	Sjogren's syndrome nuclear autoantigen 1
St13	suppression of tumorigenicity 13
Stard6	StAR-related lipid transfer (START) domain containing 6
Stub1	STIP1 homology and U-Box containing protein 1
Stx1b	syntaxin 1B
Stx2	syntaxin 2
Stxbp3a	syntaxin binding protein 3A
Sucla2	succinate-Coenzyme A ligase, ADP-forming, beta subunit
Suclg1	succinate-CoA ligase, GDP-forming, alpha subunit

Combined list of proteins	
**Protein Symbol**	**Protein Name**
Suco	SUN domain containing ossification factor
Suox	sulfite oxidase
swm2	sperm without mobility 2
Syce1	synaptonemal complex central element protein 1
Syce2	synaptonemal complex central element protein 2
Sycp2	synaptonemal complex protein 2
Sycp3	synaptonemal complex protein 3
Syt1	synaptotagmin I
Syt6	synaptotagmin VI
Syt8	synaptotagmin VIII
Sytl1	synaptotagmin-like 1
Sytl2	synaptotagmin-like 2
Taf7l	TAF7-like RNA polymerase II, TATA box binding protein (TBP)-associated factor
Tagln	transgelin
Tagln2	transgelin 2
Tbc1d17	TBC1 domain family, member 17
Tbc1d20	TBC1 domain family, member 20
Tbl1x	transducin (beta)-like 1 X-linked
Tbl2	transducin (beta)-like 2
Tcp1	t-complex protein 1
Tcp11	t-complex protein 11
Tcte1	t-complex-associated testis expressed 1
Tcte3	t-complex-associated testis expressed 3
Tctex1d1	Tctex1 domain containing 1
Tctex1d2	Tctex1 domain containing 2
Tctex1d4	Tctex1 domain containing 4
Tekt1	tektin 1
Tekt2	tektin 2
Tekt3	tektin 3
Tekt4	tektin 4
Tekt5	tektin 5
Tepp	testis, prostate and placenta expressed
Tesk1	testis specific protein kinase 1
Tex101	testis expressed gene 101

Combined list of proteins	
**Protein Symbol**	**Protein Name**
Tex11	testis expressed gene 11
Tex14	testis expressed gene 14
Tg(PRG1)18Wlad	transgene insertion 18, Wyeth Lederle/Anne Deatly
Tgm4	transglutaminase 4 (prostate)
Thap4	THAP domain containing 4
Theg	testicular haploid expressed gene
Them4	thioesterase superfamily member 4
Thnsl1	threonine synthase-like 1 (bacterial)
Tia1	cytotoxic granule-associated RNA binding protein 1
Tial1	Tia1 cytotoxic granule-associated RNA binding protein-like 1
Timm44	translocase of inner mitochondrial membrane 44
Timm50	translocase of inner mitochondrial membrane 50 homolog (yeast)
Timp1	tissue inhibitor of metalloproteinase 1
Tkt	transketolase
Tmed10	transmembrane emp24-like trafficking protein 10 (yeast)
Tmem146	transmembrane protein 146
Tmem147	transmembrane protein 147
Tmem177	transmembrane protein 177
Tmem190	transmembrane protein 190
Tmem30a	transmembrane protein 30A
Tmprss2	transmembrane protease, serine 2
Tnp1	transition protein 1
Tnp2	transition protein 2
Tnr	tenascin R
Tomm22	translocase of outer mitochondrial membrane 22 homolog (yeast)
Tomm40	translocase of outer mitochondrial membrane 40 homolog (yeast)
Tpi1	triosephosphate isomerase 1
Tpm1	tropomyosin 1, alpha
Tpm3	tropomyosin 3, gamma
Tpp2	tripeptidyl peptidase II
Tra2b	transformer 2 beta homolog (Drosophila)
Trappc6b	trafficking protein particle complex 6B
Trappc8	trafficking protein particle complex 8
Trf	transferrin

Combined list of proteins	
**Protein Symbol**	**Protein Name**
Trim36	tripartite motif-containing 36
Trip13	thyroid hormone receptor interactor 13
Trpc1	transient receptor potential cation channel, subfamily C, member 1
Trpc2	transient receptor potential cation channel, subfamily C, member 2
Trpc3	transient receptor potential cation channel, subfamily C, member 3
Trpc4	transient receptor potential cation channel, subfamily C, member 4
Trpc6	transient receptor potential cation channel, subfamily C, member 6
Trrap	transformation/transcription domain-associated protein
try10	trypsin 10
Tsga10	testis specific 10
Tsks	testis-specific serine kinase substrate
Tsp50	testes-specific protease 50
Tspo	translocator protein
Tssk1	testis-specific serine kinase 1
Tssk2	testis-specific serine kinase 2
Tssk3	testis-specific serine kinase 3
Tssk4	testis-specific serine kinase 4
Tssk6	testis-specific serine kinase 6
Tssk6	testis-specific serine kinase 6
Ttc25	tetratricopeptide repeat domain 25
Ttc39d	tetratricopeptide repeat domain 39D
Ttc8	tetratricopeptide repeat domain 8
Ttn	titin
Ttr	transthyretin
Tuba1a	tubulin, alpha 1A
Tuba1a	tubulin, alpha 1A
Tuba1b	tubulin, alpha 1B
Tuba1c	tubulin, alpha 1C
Tuba3a	tubulin, alpha 3A
Tuba3a	tubulin, alpha 3A
Tuba3b	tubulin, alpha 3B
Tubb2b	tubulin, beta 2B
Tubb2c	tubulin, beta 2C
Tubb3	tubulin, beta 3

Combined list of proteins	
**Protein Symbol**	**Protein Name**
Tubb4	tubulin, beta 4
Tubd1	tubulin, delta 1
Txndc2	thioredoxin domain containing 2 (spermatozoa)
Txndc3	thioredoxin domain containing 3 (spermatozoa)
Tyk2	tyrosine kinase 2
Uba52	ubiquitin A-52 residue ribosomal protein fusion product 1
Uba6	ubiquitin-like modifier activating enzyme 6
Ubb	ubiquitin B
Ube1x	ubiquitin-like modifier activating enzyme 1
Ube2b	ubiquitin-conjugating enzyme E2B
Ube2v2	ubiquitin-conjugating enzyme E2 variant 2
Ubqln1	ubiquilin 1
Ubtd2	ubiquitin domain containing 2
Ubxn11	UBX domain protein 11
Ubxn8	UBX domain protein 8
Uchl1	ubiquitin carboxy-terminal hydrolase L1
Uchl3	ubiquitin carboxyl-terminal esterase L3 (ubiquitin thiolesterase)
Uchl4	ubiquitin carboxyl-terminal esterase L4
Uchl5	ubiquitin carboxyl-terminal esterase L5
Ugcgl1	UDP-glucose glycoprotein glucosyltransferase 1
Ugp2	UDP-glucose pyrophosphorylase 2
Uqcr	ubiquinol-cytochrome c reductase (6.4kD) subunit
Uqcrb	ubiquinol-cytochrome c reductase binding protein
Uqcrc1	ubiquinol-cytochrome c reductase core protein 1
Uqcrc2	ubiquinol cytochrome c reductase core protein 2
Uqcrfs1	ubiquinol-cytochrome c reductase, Rieske iron-sulfur polypeptide 1
Uqcrq	ubiquinol-cytochrome c reductase, complex III subunit VII
Usp26	ubiquitin specific peptidase 26
Usp4	ubiquitin specific peptidase 4 (proto-oncogene)
Usp7	ubiquitin specific peptidase 7
Vamp3	vesicle-associated membrane protein 3
Vamp5	vesicle-associated membrane protein 5
Vapa	vesicle-associated membrane protein, associated protein A
Vcl	vinculin

Combined list of proteins	
**Protein Symbol**	**Protein Name**
Vcp	valosin containing protein
Vdac1	voltage-dependent anion channel 1
Vdac2	voltage-dependent anion channel 2
Vdac3	voltage-dependent anion channel 3
Vil2	ezrin
Vim	vimentin
Vps13a	vacuolar protein sorting 13A (yeast)
Wars	tryptophanyl-tRNA synthetase
Was	Wiskott-Aldrich syndrome homolog (human)
Wbp7	WW domain binding protein 7
Wdr16	WD repeat domain 16
Wdr49	WD repeat domain 49
Wdr52	WD repeat domain 52
Wdr64	WD repeat domain 64
Wnk2	WNK lysine deficient protein kinase 2
Xpnpep1	X-prolyl aminopeptidase (aminopeptidase P) 1, soluble
Xpo7	exportin 7
Ybx2	Y box protein 2
Yes1	Yamaguchi sarcoma viral (v-yes) oncogene homolog 1
Ywhab	tyrosine 3-monooxygenase/tryptophan 5-monooxygenase activation protein, beta polypeptide
Ywhae	tyrosine 3-monooxygenase/tryptophan 5-monooxygenase activation protein, epsilon polypeptide
Ywhaz	tyrosine 3-monooxygenase/tryptophan 5-monooxygenase activation protein, zeta polypeptide
Zan	zonadhesin
Zcchc8	zinc finger, CCHC domain containing 8
Zfa-ps	zinc finger protein, autosomal, pseudogene
Zfp112	zinc finger protein 112
Zfp395	zinc finger protein 395
Zfp407	zinc finger protein 407
Zfp474	zinc finger protein 474
Zfp828	zinc finger protein 828
Zp3r	zona pellucida 3 receptor
Zpbp	zona pellucida binding protein
Zpbp2	zona pellucida binding protein 2
