# Supplementary material for: Proteins Involved in Motility and Sperm-Egg Interaction Evolve More Rapidly in Mouse Spermatozoa
Source: PLoS One. 2014 Mar 7;9(3):e91302. doi: 10.1371/journal.pone.0091302 (PMC3948348; doi:10.1371/journal.pone.0091302)
Supplement: Table S2 — List of proteins used in the study. (PDF) [file pone.0091302.s002.pdf]

Table S2. List of proteins used in the study.

Protein	Protein name	Reproductive process	Subcelullar location	Molecular function
Acrbp	Acrosin binding protein	Acrosome reaction	Head	Binding
Afaf	Acrosome formation- associated factor	Acrosome reaction	Head	Binding
Akt1	RAC-alpha serine/threonine-protein kinase	Acrosome reaction	Head	Catalytic
Cacna1h	Voltage-dependent T-type calcium channel subunit alpha-1H	Acrosome reaction	Head	Transport
Cd46	CD46 antigen, complement regulatory protein	Acrosome reaction	Head	Binding
Enkur	Enkurin	Acrosome reaction	Head and flagellum	Binding
Glra1	Glycine receptor subunit alpha 1	Acrosome reaction	Head	Transport
Itpr1	Inositol 1,4,5-trisphosphate receptor type 1	Acrosome reaction	Head.	Transport
Pdpk1	3-phosphoinositide dependent protein kinase 1	Acrosome reaction	Head and flagellum	Catalytic
Pik3c3	Phosphoinositide-3-kinase, class 3	Acrosome reaction	Head	Catalytic
Pik3r3	Phosphatidil inositol 3 kinasa regulatory subunit 3	Acrosome reaction	Head	Catalytic
Pla2g1b	Phospholipase A2	Acrosome reaction	Head	Catalytic
Plcd4	Phospholipase C delta 4	Acrosome reaction	Head	Catalytic
Prkcz	Protein kinase C zeta type	Acrosome reaction	Head	Catalytic
Ptpn1	Tyrosine-protein phosphatase non-receptor type 1	Acrosome reaction	Head	Catalytic
Syt6	Synaptotagmin-6	Acrosome reaction	Head	Binding
Syt8	Synaptotagmin-8	Acrosome reaction	Head	Binding
Trim36	E3 ubiquitin-protein ligase Trim36	Acrosome reaction	Head	Catalytic
Trpc2	Short transient receptor potential channel 2	Acrosome reaction	Head	Transport
Vcp	Transitional endoplasmic reticulum ATPase	Acrosome reaction	Head	Binding, transport
Adcy2	Adenylate cyclase type 2	Capacitation	Head	Catalytic
Adcy8	Adenylate cyclase type 8	Capacitation	Head and flagellum	Catalytic
Cabyr	Calcium-binding tyrosine phosphorilation-regulated protein	Capacitation	Flagellum	Binding
Cacna1c	Voltage-dependent L-type calcium channel subunit alpha-1C	Capacitation	Head and flagellum	Transport
Calca	Calcitonin-related polypeptide, alpha	Capacitation	Head and flagellum	Binding
Calm1	Calmodulin	Capacitation	Head and flagellum	Binding
Dld	Dihydrolipoamide dehydrogenase	Capacitation	Head and flagellum	Catalytic
Fsip2	Fibrous sheath-interacting protein 2	Capacitation	Flagellum	Binding
Pcsk4	Proprotein convertase subtilisin/kexin type 4	Capacitation	Head	Catalytic
Pde4d	Phospodiesterase 4d	Capacitation	Head and flagellum	Catalytic
Plscr2	Phospholipid scramblase 2	Capacitation	Head	Transport
Ppp3r2	Calcineurin subunit B type 2	Capacitation	Head	Catalytic, binding
Prkaca	Sperm -specific protein kinase A catalytic subunit	Capacitation	Head	Binding
Pyk2	Protein-tyrosine kinase 2-beta	Capacitation	Head and flagellum	Catalytic
Rab14	Ras-related protein Rab-14	Capacitation	Head	Transport

Protein	Protein name	Reproductive process	Subcelullar location	Molecular function
Sacy	Soluble adenylate cyclase	Capacitation	Head and flagellum	Catalytic
Slo3	Potassium channel subfamily U member 1	Capacitation	Flagellum	Transport
Tex101	Testis-expressed protein 101	Capacitation	Head	Catalytic
Trpc3	Short transient receptor potential channel 3	Capacitation	Flagellum	Transport
Pebp1	Phosphatidylethanolamine-binding protein 1	Capacitation	Head and flagellum	Binding
Svs2	Semenogelin-1	Capacitation	Head	Structural
Aldoa1	Fructosa bisphosphate aldolase	Sperm metabolism	Flagellum	Catalytic
Atp5b	ATP synthetase beta subunit, mitochondrial	Sperm metabolism	Head and flagellum	Catalytic
Cox6b2	Cytochrome c oxidase subunit 6B2	Sperm metabolism	Flagellum	Catalytic
Csl	Cytrate synthase-like gene	Sperm metabolism	Flagellum	Catalytic
Cyct	Cytochrome c, testis-specific	Sperm metabolism	Flagellum	Binding, transport
Dbil5	Diazepam-binding inhibitor-like 5	Sperm metabolism	Flagellum	Binding, transport
Dlat	Dihydrolipoyllysine-residue acetyltransferase	Sperm metabolism	Flagellum	Catalytic
Eno1	Enolase 1	Sperm metabolism	Flagellum	Catalytic, binding
G6pd2	Glucose-6-phosphate 1-dehydrogenase 2	Sperm metabolism	Flagellum	Catalytic
Gapdhs	Glyceraldehyde-3-phosphate dehydrogenase, spermatogenic	Sperm metabolism	Flagellum	Catalytic
Glut3	Solute carrier family 2, facilitated glucose transporter member 3	Sperm metabolism	Head	Transport
Gpd2	Glycerol phospate deshydrogenase 2	Sperm metabolism	Flagellum	Binding
Gpi1	Glucose phosphate isomerase	Sperm metabolism	Flagellum	Catalytic
Hk1s	Hexokinase-1, spermatogenic cell-specific	Sperm metabolism	Head and flagellum	Catalytic, binding
Idh1	Isocitrate dehydrogenase [NADP] cytoplasmic	Sperm metabolism	Head	Catalytic, binding
Ldhc	L-lactate dehydrogenase C chain	Sperm metabolism	Flagellum	Catalytic
Mpi	Mannose-6-phosphate isomerase	Sperm metabolism	Flagellum	Catalytic, binding
Oxct2a	Succinyl-CoA:3-ketoacid-coenzyme A transferase 2A, mitochondrial	Sperm metabolism	Flagellum	Unknown
Pdha2	Piruvate dehydrogenase A2	Sperm metabolism	Flagellum	Catalytic
Pfkfb4	6-phosphofructo-2-kinase/fructose-2,6-biphosphatase 4	Sperm metabolism	Flagellum	Catalytic, binding
Pgam2	Phosphoglycerate mutase 2	Sperm metabolism	Flagellum	catalytic
Pgk2	Phosphoglycerate kinase 2	Sperm metabolism	Flagellum	Catalytic
Pkm2	Pyruvate kinase isozymes M1/M2	Sperm metabolism	Flagellum	Catalytic, binding
Ak1	Adenylate kinase 1	Sperm motility	Flagellum	Catalytic
Akap110	A- kinase anchor protein 3	Sperm motility	Flagellum	Binding
Akap82	A- kinase anchor protein 82	Sperm motility	Head and flagellum	Binding
Atp1a4	Sodium/potassium-transporting ATPase subunit alpha-4	Sperm motility	Flagellum	Transport
Car4	Carbonic anhydrase-4	Sperm motility	Head	Catalytic
Catsper1	Cation channel sperm associated protein 1	Sperm motility	Flagellum	Transport
CatSper2	Cation channel sperm associated protein 2	Sperm motility	Flagellum	Transport
Catsper3	Cation channel sperm associated protein 3	Sperm motility	Flagellum	Binding

Protein	Protein name	Reproductive process	Subcelullar location	Molecular function
Catsper4	Cation channel sperm associated protein 4	Sperm motility	Flagellum	Transport
CatsperG	Cation channel sperm associated protein G	Sperm motility	Flagellum	Transport
Chdh	Choline dehydrogenase	Sperm motility	Flagellum	Catalytic
Chrna7	Neuronal acetylcholine receptor subunit alpha-7	Sperm motility	Flagellum	Transport
Dnah12	Dynein heavy chain 12	Sperm motility	Flagellum	Catalytic, binding
Gas8	Growth arrest-specific protein 8	Sperm motility	Flagellum	Structural
Ift88	Intraflagellar transport protein 88 homolog	Sperm motility	Flagellum	Binding, transport
Neurl1a	Neuralized-like protein 1A	Sperm motility	Flagellum	Binding
Nhe1	Sodium/hydrogen exchanger 1	Sperm motility	Flagellum	Transport
Nhe10	Sodium/hydrogen exchanger 10	Sperm motility	Flagellum	Transport
Nhe5	Sodium/hydrogen exchanger 5	Sperm motility	Flagellum	Transport
Odf4	Outer dense fiber protein-4	Sperm motility	Flagellum	Structural
Pmca4	Plasma membrane calcium/calmodulin-dependent calcium ATPase, isoform 4	Sperm motility	Flagellum	Transport
Rhpn1	Rhophilin-1	Sperm motility	Flagellum	Binding
Ropn1	Ropporin-1	Sperm motility	Flagellum	Binding
Slc25a1	Solute carrier family 25 member 1	Sperm motility	Flagellum	Binding
Smcp	Sperm mitochondria-associated cysteine-rich protein.	Sperm motility	Flagellum	Structural
Smky	Sperm motility Kinase Y	Sperm motility	Flagellum	Catalytic
Smok2a	Sperm motility kinase 2A	Sperm motility	Flagellum	Catalytic
Smok2b	Sperm motility kinase 2B	Sperm motility	Flagellum	Catalytic
Smok4a	Sperm motility kinase 4A	Sperm motility	Flagellum	Catalytic
Spag6	Sperm associated antigen 6	Sperm motility	Flagellum	Binding
Tctex1	Dynein light chain Tctex-type 1	Sperm motility	Flagellum	Transport
Tctex5	Dynein light chain Tctex-type 5	Sperm motility	Head and flagellum	Catalytic
Tekt3	Tektin-3	Sperm motility	Flagellum	Binding
Tekt4	Tektin-4	Sperm motility	Flagellum	Binding
Boll	Boule-like protein	Spermatogenesis	Head	Binding
Ccna1	Cyclin A1	Spermatogenesis	Head	Binding
Celf3	CUGBP Elav-like family member 3	Spermatogenesis	Head	Binding
Cttn	Cotractine	Spermatogenesis	Head	Structural
Dazla	Deleted in azoospermia-like	Spermatogenesis	Head	Binding
Ddx4	Probable ATP-dependent RNA helicase	Spermatogenesis	Head	Binding
Dnmt3l	DNA (cytosine-5)-methyltransferase 3-like	Spermatogenesis	Head	Catalytic
Etv5	ETS translocation variant 5	Spermatogenesis	Head	Binding
Fkbp6	Peptidyl-prolyl cis-trans isomerase FKBP6	Spermatogenesis	Head	Catalytic
Fscb	Fibrous sheat CABYR-binding protein	Spermatogenesis	Flagellum	Binding
H1fnt	Testis-specific H1 histone	Spermatogenesis	Head	Binding

Protein	Protein name	Reproductive process	Subcelullar location	Molecular function
Hils1	Histone H1-like protein in spermatids 1	Spermatogenesis	Head	Binding
Krt9	Keratin-9	Spermatogenesis	Flagellum	Structural
Prm1	Protamine 1	Spermatogenesis	Head	Binding
Prm2	Protamine 2	Spermatogenesis	Head	Binding
Pvrl2	Nectin 2	Spermatogenesis	Flagellum	Binding
Rsph1	Radial spoke head 1 homolog	Spermatogenesis	Head and flagellum	Binding
Sept4	Septin-4	Spermatogenesis	Flagellum	Structural
Spata16	Spermatogenesis-associated protein 16	Spermatogenesis	Head	Binding
Spata24	Spermatogenesis-associated protein 24	Spermatogenesis	Head	Binding
Spatc1	Speriolin	Spermatogenesis	Flagellum	Binding
Sptrx1	Thioredoxin domain-containing protein 2	Spermatogenesis	Flagellum	Catalytic
Stx2	Sintaxin-2	Spermatogenesis	Head	Binding, transport
Taf7l	Transcription initiation factor TFIID subunit 7-like	Spermatogenesis	Flagellum	Binding
Tekt2	Tektin-2	Spermatogenesis	Flagellum	Structural
Tnp1	Transition protein-1	Spermatogenesis	Head	Binding
Tnp2	Transition protein-2	Spermatogenesis	Head	Binding
Tssk1	Testis-specific serine/threonine-protein kinase-1	Spermatogenesis	Head and flagellum	Catalytic
Tssk2	Testis-specific serine/threonine-protein kinase-2	Spermatogenesis	Flagellum	Catalytic
Tssk6	Testis-specific serine/threonine-protein kinase-6	Spermatogenesis	Head	Catalytic
Vdac3	Voltage-dependent-anion channel mitochondrial	Spermatogenesis	Flagellum	Transport
Dpep3	Dipeptidase 3	Spermatogenesis	Head	Catalytic
Pdia3	Disulfide isomerase A3	Spermatogenesis	Head and flagellum	Catalytic
Ace	Angiotensin-converting enzyme	Sperm-egg interaction	Head	Catalytic
Acrv1	Acrosomal protein SP-10	Sperm-egg interaction	Head	Catalytic
Adam18	A disintegrin and metallopeptidase domain 18	Sperm-egg interaction	Head	Binding
Adam1a	A disintegrin and metallopeptidase domain 1a	Sperm-egg interaction	Head	Binding
Adam1b	A disintegrin and metallopeptidase domain 1b	Sperm-egg interaction	Head	Binding
Adam2	A disintegrin and metallopeptidase domain 2	Sperm-egg interaction	Head	Binding
Adam24	A disintegrin and metallopeptidase domain 24	Sperm-egg interaction	Head	Catalytic
Adam3	A disintegrin and metallopeptidase domain 3	Sperm-egg interaction	Head	Binding
Adam32	A disintegrin and metallopeptidase domain 32	Sperm-egg interaction	Head	Catalytic
Arsa	Arysulfatase A	Sperm-egg interaction	Head	Catalytic
Atp8b3	ATPase class I type 8B member 3	Sperm-egg interaction	Head	Catalytic, transport
B4galt1	Beta-1,4 galactosyltransferase 1	Sperm-egg interaction	Head	Catalytic
Bsg	Basigin	Sperm-egg interaction	Head and flagellum	Binding
Clgn	Calmegin	Sperm-egg interaction	Head	Binding

Protein	Protein name	Reproductive process	Subcelullar location	Molecular function
Crisp1	Cysteine-rich secretory protein 1	Sperm-egg interaction	Head and flagellum	Unknown
Crisp2	Cysteine-rich secretory protein 2	Sperm-egg interaction	Head and flagellum	Binding
Crisp4	Cysteine-rich secretory protein 4	Sperm-egg interaction	Head and flagellum	
Ctsl1	Cysteine-specific cathepsin	Sperm-egg interaction	Head	Catalytic
Dkkl1	Dickkofp-like 1	Sperm-egg interaction	Head	
Hyal5	Hyaluronidase-5	Sperm-egg interaction	Head	Catalytic
Izumo1	Izumo sperm-egg fusion protein 1	Sperm-egg interaction	Head	Binding
Mgea5	Beta-N-acetylhexosaminidase	Sperm-egg interaction	Head	Catalytic
Ph20	Hyaluronidase PH-20	Sperm-egg interaction	Head and flagellum	Catalytic
Pkdrej	Polycystic kidney disease and receptor for egg jelly-related protein	Sperm-egg interaction	Head	Transport
Sed1	Bimotif EGF Repeat and Discoidin-Domain Protein-1	Sperm-egg interaction	Head	Binding
Slxl1	Putative novel protein similar to Xlr-related, meiosis regulated Xmr	Sperm-egg interaction	Head	Transport
Sp56	Zona pellucida sperm-binding protein 3 receptor	Sperm-egg interaction	Head	Binding
Spaca3	Sperm acrosome membrane-associated protein 3	Sperm-egg interaction	Head	Catalytic
Spesp1	Sperm equatorial segment protein 1	Sperm-egg interaction	Head	
Tcte1	T-complex-associated testis-expressed protein 1	Sperm-egg interaction	Head	
Tmem190	Transmembrane protein 190	Sperm-egg interaction	Head	Unknown
Zan	Zonadhesin	Sperm-egg interaction	Head	Binding
Zpbp1	Zona pellucida binding protein-1	Sperm-egg interaction	Head	Binding
Zpbp2	Zona pellucida binding protein-2	Sperm-egg interaction	Head	Binding

* Subcellular location was inferred based on proteomic studies, Gene Ontology annotation (cellular component) databases and a review of literature.
† Molecular function was determinated using Gene Ontology annotation (molecular function) databases.
