# Supplementary material for: Proteins Involved in Motility and Sperm-Egg Interaction Evolve More Rapidly in Mouse Spermatozoa
Source: PLoS One. 2014 Mar 7;9(3):e91302. doi: 10.1371/journal.pone.0091302 (PMC3948348; doi:10.1371/journal.pone.0091302)
Supplement: Table S3 — Values of non-synonymous (dN), synonymous (dS) and dN/dS (ω) ratios estimated for sperm genes. Sperm genes with a dN/dS ratio >0.5 are shown in bold. (PDF) [file pone.0091302.s003.pdf]

Table S3. Values of non-synonymous (dN), synonymous (dS) and dN/dS (ω) ratios estimated for sperm genes. Sperm genes with a dN/dS ratio > 0.5 are shown in bold.

Gene	Gene name	Reproductive process	dN	dS	dN/dS
Crisp1	**Cysteine-rich secretory protein 1**	**Sperm-egg interaction**	**0.553**	**0.4769**	**1.16493**
Fscb	**Fibrous sheat CABYR-binding protein**	**Spermatogenesis**	**0.0321**	**0.0279**	**1.1505**
Slxl1	**Putative novel protein similar to Xlr-related, meiosis regulated Xmr**	**Sperm-egg interaction**	**0.0649**	**0.0573**	**1.1326**
Smcp	**Sperm mitochondria-associated cysteine-rich protein.**	**Sperm motility**	**0.4891**	**0.4326**	**1.13**
Hils1	**Histone H1-like protein in spermatids 1**	**Spermatogenesis**	**0.2182**	**0.1978**	**1.1031**
Tmem190	**Transmembrane protein 190**	**Sperm-egg interaction**	**0.0866**	**0.0806**	**1.0744**
Izumo1	**Izumo sperm-egg fusion protein 1**	**Sperm-egg interaction**	**0.2261**	**0.2983**	**0.75819**
Sp56	**Zona pellucida sperm-binding protein 3 receptor**	**Sperm-egg interaction**	**0.1452**	**0.1931**	**0.75216**
Cd46	**CD46 antigen, complement regulatory protein**	**Acrosome reaction**	**0.1301**	**0.1809**	**0.7193**
Adam24	**A disintegrin and metallopeptidase domain 24**	**Sperm-egg interaction**	**0.15**	**0.2106**	**0.7123**
CatSper2	**Cation channel sperm associated protein 2**	**Sperm motility**	**0.1489**	**0.2158**	**0.69023**
Spesp1	**Sperm equatorial segment protein 1**	**Sperm-egg interaction**	**0.1408**	**0.2062**	**0.6828**
Krt9	**Keratin-9**	**Spermatogenesis**	**0.131**	**0.1971**	**0.6646**
Sptrx1	**Thioredoxin domain-containing protein 2**	**Spermatogenesis**	**0.2032**	**0.3226**	**0.6299**
Ph20	**Hyaluronidase PH-20**	**Sperm-egg interaction**	**0.1374**	**0.2206**	**0.62279**
Acrv1	**Acrosomal protein SP-10**	**Sperm-egg interaction**	**0.0939**	**0.151**	**0.6219**
Svs2	**Semenogelin-1**	**Capacitation**	**0.1858**	**0.3076**	**0.60407**
Car4	**Carbonic anhydrase-4**	**Sperm motility**	**0.176**	**0.3023**	**0.5822**
H1fnt	**Testis-specific H1 histone**	**Spermatogenesis**	**0.1358**	**0.2374**	**0.5720**
G6pd2	**Glucose-6-phosphate 1-dehydrogenase 2**	**Sperm metabolism**	**0.0744**	**0.1386**	**0.53679**
Afaf	**Acrosome formation- associated factor**	**Acrosome reaction**	**0.1321**	**0.2504**	**0.5276**
Taf7l	**Transcription initiation factor TFIID subunit 7-like**	**Spermatogenesis**	**0.1449**	**0.2767**	**0.5237**
Smky	**Sperm motility Kinase Y**	**Sperm motility**	**0.1456**	**0.2823**	**0.5158**
Smok2b	**Sperm motility kinase 2B**	**Sperm motility**	**0.4949**	**0.9823**	**0.50384**
Mpi	**Mannose-6-phosphate isomerase**	**Sperm metabolism**	**0.0667**	**0.1334**	**0.5001**
Smok4a	Sperm motility kinase 4A	Sperm motility	0.4125	0.828	0.49819
Smok2a	Sperm motility kinase 2A	Sperm motility	0.4706	0.9454	0.49772
CatsperG	Cation channel sperm associated protein G	Sperm motility	0.1402	0.2942	0.4764
Hyal5	Hyaluronidase-5	Sperm-egg interaction	0.103	0.2178	0.47264
Adam32	A disintegrin and metallopeptidase domain 32	Sperm-egg interaction	0.1205	0.2585	0.4662
Catsper4	Cation channel sperm associated protein 4	Sperm motility	0.095	0.461	0.461
Fsip2	Fibrous sheath-interacting protein 2	Capacitation	0.0102	0.0223	0.4574
Tex101	Testis-expressed protein 101	Capacitation	0.1123	0.2511	0.4472

Gene	Gene name	Reproductive process	dN	dS	dN/dS
Cabyr	Calcium-binding tyrosine phosphorilation-regulated protein	Capacitation	0.064	0.147	0.4354
Tctex5	Dynein light chain Tctex-type 5	Sperm motility	0.0553	0.1281	0.4317
Prm1	Protamine 1	Spermatogenesis	0.0551	0.1277	0.4315
Odf4	Outer dense fiber protein-4	Sperm motility	0.1147	0.2841	0.4037
Catsper1	Cation channel sperm associated protein 1	Sperm motility	0.1751	0.4338	0.4037
Adam3	A disintegrin and metallopeptidase domain 3	Sperm-egg interaction	0.1184	0.2965	0.39911
Zan	Zonadhesin	Sperm-egg interaction	0.0179	0.0457	0.39209
Adam18	A disintegrin and metallopeptidase domain 18	Sperm-egg interaction	0.1068	0.2747	0.3888
Spatc1	Speriolin	Spermatogenesis	0.012	0.0316	0.3797
Cox6b2	Cytochrome c oxidase subunit 6B2	Sperm metabolism	0.0404	0.1081	0.3740
Crisp2	Cysteine-rich secretory protein 2	Sperm-egg interaction	0.0886	0.2463	0.35949
Adam2	A disintegrin and metallopeptidase domain 2	Sperm-egg interaction	0.089	0.2491	0.35708
Pkdrej	Polycystic kidney disease and receptor for egg jelly-related protein	Sperm-egg interaction	0.1015	0.2861	0.3548
Adam1a	A disintegrin and metallopeptidase domain 1a	Sperm-egg interaction	0.1041	0.3036	0.34298
Slo3	Potassium channel subfamily U member 1	Capacitation	0.0061	0.0181	0.33557
Pik3c3	Phosphoinositide-3-kinase, class 3	Acrosome reaction	0.0117	0.0039	0.3335
Tctex1	Dynein light chain Tctex-type 1	Sperm motility	0.0557	0.1678	0.3319
Spaca3	Sperm acrosome membrane-associated protein 3	Sperm-egg interaction	0.0574	0.1762	0.32554
Prm2	Protamine 2	Spermatogenesis	0.0445	0.1369	0.3251
Syt8	Synaptotagmin-8	Acrosome reaction	0.0559	0.1894	0.29489
Pgk2	Phosphoglycerate kinase 2	Sperm metabolism	0.0582	0.1985	0.2932
Plcd4	Phospholipase C delta 4	Acrosome reaction	0.0622	0.2145	0.28989
Pla2g1b	Phospholipase A2	Acrosome reaction	0.0686	0.2527	0.27136
Aldoa1	Fructosa bisphosphate aldolase	Sperm metabolism	0.0067	0.0254	0.2638
Ldhc	L-lactate dehydrogenase C chain	Sperm metabolism	0.0559	0.2126	0.2629
Adam1b	A disintegrin and metallopeptidase domain 1b	Sperm-egg interaction	0.0113	0.0434	0.26053
Dpep3	Dipeptidase 3	Spermatogenesis	0.0536	0.2185	0.2453
Zpbp1	Zona pellucida binding protein-1	Sperm-egg interaction	0.0302	0.1231	0.24512
Zpbp2	Zona pellucida binding protein-2	Sperm-egg interaction	0.079	0.323	0.24457
Rsph1	Radial spoke head 1 homolog	Spermatogenesis	0.0659	0.2728	0.2416
Ppp3r2	Calcineurin subunit B type 2	Capacitation	0.0481	0.2015	0.2387
Tnp2	Transition protein-2	Spermatogenesis	0.0524	0.2198	0.2384
Dnmt3l	DNA (cytosine-5)-methyltransferase 3-like	Spermatogenesis	0.0687	0.2911	0.2360
Itpr1	Inositol 1,4,5-trisphosphate receptor type 1	Acrosome reaction	0.1323	0.5815	0.2275
Tcte1	T-complex-associated testis-expressed protein 1	Sperm-egg interaction	0.0577	0.2554	0.2259

Gene	Gene name	Reproductive process	dN	dS	dN/dS
Tekt3	Tektin-3	Sperm motility	0.0547	0.2527	0.2165
Pmca4	Plasma membrane calcium/calmodulin-dependent calcium ATPase, isoform 4	Sperm motility	0.0503	0.2333	0.21565
Pde4d	Phospodiesterase 4d	Capacitation	0.0184	0.0037	0.2024
Bsg	Basigin	Sperm-egg interaction	0.0605	0.3	0.2016
Tnp1	Transition protein-1	Spermatogenesis	0.0185	0.0977	0.1894
Atp8b3	ATPase class I type 8B member 3	Sperm-egg interaction	0.0643	0.3411	0.1886
Enkur	Enkurin	Acrosome reaction	0.061	0.3328	0.18345
Plscr2	Phospholipid scramblase 2	Capacitation	0.0558	0.3071	0.18178
B4galt1	Beta-1,4 galactosyltransferase 1	Sperm-egg interaction	0.0344	0.1898	0.18125
Oxct2a	Succinyl-CoA:3-ketoacid-coenzyme A transferase 2A, mitochondrial	Sperm metabolism	0.07	0.387	0.1809
Vdac3	Voltage-dependent-anion channel mitochondrial	Spermatogenesis	0.0221	0.1225	0.1804
Catsper3	Cation channel sperm associated protein 3	Sperm motility	0.0779	0.4351	0.17901
Dkkl1	Dickkofp-like 1	Sperm-egg interaction	0.0593	0.3393	0.1747
Acrbp	Acrosin binding protein	Acrosome reaction	0.0415	0.2418	0.1717
Akap110	A- kinase anchor protein 3	Sperm motility	0.0402	0.2369	0.1697
Spata16	Spermatogenesis-associated protein 16	Spermatogenesis	0.0413	0.2568	0.1608
Clgn	Calmegin	Sperm-egg interaction	0.0464	0.2896	0.16029
Sed1	Bimotif EGF Repeat and Discoidin-Domain Protein-1	Sperm-egg interaction	0.0352	0.2218	0.1587
Trpc2	Short transient receptor potential channel 2	Acrosome reaction	0.0393	0.2595	0.15123
Tssk1	Testis-specific serine/threonine-protein kinase-1	Spermatogenesis	0.017	0.1126	0.1510
Crisp4	Cysteine-rich secretory protein 4	Sperm-egg interaction	0.0593	0.3992	0.1485
Tekt4	Tektin-4	Sperm motility	0.049	0.337	0.1454
Dlat	Dihydrolipoyllysine-residue acetyltransferase	Sperm metabolism	0.0307	0.2145	0.1430
Chdh	Choline dehydrogenase	Sperm motility	0.018	0.1272	0.1414
Gapdhs	Glyceraldehyde-3-phosphate dehydrogenase, spermatogenic	Sperm metabolism	0.0464	0.3317	0.13997
Calca	Calcitonin-related polypeptide, alpha	Capacitation	0.0195	0.1042	0.13906
Dnah12	Dynein heavy chain 12	Sperm motility	0.0329	0.2419	0.1360
Akap82	A- kinase anchor protein 82	Sperm motility	0.0296	0.2227	0.13267
Tekt2	Tektin-2	Spermatogenesis	0.0325	0.2485	0.13083
Ctsl1	Cysteine-specific cathepsin	Sperm-egg interaction	0.0329	0.2529	0.1301
Atp1a4	Sodium/potassium-transporting ATPase subunit alpha-4	Sperm motility	0.0349	0.2692	0.1298
Rhpn1	Rhophilin-1	Sperm motility	0.0395	0.3124	0.1264
Ropn1	Ropporin-1	Sperm motility	0.0233	0.186	0.1253
Arsa	Arysulfatase A	Sperm-egg interaction	0.0233	0.1903	0.1224
Ptpn1	Tyrosine-protein phosphatase non-receptor type 1	Acrosome reaction	0.036	0.2964	0.1215

Gene	Gene name	Reproductive process	dN	dS	dN/dS
Dbil5	Diazepam-binding inhibitor-like 5	Sperm metabolism	0.0286	0.2386	0.1199
Nhe10	Sodium/hydrogen exchanger 10	Sperm motility	0.0538	0.4505	0.11934
Pdha2	Piruvate dehydrogenase A2	Sperm metabolism	0.0363	0.3062	0.11866
Ace	Angiotensin-converting enzyme	Sperm-egg interaction	0.0332	0.2992	0.1110
Ccna1	Cyclin A1	Spermatogenesis	0.022	0.1989	0.1106
Pdpk1	3-phosphoinositide dependent protein kinase 1	Acrosome reaction	0.0109	0.1025	0.10628
Glut3	Solute carrier family 2, facilitated glucose transporter member 3	Sperm metabolism	0.0292	0.2869	0.1018
Gpi1	Glucose phosphate isomerase	Sperm metabolism	0.0306	0.3058	0.1001
Pdia3	Disulfide isomerase A3	Spermatogenesis	0.0157	0.1598	0.0982
Ak1	Adenylate kinase 1	Sperm motility	0.0304	0.3164	0.0961
Sacy	Soluble adenylate cyclase	Capacitation	0.0275	0.2882	0.0954
Cttn	Cotractine	Spermatogenesis	0.0149	0.1567	0.0951
Pvrl2	Nectin 2	Spermatogenesis	0.0207	0.231	0.0896
Gpd2	Glycerol phospate deshydrogenase 2	Sperm metabolism	0.0243	0.2767	0.0878
Boll	Boule-like protein	Spermatogenesis	0.0131	0.1523	0.0860
Pcsk4	Proprotein convertase subtilisin/kexin type 4	Capacitation	0.0195	0.2289	0.0853
Stx2	Sintaxin-2	Spermatogenesis	0.0049	0.0576	0.0850
Pik3r3	Phosphatidil inositol 3 kinasa regulatory subunit 3	Acrosome reaction	0.0197	0.2356	0.0837
Dld	Dihydrolipoamide dehydrogenase	Capacitation	0.018	0.227	0.07944
Etv5	ETS translocation variant 5	Spermatogenesis	0.0155	0.2015	0.0769
Slc25a1	Solute carrier family 25 member 1	Sperm motility	0.0162	0.2145	0.0755
Idh1	Isocitrate dehydrogenase [NADP] cytoplasmic	Sperm metabolism	0.0153	0.2124	0.0720
Pebp1	Phosphatidylethanolamine-binding protein 1	Capacitation	0.0362	0.5161	0.07005
Trpc3	Short transient receptor potential channel 3	Capacitation	0.0237	0.3435	0.0690
Ddx4	Probable ATP-dependent RNA helicase	Spermatogenesis	0.02	0.2932	0.0682
Pkm2	Pyruvate kinase isozymes M1/M2	Sperm metabolism	0.0329	0.4922	0.0669
Tssk2	Testis-specific serine/threonine-protein kinase-2	Spermatogenesis	0.0084	0.1341	0.0626
Cacna1h	Voltage-dependent T-type calcium channel subunit alpha-1H	Acrosome reaction	0.0174	0.2789	0.06226
Fkbp6	Peptidyl-prolyl cis-trans isomerase FKBP6	Spermatogenesis	0.0132	0.2126	0.0621
Pgam2	Phosphoglycerate mutase 2	Sperm metabolism	0.0157	0.2592	0.0605
Cacna1c	Voltage-dependent L-type calcium channel subunit alpha-1C	Capacitation	0.0133	0.2256	0.05903
Csl	Cytrate synthase-like gene	Sperm metabolism	0.0043	0.0732	0.0584
Mgea5	Beta-N-acetylhexosaminidase	Sperm-egg interaction	0.0129	0.2292	0.0565
Hk1s	Hexokinase-1, spermatogenic cell-specific	Sperm metabolism	0.0161	0.2939	0.0549
Eno1	Enolase 1	Sperm metabolism	0.0155	0.2849	0.0544

Gene	Gene name	Reproductive process	dN	dS	dN/dS
Spata24	Spermatogenesis-associated protein 24	Spermatogenesis	0.0143	0.2714	0.0527
Gas8	Growth arrest-specific protein 8	Sperm motility	0.0246	0.4897	0.05023
Cyct	Cytochrome c, testis-specific	Sperm metabolism	0.017	0.3388	0.0502
Sept4	Septin-4	Spermatogenesis	0.0104	0.2083	0.0499
Nhe5	Sodium/hydrogen exchanger 5	Sperm motility	0.0096	0.2054	0.04665
Atp5b	ATP synthetase beta subunit, mitochondrial	Sperm metabolism	0.0078	0.1681	0.0464
Pyk2	Protein-tyrosine kinase 2-beta	Capacitation	0.002	0.0462	0.0433
Syt6	Synaptotagmin-6	Acrosome reaction	0.0096	0.2588	0.03718
Celf3	CUGBP Elav-like family member 3	Spermatogenesis	0.004	0.1103	0.0363
Prkcz	Protein kinase C zeta type	Acrosome reaction	0	0.0058	0.03498
Trim36	E3 ubiquitin-protein ligase Trim36	Acrosome reaction	0.0071	0.2019	0.03495
Adcy8	Adenylate cyclase type 8	Capacitation	0.0051	0.1929	0.02669
Chrna7	Neuronal acetylcholine receptor subunit alpha-7	Sperm motility	0.0039	0.153	0.02526
Pfkfb4	6-phosphofructo-2-kinase/fructose-2,6-biphosphatase 4	Sperm metabolism	0.0048	0.1891	0.0251
Ift88	Intraflagellar transport protein 88 homolog	Sperm motility	0.0048	0.1891	0.0251
Neurl1a	Neuralized-like protein 1A	Sperm motility	0.0023	0.0941	0.0247
Adcy2	Adenylate cyclase type 2	Capacitation	0.005	0.2241	0.02223
Rab14	Ras-related protein Rab-14	Capacitation	0.0021	0.0976	0.0211
Spag6	Sperm associated antigen 6	Sperm motility	0.0049	0.2339	0.02084
Prkaca	Sperm -specific protein kinase A catalytic subunit	Capacitation	0.0024	0.1476	0.0166
Dazla	Deleted in azoospermia-like	Spermatogenesis	0.0018	0.1476	0.0122
Tssk6	Testis-specific serine/threonine-protein kinase-6	Spermatogenesis	0.0016	0.149	0.0107
Nhe1	Sodium/hydrogen exchanger 1	Sperm motility	0.0251	3.3664	0.00745
Glra1	Glycine receptor subunit alpha 1	Acrosome reaction	0.0019	0.3554	0.0053
Vcp	Transitional endoplasmic reticulum ATPase	Acrosome reaction	0.0006	0.1645	0.0035
Akt1	RAC-alpha serine/threonine-protein kinase	Acrosome reaction	0.0317	1.1551	0.00312
Calm1	Calmodulin	Capacitation	0	0.0058	0.0000
