# Supplementary material for: Proteins Involved in Motility and Sperm-Egg Interaction Evolve More Rapidly in Mouse Spermatozoa
Source: PLoS One. 2014 Mar 7;9(3):e91302. doi: 10.1371/journal.pone.0091302 (PMC3948348; doi:10.1371/journal.pone.0091302)
Supplement: Table S4 — Analysis of positive selection in sperm genes. Results of likelihood ratio test (LRT) comparing the likelihood values obtained in models M8 and M8a are presented. Evidence of positive selection is detected as the comparison of the LRT with the 50∶50 mixture of point mass 0 and χ2 is greater than the critical value 5.41 at 1% of significance. Infered positively selected sites with BEB posterior probabilities >0.95 (*) and >0.99 (**) are shown. (PDF) [file pone.0091302.s004.pdf]

Table S4. Analysis of positive selection in sperm genes. Results of likelihood ratio test (LRT) comparing the likelihood values obtained in models M8 and M8a are presented. Evidence of positive selection is detected as the comparison of the LRT with the 50:50 mixture of point mass 0 and χ_2 is greater than the critical value 5.41 at 1% of significance. Infered positively selected sites with BEB posterior probabilities > 0.95 (*) and > 0.99 (**) are shown.

**Protein**	**Protein name**	**Reproductive process**	**LRT M8vsM8a**	**Positive selection M8vsM8a (df = 2)**
G6pd2	Glucose-6-phosphate 1-dehydrogenase 2	Sperm metabolism	104.84	Yes
Fscb	Fibrous sheat CABYR-binding protein	Spermatogenesis	85.52	Yes
Smky	Sperm motility Kinase Y	Sperm motility	85.18	Yes
Chdh	Choline dehydrogenase	Sperm motility	48.80	Yes
Adam24	A disintegrin and metallopeptidase domain 24	Sperm-egg interaction	44.03	Yes
Smok2a	Sperm motility kinase 2A	Sperm motility	40.73	Yes
Idh1	Isocitrate dehydrogenase [NADP] cytoplasmic	Sperm metabolism	38.24	Yes
Ropn1	Ropporin-1	Sperm motility	34.38	Yes
Smok2b	Sperm motility kinase 2B	Sperm motility	33.33	Yes
Gapdhs	Glyceraldehyde-3-phosphate dehydrogenase, spermatogenic	Sperm metabolism	31.18	Yes
Pgk2	Phosphoglycerate kinase 2	Sperm metabolism	27.31	Yes
Krt9	Keratin-9	Spermatogenesis	26.95	Yes
Fsip2	Fibrous sheath-interacting protein 2	Capacitation	24.44	Yes
Crisp1	Cysteine-rich secretory protein 1	Sperm-egg interaction	24.08	Yes
Atp1a4	Sodium/potassium-transporting ATPase subunit alpha-4	Sperm motility	24.07	Yes
Afaf	Acrosome formation- associated factor	Acrosome reaction	22.45	Yes
Adam18	A disintegrin and metallopeptidase domain 18	Sperm-egg interaction	21.93	Yes
Sp56	Zona pellucida sperm-binding protein 3 receptor	Sperm-egg interaction	21.48	Yes
Prm1	Protamine 1	Spermatogenesis	21.40	Yes
Adam32	A disintegrin and metallopeptidase domain 32	Sperm-egg interaction	21.01	Yes
Car4	Carbonic anhydrase-4	Sperm motility	19.31	Yes
Nhe5	Sodium/hydrogen exchanger 5	Sperm motility	19.26	Yes
Tekt4	Tektin-4	Sperm motility	18.75	Yes
Pdia3	Disulfide isomerase A3	Spermatogenesis	18.72	Yes
Smcp	Sperm mitochondria-associated cysteine-rich protein.	Sperm motility	15.36	Yes
CatsperG	Cation channel sperm associated protein G	Sperm motility	13.77	Yes
Plscr2	Phospholipid scramblase 2	Capacitation	13.69	Yes
Tctex5	Dynein light chain Tctex-type 5	Sperm motility	13.26	Yes
Slxl1	Putative novel protein similar to Xlr-related, meiosis regulated Xmr	Sperm-egg interaction	12.56	Yes
Pkm2	Pyruvate kinase isozymes M1/M2	Sperm metabolism	12.38	Yes
Catsper3	Cation channel sperm associated protein 3	Sperm motility	12.31	Yes

Protein	Protein name	Reproductive process	LRT M8vsM8a	Positive selection M8vsM8a (df = 2)
Tnp2	Transition protein-2	Spermatogenesis	11.98	Yes
Zan	Zonadhesin	Sperm-egg interaction	11.97	Yes
Celf3	CUGBP Elav-like family member 3	Spermatogenesis	11.91	Yes
Ph20	Hyaluronidase PH-20	Sperm-egg interaction	11.65	Yes
Tmem190	Transmembrane protein 190	Sperm-egg interaction	10.44	Yes
CatSper2	Cation channel sperm associated protein 2	Sperm motility	10.15	Yes
Spatc1	Speriolin	Spermatogenesis	9.76	Yes
Rsph1	Radial spoke head 1 homolog	Spermatogenesis	9.59	Yes
Sptrx1	Thioredoxin domain-containing protein 2	Spermatogenesis	9.39	Yes
Dlat	Dihydrolipoyllysine-residue acetyltransferase	Sperm metabolism	8.82	Yes
Acrv1	Acrosomal protein SP-10	Sperm-egg interaction	8.45	Yes
Clgn	Calmegin	Sperm-egg interaction	8.29	Yes
Cacna1c	Voltage-dependent L-type calcium channel subunit alpha-1C	Capacitation	7.23	Yes
Spata16	Spermatogenesis-associated protein 16	Spermatogenesis	6.76	Yes
Zpbp2	Zona pellucida binding protein-2	Sperm-egg interaction	5.94	Yes
Mgea5	Beta-N-acetylhexosaminidase	Sperm-egg interaction	5.78	Yes
Trpc3	Short transient receptor potential channel 3	Capacitation	5.47	Yes
Oxct2a	Succinyl-CoA:3-ketoacid-coenzyme A transferase 2A, mitochondrial	Sperm metabolism	5.12	No
Zpbp1	Zona pellucida binding protein-1	Sperm-egg interaction	5.04	No
Cabyr	Calcium-binding tyrosine phosphorilation-regulated protein	Capacitation	5.04	No
Prm2	Protamine 2	Spermatogenesis	4.45	No
Hyal5	Hyaluronidase-5	Sperm-egg interaction	4.30	No
Gpd2	Glycerol phospate deshydrogenase 2	Sperm metabolism	4.30	No
Tnp1	Transition protein-1	Spermatogenesis	3.55	No
Taf7l	Transcription initiation factor TFIID subunit 7-like	Spermatogenesis	3.39	No
Plcd4	Phospholipase C delta 4	Acrosome reaction	3.39	No
Mpi	Mannose-6-phosphate isomerase	Sperm metabolism	3.34	No
Stx2	Sintaxin-2	Spermatogenesis	3.28	No
Pde4d	Phospodiesterase 4d	Capacitation	3.25	No
Pla2g1b	Phospholipase A2	Acrosome reaction	2.93	No
Ak1	Adenylate kinase 1	Sperm motility	2.64	No
Eno1	Enolase 1	Sperm metabolism	2.35	No
Rhpn1	Rhophilin-1	Sperm motility	2.13	No

Protein	Protein name	Reproductive process	LRT M8vsM8a	Positive selection M8vsM8a (df = 2)
Ppp3r2	Calcineurin subunit B type 2	Capacitation	2.02	No
Adam1b	A disintegrin and metallopeptidase domain 1b	Sperm-egg interaction	1.93	No
Atp8b3	ATPase class I type 8B member 3	Sperm-egg interaction	1.80	No
Chrna7	Neuronal acetylcholine receptor subunit alpha-7	Sperm motility	1.77	No
Boll	Boule-like protein	Spermatogenesis	1.74	No
Cacna1h	Voltage-dependent T-type calcium channel subunit alpha-1H	Acrosome reaction	1.70	No
Tssk6	Testis-specific serine/threonine-protein kinase-6	Spermatogenesis	1.65	No
Bsg	Basigin	Sperm-egg interaction	1.36	No
Sed1	Bimotif EGF Repeat and Discoidin-Domain Protein-1	Sperm-egg interaction	1.02	No
Pvrl2	Nectin 2	Spermatogenesis	0.98	No
Hils1	Histone H1-like protein in spermatids 1	Spermatogenesis	0.90	No
Tekt3	Tektin-3	Sperm motility	0.89	No
Ldhc	L-lactate dehydrogenase C chain	Sperm metabolism	0.80	No
Ptpn1	Tyrosine-protein phosphatase non-receptor type 1	Acrosome reaction	0.73	No
Dbil5	Diazepam-binding inhibitor-like 5	Sperm metabolism	0.65	No
Cttn	Cotractine	Spermatogenesis	0.58	No
Dpep3	Dipeptidase 3	Spermatogenesis	0.56	No
Adcy2	Adenylate cyclase type 2	Capacitation	0.34	No
Dnmt3l	DNA (cytosine-5)-methyltransferase 3-like	Spermatogenesis	0.32	No
Acrbp	Acrosin binding protein	Acrosome reaction	0.30	No
Tctex1	Dynein light chain Tctex-type 1	Sperm motility	0.20	No
Tcte1	T-complex-associated testis-expressed protein 1	Sperm-egg interaction	0.20	No
Sept4	Septin-4	Spermatogenesis	0.18	No
Pebp1	Phosphatidylethanolamine-binding protein 1	Capacitation	0.15	No
Gpi1	Glucose phosphate isomerase	Sperm metabolism	0.07	No
Pgam2	Phosphoglycerate mutase 2	Sperm metabolism	0.07	No
Slc25a1	Solute carrier family 25 member 1	Sperm motility	0.07	No
Ctsl1	Cysteine-specific cathepsin	Sperm-egg interaction	0.04	No
Aldoa1	Fructosa bisphosphate aldolase	Sperm metabolism	0.01	No
Prkaca	Sperm -specific protein kinase A catalytic subunit	Capacitation	0.01	No
Akt1	RAC-alpha serine/threonine-protein kinase	Acrosome reaction	0.00	No
Tssk1	Testis-specific serine/threonine-protein kinase-1	Spermatogenesis	0.00	No
Pcsk4	Proprotein convertase subtilisin/kexin type 4	Capacitation	0.00	No
B4galt1	Beta-1,4 galactosyltransferase 1	Sperm-egg interaction	0.00	No
Hk1s	Hexokinase-1, spermatogenic cell-specific	Sperm metabolism	0.00	No

Protein	Protein name	Reproductive process	LRT M8vsM8a	Positive selection M8vsM8a (df = 2)
Spesp1	Sperm equatorial segment protein 1	Sperm-egg interaction	0.00	No
Calm1	Calmodulin	Capacitation	0.00	No
Vdac3	Voltage-dependent-anion channel mitochondrial	Spermatogenesis	0.00	No
Vcp	Transitional endoplasmic reticulum ATPase	Acrosome reaction	0.00	No
Prkcz	Protein kinase C zeta type	Acrosome reaction	0.00	No
Arsa	Arysulfatase A	Sperm-egg interaction	0.00	No
Calca	Calcitonin-related polypeptide, alpha	Capacitation	0.00	No
Spaca3	Sperm acrosome membrane-associated protein 3	Sperm-egg interaction	0.00	No
Enkur	Enkurin	Acrosome reaction	0.00	No
Spata24	Spermatogenesis-associated protein 24	Spermatogenesis	0.00	No
Pik3c3	Phosphoinositide-3-kinase, class 3	Acrosome reaction	0.00	No
Cox6b2	Cytochrome c oxidase subunit 6B2	Sperm metabolism	0.00	No
Crisp2	Cysteine-rich secretory protein 2	Sperm-egg interaction	0.00	No
Itpr1	Inositol 1,4,5-trisphosphate receptor type 1	Acrosome reaction	0.00	No
Dkkl1	Dickkofp-like 1	Sperm-egg interaction	0.00	No
Ccna1	Cyclin A1	Spermatogenesis	0.00	No
Pdpk1	3-phosphoinositide dependent protein kinase 1	Acrosome reaction	0.00	No
Atp5b	ATP synthetase beta subunit, mitochondrial	Sperm metabolism	0.00	No
Pfkfb4	6-phosphofructo-2-kinase/fructose-2,6-biphosphatase 4	Sperm metabolism	0.00	No
Ift88	Intraflagellar transport protein 88 homolog	Sperm motility	0.00	No
Rab14	Ras-related protein Rab-14	Capacitation	0.00	No
Dazla	Deleted in azoospermia-like	Spermatogenesis	0.00	No
Glra1	Glycine receptor subunit alpha 1	Acrosome reaction	0.00	No
Adcy8	Adenylate cyclase type 8	Capacitation	0.00	No
Cyct	Cytochrome c, testis-specific	Sperm metabolism	0.00	No
Tex101	Testis-expressed protein 101	Capacitation	0.00	No
Fkbp6	Peptidyl-prolyl cis-trans isomerase FKBP6	Spermatogenesis	0.00	No
Csl	Cytrate synthase-like gene	Sperm metabolism	0.00	No
Ace	Angiotensin-converting enzyme	Sperm-egg interaction	0.00	No
Syt6	Synaptotagmin-6	Acrosome reaction	0.00	No
Pik3r3	Phosphatidil inositol 3 kinasa regulatory subunit 3	Acrosome reaction	0.00	No
Tssk2	Testis-specific serine/threonine-protein kinase-2	Spermatogenesis	0.00	No
Akap110	A- kinase anchor protein 3	Sperm motility	0.00	No
Glut3	Solute carrier family 2, facilitated glucose transporter member 3	Sperm metabolism	-0.01	No
Etv5	ETS translocation variant 5	Spermatogenesis	-0.01	No

Protein	Protein name	Reproductive process	LRT M8vsM8a	Positive selection M8vsM8a (df = 2)
Akap82	A- kinase anchor protein 82	Sperm motility	-0.01	No
Sacy	Soluble adenylate cyclase	Capacitation	-0.03	No
Gas8	Growth arrest-specific protein 8	Sperm motility	-0.05	No
Pyk2	Protein-tyrosine kinase 2-beta	Capacitation	-0.06	No
Dnah12	Dynein heavy chain 12	Sperm motility	-0.10	No
Ddx4	Probable ATP-dependent RNA helicase	Spermatogenesis	-0.87	No
Trim36	E3 ubiquitin-protein ligase Trim36	Acrosome reaction	-1.41	No
Odf4	Outer dense fiber protein-4	Sperm motility	-1.49	No
Izumo1	Izumo sperm-egg fusion protein 1	Sperm-egg interaction	-2.06	No
Crisp4	Cysteine-rich secretory protein 4	Sperm-egg interaction	-2.99	No
Neurl1a	Neuralized-like protein 1A	Sperm motility	-3.15	No
Adam3	A disintegrin and metallopeptidase domain 3	Sperm-egg interaction	-3.80	No
Syt8	Synaptotagmin-8	Acrosome reaction	-4.08	No
Dld	Dihydrolipoamide dehydrogenase	Capacitation	-6.55	No
Adam2	A disintegrin and metallopeptidase domain 2	Sperm-egg interaction	-8.19	No
H1fnt	Testis-specific H1 histone	Spermatogenesis	-9.84	No
Pkdrej	Polycystic kidney disease and receptor for egg jelly-related protein	Sperm-egg interaction	-11.25	No
Tekt2	Tektin-2	Spermatogenesis	-15.23	No
Pmca4	Plasma membrane calcium/calmodulin-dependent calcium ATPase, isoform 4	Sperm motility	-22.61	No
Trpc2	Short transient receptor potential channel 2	Acrosome reaction	-23.05	No
Svs2	Semenogelin-1	Capacitation	-24.43	No
Catsper4	Cation channel sperm associated protein 4	Sperm motility	-27.34	No
Catsper1	Cation channel sperm associated protein 1	Sperm motility	-27.89	No
Slo3	Potassium channel subfamily U member 1	Capacitation	-28.12	No
Spag6	Sperm associated antigen 6	Sperm motility	-34.96	No
Adam1a	A disintegrin and metallopeptidase domain 1a	Sperm-egg interaction	-40.16	No
Smok4a	Sperm motility kinase 4A	Sperm motility	-45.89	No
Cd46	CD46 antigen, complement regulatory protein	Acrosome reaction	-56.32	No
Pdha2	Piruvate dehydrogenase A2	Sperm metabolism	-65.57	No
Nhe1	Sodium/hydrogen exchanger 1	Sperm motility	-179.12	No
Nhe10	Sodium/hydrogen exchanger 10	Sperm motility	-190.45	No

Protein	Positively selected sites
G6pd2	231A**, 294R**
Fscb	105I*, 459S**, 551P**, 572F**, 857G**, 878M*, 978S**
Smky	29K*, 34L**, 46K**, 60L**, 65C**, 69A**, 75I**, 91F**, 115S*, 140G*, 141L**, 156D*, 179H*, 186G**, 199T**, 222I**, 243N**, 246K*, 273R*, 276C**, 280T**, 287V**, 298A*, 330E**, 359E**, 372S*, 381P*, 390V**, 402S**, 407L**, 409E**, 410P**, 415P**, 461R**, 494Q**
Chdh	4V**, 12W**, 24Q**
Adam24	453H**, 620D**, 677N*, 720E**
Smok2a	7Q*, 36S*, 38K**, 98R*, 109A**, 174G*, 181T*, 224T*, 240M*
Idh1	229K*
Ropn1	149N*
Smok2b	89R*, 101A*, 173G*, 180T*, 204V**, 374S*
Gapdhs	
Pgk2	151Q**, 176T**
Krt9	154A*, 168P*, 499H*, 532H*, 548G*, 572H*, 586G*, 587S*, 589G*, 591Y*, 592G*, 594G*, 595S*
Fsip2	1247R*, 2310T*, 2627N*, 2903P*, 3311R*, 3547I*, 5639S*, 5755T*, 5844R*, 5903E*, 6823Y*
Crisp1	112K**, 125V*, 201K*, 208S*, 212E*
Atp1a4	
Afaf	50A*, 123T**
Adam18	35H*, 145N*, 159T*, 327R*
Sp56	7I*, 8E**, 228L**, 519A**
Prm1	46I**
Adam32	154I*, 342S*, 422R*, 427A*, 456E*
Car4	97V**
Nhe5	
Tekt4	4T*, 145T*, 210S**, 262Q**
Pdia3	20R*
Smcp	51P*, 69P*, 74Q*, 109T**, 130N*
CatsperG	323H*
Plscr2	177K (**)
Tctex5	23T*, 79S**
Slxl1	34I**, 76E**
Pkm2	385L*, 388I**, 393T*
Catsper3	

Protein	Positively selected sites
Tnp2	
Zan	104Q**, 692A**, 703Y*, 779Y*, 868T**, 878L**, 956V*, 959V**, 997S**, 1032E*, 1042V*, 1645V**, 1767M**, 2714A**, 2765D**, 2854T*, 2889M**, 3109T**, 3203*, 3214**, 3587T**, 3671P**, 3690S**, 3720D**, 3761V**, 3853K**, 3897H**, 3898N*, 4032G**, 4132T**, 4300G**, 4307S**, 4358T*, 4363N*, 4490E**, 4533D*, 4674I**, 5216A*
Celf3	
Ph20	8H*, 97L*, 123R*
Tmem190	117H*, 143V*, 144L*
CatSper2	49A*, 490I**
Spatc1	
Rsph1	189T*, 238P*, 260L*
Sptrx1	266S*, 272Q*, 323E*, 387G*
Dlat	172D**
Acrv1	
Clgn	2R**, 291K*
Cacna1c	
Spata16	
Zpbp2	
Mgea5	
Trpc3	
Oxct2a	
Zpbp1	
Cabyr	
Prm2	
Hyal5	
Gpd2	
Tnp1	
Taf7l	
Plcd4	
Mpi	
Stx2	
Pde4d	
Pla2g1b	
Ak1	
Eno1	
Rhpn1	

Protein	Positively selected sites
Ppp3r2	
Adam1b	
Atp8b3	
Chrna7	
Boll	
Cacna1h	
Tssk6	
Bsg	
Sed1	
Pvrl2	
Hils1	
Tekt3	
Ldhc	
Ptpn1	
Dbil5	
Cttn	
Dpep3	
Adcy2	
Dnmt3l	
Acrbp	
Tctex1	
Tcte1	
Sept4	
Pebp1	
Gpi1	
Pgam2	
Slc25a1	
Ctsl1	
Aldoa1	
Prkaca	
Akt1	
Tssk1	
Pcsk4	
B4galt1	
Hk1s	

Protein	Positively selected sites
Spesp1	
Calm1	
Vdac3	
Vcp	
Prkcz	
Arsa	
Calca	
Spaca3	
Enkur	
Spata24	
Pik3c3	
Cox6b2	
Crisp2	
Itpr1	
Dkkl1	
Ccna1	
Pdpk1	
Atp5b	
Pfkfb4	
Ift88	
Rab14	
Dazla	
Glra1	
Adcy8	
Cyct	
Tex101	
Fkbp6	
Csl	
Ace	
Syt6	
Pik3r3	
Tssk2	
Akap110	
Glut3	
Etv5	

Protein	Positively selected sites
Akap82	
Sacy	
Gas8	
Pyk2	
Dnah12	
Ddx4	
Trim36	
Odf4	
Izumo1	150L*, 240Q*
Crisp4	
Neurl1a	
Adam3	
Syt8	
Dld	
Adam2	
H1fnt	
Pkdrej	887C*, 890M**, 1034M*, 1873F*
Tekt2	
Pmca4	
Trpc2	97R**, 102S*, 185R**, 274G**, 321A**
Svs2	45H**, 132V*, 138Y*, 255F**
Catsper4	
Catsper1	
Slo3	
Spag6	
Adam1a	117S**
Smok4a	
Cd46	46R*, 312P**
Pdha2	
Nhe1	22M*
Nhe10	37T*, 650I*, 784T*
